# The Effects of Salt Fortified with Multiple Nutrients on Health Outcomes in Children, Adolescents, and Adults: A Systematic Review and Meta-Analysis

**DOI:** 10.1016/j.advnut.2025.100567

**Published:** 2025-12-04

**Authors:** Gitanjali Lall, Michael B Zimmermann, Werner Schultink, Leila M Larson

**Affiliations:** 1 Department of Health Promotion, Education, and Behavior, University of South Carolina, Columbia, SC, United States; 2 Radcliffe Department of Medicine, University of Oxford, Oxford, United Kingdom; 3 Iodine Global Network, Orleans, ON, Canada

**Keywords:** fortified salt, hemoglobin, anemia, iron deficiency, fortification

## Abstract

Nutritional deficiencies are prevalent in populations across the world. Fortification of staple foods has been used as an alternative to supplementation to address many deficiencies. One such staple is salt, which has long been fortified with iodine, but more recently with iron, folate, and other micronutrients. Our objective was to determine the effects of fortified salt on nutritional and health outcomes among children, adolescents, and adults. We conducted a systematic review of published and unpublished literature using a pre-defined search strategy. Abstracts and full texts were screened for randomized trials, quasi-randomized trials, and pre-post-designs of double or multiple fortified salt. We calculated the weighted pooled effect sizes for the effects of fortified salt on nutritional and health outcomes. Of the 395 studies identified, 33 (including 37 intervention-control comparisons) fit our inclusion criteria. Of these comparisons, 26 studied the effects of salt fortified with iron and iodine [double fortified salt (DFS)], 2 studied the effects of salt fortified with folic acid and iodine, 1 studied the effect of triple fortified salt, 1 studied the effect of quadruple fortified salt, and 7 studied the effects of multiple micronutrient fortified salt (MMFS; fortified with ≥5 nutrients). Pooled effect sizes indicated positive effects from all iron-containing fortified salt on hemoglobin concentration [standardized mean difference (95% confidence interval): DFS 0.36 (0.22, 0.50), *n* comparisons = 26; triple fortified salt 1.56 (1.42, 1.70), *n* comparisons = 1; quadruple fortified salt 0.33 (0.02, 0.63), *n* comparisons = 1; MMFS 0.23 (0.03, 0.43), *n* comparisons = 6]. DFS and MMFS reduced the odds of anemia and iron deficiency (ID) anemia. MMFS improved serum folate and reduced the odds of ID. Pooled effects on biomarkers of vitamin B12, vitamin A, and zinc status varied by type of salt, but were largely not significant. Fortification of salt with iodine and iron, with and without other nutrients, is effective in increasing hemoglobin and reducing the odds of anemia and ID in population-based studies.

**Statement of significance**

Salt has long been fortified with iodine, but more recently with iron, folate, and other micronutrients. Our systematic review and meta-analysis demonstrate that fortification of salt with iodine and iron, with and without other nutrients, is effective in increasing hemoglobin and reducing the odds of anemia and iron deficiency in population-based studies.

## Introduction

Micronutrient deficiencies are common in low- and middle-income countries, particularly among females of reproductive age and children. Globally, the prevalence of anemia is 29.9% in females and 39.8% in children <5 y of age, with important consequences for growth, development, fatigue, and well-being [1]. The etiology of anemia is complex and is commonly related to nutritional deficiencies, particularly iron deficiency (ID), but also includes deficiencies in other key nutrients, such as zinc, folate, and vitamin B12 [2]. Inadequate intake of micronutrients is a global issue. For instance, insufficient consumption of iodine is estimated in >68% of the global population, iron in 65% of the population, and folate in 54% of the population [3]. Attempts to address these deficiencies have included diet diversification, biofortification of staple crops, micronutrient supplementation, and large-scale fortification with nutrients such as iron, zinc, folate, vitamin B12, vitamin A, and iodine [4]. Fortification of commonly consumed staple foods can be offered over long periods of time to help improve nutrient status and minimize risks associated with high intakes of nutrients (e.g. iron).

Salt has emerged as a vehicle for fortification that is appropriate in many contexts globally. Important strides have been made in the ability to fortify salt with nutrients beyond iodine, including iron, folic acid, vitamin B12, and others. Recent meta-analyses have demonstrated the benefits that salt fortified with iron and iodine [i.e. double fortified salt (DFS)] can have on hemoglobin, iron status, anemia, and ID [5,6]. Literature, however, is limited on the health benefits of fortification of salt with other micronutrients, including folic acid, vitamin B12, and others. This review is critical for several reasons. First, new studies of DFS have been published, which necessitate an update to the last review. Second, several recent publications have reported the health effects of salt fortified with iodine and nutrients other than iron, which must be reviewed in the context of other evidence on salt fortification. This systematic review and meta-analysis sought to determine the effects of fortified salt on nutritional and health outcomes among children, adolescents, and adults.

## Methods

### Search strategy and selection criteria

In this systematic review and meta-analysis, we included randomized trials, quasi-experimental study designs, and non-experimental pre-post designs reported in English. We did not restrict the search to a particular population type and included studies that enrolled infants, children, adolescents, or adults. We included interventions using salt fortified with iodine and ≥1 other nutrient (i.e. double or multiple fortified salt). Comparison groups may have received no intervention or iodized salt only. No date restrictions were applied. We reported on outcomes of nutritional biomarkers (i.e. hemoglobin, anemia, iron status, iodine status, folate status, and vitamin B12 status), child development, work productivity, and blood pressure. We examined outcomes measured immediately after the intervention period.

We searched MEDLINE (Ovid), Embase (Ovid), CINAHL (EBSCO), Cochrane Central Register of Controlled Trials, and ProQuest from database inception until 9 October, 2024. We also examined the references of included studies and relevant reviews to search for further studies that met the inclusion criteria. The search strategy used the following search terms: (“double fortified salt” OR “dual fortified salt” OR “dual salt” OR “double salt” OR “quad fortified salt” OR “multiple fortified salt” OR “iodine and iron”) AND (“anaemia” OR “anemia” OR “iron” OR “ferritin” OR “transferrin receptor” OR “hepcidin” OR “haemoglobin” OR “hemoglobin” OR “iron deficiency anaemia” OR “iron deficiency anemia” OR “vitamin B12” OR “folic acid” OR “blood pressure” OR “BP” OR “language” OR “cognitive” OR “cognition” OR “socio-emotional” OR “mental development” OR “psychomotor” OR “motor” OR “sensorimotor” OR “intelligence” OR “IQ” OR “executive function” OR “memory” OR “attention” OR “learning” OR “information processing” OR “literacy” OR “reading” OR “math” OR “school readiness” OR “emotion” OR “productivity”) AND (“trial” OR “intervention” OR “RCT” OR “program” OR “effectiveness” OR “randomized” OR “experimental” OR “difference in difference” OR “double difference” OR “instrumental variables estimation” OR “propensity score matching” OR “regression discontinuity design”). The review was registered with the PROSPERO (CRD42024587050).

### Data extraction

The review of articles for inclusion was done using Covidence. GL and LML independently screened the title and abstract of each article identified by the search strategy. Abstracts were screened for inclusion criteria based on the study design (i.e. experimental and non-experimental) and the intervention (i.e. salt fortified with iodine and ≥1 other nutrient). Full texts of articles identified through the abstract review were assessed for all inclusion and exclusion criteria.

Data were extracted from each study that met our inclusion criteria, including the following information: study country, study design, intervention and duration, type of salt and nutrients included in the fortified salt, iron concentration in salt, fortified salt stability and organoleptic properties, coverage of intervention, mean salt intake in the participants, baseline hemoglobin, ferritin, anemia, iron deficiency anemia (IDA), and morbidity, endline values for outcomes of interest, and quality ratings. Quality was assessed, and each study was assigned a global rating using the Effective Public Health Practice Project quality-assessment tool [7]. Any discrepancies in data extraction between the reviewers were resolved with discussion and by returning to the full text articles. Authors of studies with missing information were contacted twice, requesting additional information. Two authors replied with additional information. Studies were excluded if no response to inquiries was received or if the authors did not provide sufficient information to meet the inclusion criteria.

DFS was categorized into types [8]. Henceforth, type 1a DFS refers to DFS that contains microencapsulated potassium iodide and ferrous fumarate; type 1b contains encapsulated ferrous fumarate; type 2 contains a refined iodized salt, ferrous sulfate, and a stabilizing compound; type 3 contains ferrous sulfate with various chelating agents and encapsulated iodine; type 4 contains encapsulated ferrous sulfate; and type 5 contains micronized ferric pyrophosphate.

### Data analysis

We presented continuous outcomes as Hedges’ g, calculated with a pooled SD and with a 95% confidence interval (CI) [9]. Odds ratios (OR) were calculated for all dichotomous outcomes. Pooled analyses were conducted separately for DFS (salt fortified with iron and iodine), salt fortified with folic acid and iodine (FISFA), triple fortified salt (TFS, salt fortified with iron, vitamin A, and iodine), quadruple fortified salt (QFS, salt fortified with iron, folic acid, vitamin A, and iodine), and multiple micronutrient fortified salt (MMFS, salt fortified with iron, folic acid, vitamin A, iodine, and other nutrients). This was done primarily because our study was aimed at informing programmatic and policy decisions about the use of different fortified salt formulations. Effect sizes and 95% CIs were calculated for urinary iodine and some ferritin concentrations from median and range values using a method presented by Hozo et al. [10]. Weights were assigned to each study by calculating the inverse variance of the endline scores. Pooled effect sizes and ORs for all outcomes were calculated by taking a weighted average of included studies. If studies reported outcomes for multiple population groups, a weighted average of all groups was used for the main analysis. For non-experimental studies (e.g. single-group pre-post study designs), the baseline values were used as the comparison.

Sensitivity analyses were conducted by analyzing stratified pooled effect sizes and risk ratios by study quality, DFS formulation (as described above [8]), population group, average iron intake from fortified salt, intervention duration, baseline hemoglobin concentration, baseline anemia prevalence, and study design (efficacy compared with effectiveness, excluding non-experimental study designs).

Most DFS studies examined a single type of DFS, with the exception of Andersson et al. [11] and Ramachandran et al. [12]. Andersson et al. [11] reported effects using 2 intervention groups, 1 receiving DFS with micronized ferric pyrophosphate and another with encapsulated ferrous fumarate. Ramachandran et al. [12] reported effects of 2 intervention groups, 1 receiving DFS with encapsulated ferrous fumarate (type 1b) and the other receiving DFS with ferrous sulfate heptahydrate (type 2). Goh et al. [13] reported effects using 2 types of MMFS, 1 with encapsulated ferrous fumarate, zinc, vitamin B12, folic acid, and iodine, and the other with ferric pyrophosphate plus EDTA, zinc, vitamin B12, folic acid, and iodine. In these 3 studies, both intervention groups were compared with the same control group. In our analysis, the intervention groups in these studies were included as separate comparisons, and the control group *n* was halved in pooled effect size calculations to avoid over-weighting the studies.

Data analyses were conducted using R V.4.3.2 (R Foundation for Statistical Computing), and forest and funnel plots were created using the metafor package. Statistical heterogeneity was assessed using the χ^2^ test on Cochrane’s heterogeneity statistic Q and the I2 statistic. Because of heterogeneity between studies, random effects models were used to create the pooled effect sizes and risk ratios. Publication bias was examined by generating funnel plots.

### Role of the funding source

The funders of the study had no role in study design, data collection, data analysis, data interpretation, or writing of the report. The corresponding author had full access to all the data in the study and had final responsibility for the decision to submit for publication.

## Results

**FIGURE 1:**
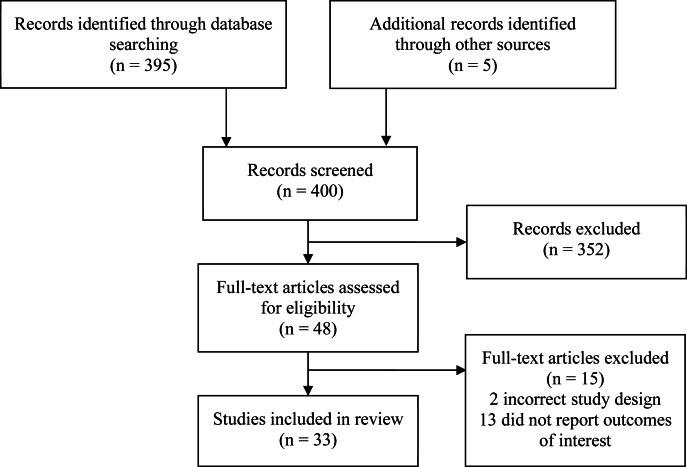
Study inclusion flow diagram.

We identified 395 studies through the search strategy and other sources (e.g. review of other meta-analyses and systematic reviews). Of these, 48 titles and abstracts were considered relevant. After a full text review, 33 studies fit our inclusion criteria (Figure 1). Four studies [[11], [12], [13], [14]] included 2 intervention groups; therefore, our review included 37 comparisons (i.e. intervention compared with comparison group). Of these, 26 studied the effects of salt fortified with iron and iodine (DFS), 2 studied the effects of salt FISFA, 1 studied the effects of TFS, 1 studied the effects of QFS, and 7 studied the effects of MMFS. We only analyzed pooled effect sizes for salt formulations with ≥2 comparisons; therefore, analyses of pooled effects focused on DFS, FISFA, and MMFS.

The majority of studies were conducted in India (*n* = 24); others were conducted in Morocco (*n* = 3), Ghana (*n* = 2), Sri Lanka (*n* = 1), Cȏte d’Ivoire (*n* = 1), Tanzania (*n* = 1), and the United States (*n* = 1). Five studies examined children <5 y of age, 19 studies examined school-age children (SAC) and adolescents (including 1 study which examined children aged 1‒15 y), 11 studies examined adult females, 3 examined pregnant females, 1 examined lactating females, and 4 examined adult females and males. Different types of designs were observed: 22 reported results from efficacy trials, 3 from randomized effectiveness trials, 1 from nonrandomized quasi-experimental designs, and 7 from pre-post designs. Twenty-five studies reported endline hemoglobin concentrations, 14 reported ferritin concentration, 15 reported urinary iodine concentration, 4 reported zinc protoporphyrin (ZnPP), 11 reported soluble transferrin receptor (sTfR), 5 reported serum folate, 9 reported body iron concentration, 4 reported serum retinol, 2 reported serum vitamin B12, 3 reported serum zinc, 16 reported anemia prevalence, 9 reported IDA prevalence, 5 reported ID, 2 reported various measures of cognition, and 2 reported work productivity using a variety of measures. Many studies had a global quality rating of weak (*n* = 20); only 5 studies were rated as moderate and 8 as strong (TABLE 1, TABLE 2 [[11], [12], [13], [14], [15], [16], [17], [18], [19], [20], [21], [22], [23], [24], [25], [26], [27], [28], [29], [30], [31], [32], [33], [34], [35], [36], [37], [38], [39], [40], [41], [42]]). We do not suspect publication bias within the included studies. The funnel plots did not indicate that effect sizes are 1-sided (Supplemental Figures 1‒23).

**TABLE 1:** Summary of study characteristics.^1^.

Author and year	Country	Design and intervention	DFS type and iron concentration	Stability and organoleptic properties	Iodine loss from storage
Double fortified salt (salt fortified with iron and iodine)					
Asibey-Berko et al. [19] 2007	Ghana	Randomized efficacy: double-blind randomized controlled efficacy trial in the agrarian region of Sekyere West district of Ghana, in which mildly anemic or non-anemic females and their mildly anemic and non-anemic children were enrolled and received either DFS or iodized salt.	Type 1a; 1000 ppm iron	DFS found to be stable after 12 mo of storage. The DFS was slightly darker in color compared with iodized salt. However, 2.7% of females in the DFS group reported darkening of plantain when fried with DFS. The food taste did not change and remained acceptable.	—
Nti-Nimako, [25] 1998	Ghana	Randomized efficacy*:* double-blind randomized controlled efficacy study in children with Hb ≥10 g/dL from 9 communities in the Sekyere West District who received DFS or iodized salt.	Type 1a; 1000 ppm iron	No differences in taste, but lower appearance and acceptability for DFS.	—
Nair et al. [26] 2013	India	One group pre-test post-test: pre-post design study in 11 randomly selected villages in rural Vadodara. Three thousand one hundred twenty-five children were examined for goiter and urinary iodine to determine eligibility. Included only children with goiter and low urinary iodine excretion. Only the intervention children were followed up.	Type 1a; 1000 ppm iron	—	—
Andersson et al. [11] 2008	India	Randomized efficacy: double-blind randomized controlled efficacy trial in 18 villages in Bangalore, children recruited from 6 schools, 5 primary schools, and 1 high school, and randomly assigned to DFS or iodized salt.	Type 5; 2000 ppm iron	Negligible color difference between DFS (light beige) and IS. No detectable difference in color, odor, or taste. Rated as acceptable.	Iodine stability testing was performed on the 2 different types of native salts used in the trial, salt with 0.5% moisture and with 1.8% moisture; the IS, MGFePP, and EFF salts of the 2 different qualities were locally stored as 2.5-kg batches in closed, high-density, transparent polyethylene bags that were stored indoors under local ambient conditions and out of direct sunlight. Iodine losses in the MGFePP salt were 44% over the first month of storage and 86% >6 mo of storage in salt with 1.8% moisture.
			Type 1b; 1000 ppm iron	Slight color difference between DFS (light gray) and IS. When added to the water used in cooking rice, EFF produced small black spots on the surface of the cooked rice grains. Rated as acceptable.	There was no difference in iodine stability between the EFF and the IS: both salts lost 20% of their iodine content after 6 mo.
Jayatissa et al. [18] 2012	Sri Lanka	Randomized efficacy: cluster randomized community efficacy trial in rural and urban areas of Gampaha in which communities were randomly assigned to receive DFS or iodized salt.	Type 1b; 1000 ppm iron	The main problem identified relevant to the low acceptability of DFS was the change in color, and this was identified as a decisive factor while purchasing salt. However, 5.9% of households in the DFS group, compared with 2.6 % in the iodine group, complained about the color of the salt.	—
Haas et al. [27] 2014	India	Randomized efficacy: double-blind randomized controlled efficacy trial in healthy non-pregnant females who worked as full-time tea pickers in the Darjeeling district of India and were randomly assigned to DFS or iodized salt. Albendazole was administered to all eligible participants 4 wk before baseline and at midline.	Type 1b; 1100 ppm iron	—	—
Banerjee et al. [28] 2018	India	Randomized effectiveness: first experiment made DFS available in shops and the public distribution system at a reduced price (9 rupees) in 200 randomly selected villages in Bihar. The control group received nothing (DFS was not available for sale).	Type 2; 1000 ppm iron	—	—
		Randomized effectiveness: second experiment was embedded in the first sales experiment. In 62 villages where DFS was being sold in shops, a regular supply of free DFS was distributed to a random subset of homes.	Type 2; 1000 ppm iron	—	—
Sivakumar et al. [24] 2001	India	Randomized efficacy: unblinded randomized efficacy trial where 4 blocks in villages in the tribal areas of East Godavari district (Andhra Pradesh) were randomly assigned to receive either DFS, iron-fortified salt, iodized salt, or unfortified salt. Here, we only present DFS vs. unfortified salt findings. Cross-sectional samples of individuals were tested at baseline and endline (not necessarily following up the same individuals).	Type 2; 1000 ppm iron	Acceptability of DFS was tested by assessing knowledge, attitude, and practices. No complaints of any side effects from the consumption of DFS.	The stability of iodine in DFS, when tested by spot test every 3 mo under the operational conditions prevailing at these tribal households, was >15 ppm and therefore at an acceptable level.
Krämer et al. [29] 2018	India	Randomized effectiveness: randomized effectiveness trial in second-grade children in Bihar assigned to receive DFS-fortified food through the mid-day meal school feeding program. Fifty-four schools were assigned to receive the DFS at a subsidized price, and the 54 control schools received regular salt.	Type 2; 1000 ppm iron	Not described, but laboratory studies have shown good stability of the iron and iodine content of the NIN formula of DFS.	—
Reddy and Nair, [15] 2014	India	Randomized efficacy: cluster randomized efficacy trial in school children from rural villages of Vadodara, Gujarat. Two schools were assigned to the DFS intervention, and 2 schools to the control group (received nothing but were recommended to continue consuming iodized salt). De-worming is given to half of the intervention and half of the control children.	Type 2; 1000 ppm iron	—	—
Sivakumar et al. [24] 2001	India	Randomized efficacy: double-blind efficacy RCT where 4 residential schools were randomly assigned to receive either DFS or iodized salt.	Type 2; 1000 ppm iron	—	The iodine content of DFS collected from the residential schools was below acceptable limits (<15 ppm) in the first and the third batch; 2 reasons cited for this potential iodine loss in DFS were *1*) salt supplier not maintaining good quality control at the production level (magnesium and insoluble solid content particularly exceeded prescribed limits); *2*) the bulk packing of the fortified salt supplied for the school (50-kg packets) as against 1-kg packets of iodine (used in the tribal households study)
Bathla and Grover, [30] 2017	India	Efficacy*:* efficacy study in anemic adolescents attending government schools in Ludhiana District (Punjab) who received DFS or iodized salt. No description of random allocation or if a random design.	Type 2; 1000 ppm iron	—	—
Reddy and Nair, [31] 2016	India	Randomized efficacy*:* randomized efficacy trial in pregnant females recruited in their first trimester from a semi-government hospital of urban Vadodara, Gujarat, to receive DFS or nothing (but recommended to continue consuming iodized salt).	Type 2; 1000 ppm iron	—	The stability of DFS (with mean iodine content 40 ppm and mean iron content 1050 ppm) was assessed after 1 y; the contents remained close to the recommended levels for iodine (37.5 ppm) and iron (979 ppm). In this study, iodine in DFS was apparently more stable and provided more iodine compared with iodized salt.
Nair et al. [32] 2014	India	One group pre-test post-test: pre-post design study in critically anemic pregnant females living in the tribal areas of Jhagadia. No comparison group. Females supplemented with DFS and IFA.	Type 2; 1000 ppm iron	—	—
Vinodkumar et al. [33] 2007	India	Randomized efficacy*:* single blind randomized efficacy trial in 7 centers in 3 states in India, where individuals were randomly assigned to receive either DFS or iodized salt. All participants were dewormed at baseline, after 6 mo, and at endline.	Type 3; 1000 ppm iron	No color changes in the DFS during transport and storage for 2 y. Iron was found to be stable. No complaints regarding taste, but the food turned slightly sour when kept for >6 h (determined using questionnaires to heads of households and the females in families).	The iron and iodine in the DFS were stable during storage for 2 y - no significant difference (*P* > 0.05) between the stability of iodine in DFS and in iodized salt.
Rajagopalan et al. [17] 2000	India	Randomized efficacy*:* double-blind randomized efficacy trial where DFS was distributed to half the adult males and females tea plantation workers, and common unfortified salt was distributed to the other half for 12 mo. The tea estate is isolated and far from the city; therefore, participants could be closely monitored, and access to non-study salt was difficult.	Type 3; 1000 ppm iron	No change in color, taste, or appearance of the food when cooking with DFS.	Samples were collected from the kitchens of the end user and tested for the stability of iron and iodine; iron and iodine were stable in these samples for more than a year.
Zimmermann et al. [22] 2002, 2003	Morocco	Randomized efficacy*:* double-blind efficacy RCT in iodine-deficient 6‒15-y-old children from 2 neighboring primary schools, where the intervention group received DFS and the control group received iodized salt. Salt was dispensed monthly directly to the head of the household from a central supply at the local health center.	Type 4; 1000 ppm iron	After storage for 20 wk, the DFS and iodized salt were not significantly different in color. Stability was acceptable when the compounds were added to local meals. During the damp winter season, when the moisture content of the local salt is high (∼3%), the DFS developed a mild yellow color during storage.	There was no significant difference in the iodine content of the salts during the 20-wk period in either the dry or the damp season. In both seasons, the salts each lost ∼15% of their iodine content by 20 wk.
Zimmermann et al. [21] 2004	Morocco	Randomized efficacy*:* double-blind efficacy RCT in iodine-deficient 6‒15-y-old children from 2 neighboring primary schools, where the intervention group received DFS and the control group received iodized salt. Population with a high prevalence of anemia living in rural villages in the Brikcha Rural Commune.	Type 5; 2000 ppm iron	After storage for 6 mo, there were no significant differences in color lightness between the DFS and iodized salt. Both salts were universally used, and there were no significant differences between the DFS and the IS in acceptability of salt color, salt taste in foods, and overall acceptability.	There was no significant difference between the DFS and the iodized salt in iodine content during storage; both salts lost ∼20% of their iodine content after 6 mo.
Wegmüller et al. [34] 2006	Cȏte d'Ivoire	Randomized efficacy*:* double-blind randomized efficacy trial in iron-deficient (with or without anemia) children 5‒15 y of age in a rural village in Dabou district, where the intervention group received DFS and the control group received iodized salt. All children were dewormed at baseline.	Type 5; 3000 ppm iron	No difference in color lightness between DFS and IS. In the triangle testing comparing DFS and IS, there was no detectable difference in color, odor, or taste in either the traditional staples (rice, cassava, yam, plantain) or the sauces (tomato, eggplant, okra, palm nut).	DFS and IS were stored as 10- and 5-kg portions in loosely woven, high-density polyethylene bags typically used to package salt at the production site and as two 300-g portions in transparent low-density polyethylene bags typically used at the retail level and in markets. Iodine loss at 6 mo was ∼50% for the IS and ∼70% for the DFS when stored in low-density polyethylene bags and close to 100% for both salts when stored in high-density polyethylene bags.
Kaur, [35] 2000	India	Efficacy*:* efficacy study in 150 young females (healthy, non-pregnant, non-lactating) residing in the University Girls’ Hostel of Punjab Agricultural University, Ludhiana, who received DFS or iodized salt. All subjects were dewormed prior to enrolment. No description of a randomized design.	Not reported	—	Iodine content in DFS decreased upon storage for 6 mo in closed containers - the percent decrease was by 41.4% in polythylene containers, 24.1% in steel containers, and 20.7% in air-tight plastic containers, respectively. Significant (*P* < 0.01) decrease in iodine content of salt was observed at 4 mo of storage in all the storage containers. The iodine content of salt packed in polyethylene (13.6 ppm) decreased <15 ppm (minimum recommended concentration of iodine in salt at retail level) after 6 mo of storage, whereas it remained >15 ppm in the other 2 storage containers.
Nadiger et al. [36] 1980	India	Efficacy*:* nonrandomized trial in school-aged children where 1 school received DFS and 1 received crushed salt for 12 mo. Group hemoglobin was not comparable at baseline.	Not reported	The fortified salt was found to be acceptable in color and taste when added to diets cooked by traditional methods. Bioavailability of iron did not alter on storage under hot, humid conditions.	—
Godbole et al. [37] 2021	India	Quasi-experimental effectiveness*:* cross-sectional study conducted as a nested substudy to a larger effectiveness study of DFS distributed through the public distribution system in Uttar Pradesh. Two districts having ≥50% DFS utilization were matched with 2 comparison districts where iodized salt was freely available.	Type 1b; 850‒1100 ppm iron	—	—
Ramachandran et al. [12] 2023	India	Randomized efficacy*:* community-based randomized study to assess the impact of 2 formulations of DFS (1 fortified with ferrous sulfate and the other fortified with ferrous fumarate) compared with iodized salt for 12 mo.	Type 2; iron concentration not reported Type 1b; iron concentration not reported	10% of families did not want to use the salt allocated to the family because they did not want to consume salt that had black spots, like the color of the salt, taste (not salty enough), or oily smell of salt solution in warm water when used for gargling.	Iodine levels in the salt were satisfactory during monthly testing.
Mdoe, [14] 2023	Tanzania	Randomized efficacy*:* double-blinded 3-arm RCT with females of reproductive age (18‒49 y) with hemoglobin between 8 and 12 g/dL who were neither pregnant nor lactating. Three arms were: *1*) quadruple fortified with iodine, iron, vitamin B12, and folic acid (QFS); *2*) double fortified with iodine and iron (DFS); and *3*) iodized salt distributed for 10 mo.	Not reported	—	—
Quadruple fortified salt (salt fortified with iron, folic acid, vitamin A, and iodine)					
Mdoe, [14] 2023	Tanzania	Randomized efficacy*:* double-blinded 3-arm RCT with females of reproductive age (18‒49 y) with hemoglobin between 8 and 12 g/dL who were neither pregnant nor lactating. Three arms were: *1*) quadruple fortified with folic acid, iron (30‒35% RDI), vitamin B12, and iodine (100% RDI) (QFS); *2*) double fortified with iodine and iron (DFS); and *3*) iodized salt distributed for 10 mo.	Not reported	QFS is indistinguishable in taste, color, and smell from regular salt.	—
Salt fortified with folic acid and iodine					
Pattisapu, [23] 2024	India	One group pre-test post-test: nonrandomized trial using a preintervention and postintervention design among non-pregnant non-lactating females aged 18 to 45 y in rural South India. The intervention group received 4 mo of salt fortified with folic acid (350 *μ*g of folicacid per 10 g of iodized salt) and iodine (FISFA), the comparison group received iodized salt.	—	The study salt was acceptable in its appearance and taste, as reported during town hall meetings and door-to-door interviews.	The study salt was stable for 8 mo at room temperature in a community setting, retaining its iodine and folic acid levels on repeated testing.
Arynchyna-Smith, [38] 2024	United States	One group pre-test post-test: pre-post intervention study design (no comparison group) to examine the effectiveness of fortified iodized salt with folic acid (FISFA) (100 mcg of folic acid and 19 mcg of iodine) among non-pregnant non-lactating females of reproductive age (18‒40 y). The intervention consisted of the substitution of regular salt with a FISFA saltshaker for 1 mo to use when cooking and eating at home or dorm, and as a table salt when eating outside the home.	—	The color of the salt had the expected light yellow tint attributable to the presence of folic acid. Acceptability of FISFA was high regarding both taste and color (96.7% and 90%, respectively).	—
Triple fortified salt (salt fortified with iron, vitamin A, and iodine)					
Zimmermann, [20] 2004	Morocco	Randomized efficacy: 10 mo randomized double-blind trial in goiterous school children. Children were randomly divided by household to receive either iodized salt (i.e. salt fortified with 25 ug iodine/g salt) or TFS (i.e. salt triple-fortified with 25 ug iodine, 60 ug vitamin A, and 2 mg iron/g salt).	2000 ppm iron as micronized ferric pyrophosphate	No significant color change in the IS and TFS over 6 mo of storage. No significant difference in color, odor, or taste between the fortified salts in any of the traditional foods.	Both salts lost 15% of their iodine content after 6 mo.
Multiple micronutrient fortified salt (salt fortified with iron, folic acid, vitamin A, iodine, and other nutrients)					
Vinodkumar, [16] 2007	India	Quasi-experimental effectiveness: using a pre-post non-randomized design, 7–11-y-old school children in Chennai, India, were assigned through their schools to experimental (i.e. residential school) and control (i.e. day school) groups. The school kitchen in the experimental group cooked with salt fortified with multiple micronutrients [chelated ferrous sulfate(1000 ppm) plus microencapsulated vitamin A (300 IU/g), vitamin B1 (200 mg/kg), vitamin B2 (200 mg/kg), vitamin B6 (200 mg/kg), vitamin B12 (400 mcg/kg), folic acid (5 mg/kg), niacin (3 g/kg), calcium pantothenate (200 mg/kg) and iodine (40 ppm)] for a period of 1 y. The control group received no intervention. De-worming was provided to both groups.	1000 ppm iron as chelated ferrous sulfate	The fortified salt did not change the color or taste of any food preparation.	—
Vinodkumar, 2009 [39]	India	Randomized efficacy: Six residential schools with children 5‒18 y of age were randomly assigned to 1 of 2 groups for 9 mo. The experimental group received a multiple micronutrient-fortified salt containing vitamins A (3000 IU), vitamin B1 (1mg), vitamin B2 (1mg), vitamin B6 (1mg), vitamin B12 (4 mcg), as well as folic acid (100 mcg), niacin (5 mg), iron (1000 ppm), iodine (40 ppm), and zinc (10 mg) (per 10 g MMFS). The control group received iodized salt. De-worming was provided to both groups.	1000 ppm iron as chelated ferrous sulfate	All of the micronutrients were uniformly and homogeneously distributed within the product.	14.31%
Vinodkumar, [40] 2014	India	Randomized effectiveness: A community-based RCT in which villages were randomly assigned to 1 of 3 arms for 8 mo. Households in the first arm received multiple micronutrient fortified salt, the second arm received health education, and the third arm had no intervention (control group). De-worming was provided to all arms. Ten grams of the multiple micronutrient fortified salt contained 3000 IU of vitamin A (from microencapsulated vitamin A acetate), 10 mg of chelated iron (from chelated ferrous sulfate), 40 ppm iodine (from microencapsulated potassium iodate), 1 mcg of vitamin B12 (from microencapsulated vitamin B12), and 100 mcg of vitamin folic acid (from microencapsulated folic acid).	1000 ppm iron as chelated ferrous sulfate	All of the micronutrients were uniformly and homogeneously distributed within the product. Each micronutrient was separately microencapsulated.	The multiple micronutrients (including iodine) in the salt were stable during storage.
Vinodkumar, [41] 2020	India	Quasi-experimental effectiveness: Pre-post-test nonrandomized design to evaluate the effects of multiple micronutrient-fortified salt (10 g of the fortified salt contained 30 mg of chelated iron, 900 *μ*g of iodine, 12 *μ*g of vitamin B12, 300 *μ*g of folic acid, and 30 mg of zinc). Children who consumed the noon meal at school constituted the intervention group, and they consumed the fortified salt for 1 y. Children who did not consume the noon meal and brought their own lunches from home formed the reference group.	3000 ppm iron as chelated ferrous sulfate	All of the micronutrients were uniformly and homogeneously distributed within the product. Each micronutrient was separately microencapsulated.	6% >12 mo.
Vinodkumar, [42] 2021	India	Randomized efficacy: RCT in which villages were randomly assigned to 1 of 2 groups. Households in the experimental villages received multiple micronutrient fortified salt (10 g of the fortified salt contained 10 mg of chelated iron, 400 *μ*g of iodine, 4 *μ*g of vitamin B12 and 100 *μ*g of vitamin folic acid, and 10 mg zinc) for a period of 8 mo. The households in the control villages continued to use the conventional iodized salt. De-worming was provided to both groups.	1000 ppm iron as chelated ferrous sulfate	—	3 ppm lost after 6 mo of storage; further 2 ppm lost after 12 mo of storage.
Goh, [13] 2025	India	Randomized efficacy: Community-based, randomized, controlled trial of IS vs. 2 types of MMFS: *1*) encapsulated ferrous fumarate, zinc, vitamin B12, folic acid, and iodine, *2*) ferric pyrophosphate plus EDTA, zinc, vitamin B12, folic acid, and iodine. The composition per gram of salt was as follows: 1.3 mg iron; 1.4 mg zinc (as zinc oxide); 0.6 *μ*g vitamin B12; 52 *μ*g folic acid, and 30 *μ*g iodine.	1300 ppm iron as encapsulated ferrous fumarate	Preliminary stability, sensory, and acceptability testing of the new MMFS formulations have yielded promising results.	—
			1300 ppm iron as ferric pyrophosphate		

Abbreviations: DFS, double fortified salt; EDTA, ethylenediaminetetraacetic acid; EFF, encapsulated ferrous fumarate; FISFA, fortified iodized salt with folic acid; Hb, hemoglobin; IFA, iron and folic acid; IS, iodized salt; MGFePP, micronized ground ferric pyrophosphate; MMFS, multiple micronutrient fortified salt; NIN, National Insitute of Nutrition; QFS, quadruple fortified salt; RCT, randomized controlled trial; RDI, recommended dietary intake; TFS, triple fortified salt.

1 Type 1a DFS refers to DFS which contains microencapsulated potassium iodide and ferrous fumarate; type 1b contains encapsulated ferrous fumarate; type 2 contains a refined iodized salt, ferrous sulfate, and a stabilizing compound; type 3 contains ferrous sulfate with various chelating agents and encapsulated iodine; type 4 contains encapsulated ferrous sulfate; and type 5 contains micronized ferric pyrophosphate.

**TABLE 2:** Summary of study outcomes.^1^.

Author and year	Salt intake (g/person/d)	Population group	Baseline mean Hb (g/dL) and anemia %	Sample size	Endline nutritional biomarkers, mean ± SD or median (IQR) or %	Global quality rating
Double fortified salt (salt fortified with iron and iodine)						
Asibey-Berko et al. [19] 2007	10	Children	Hb: 11.0; anemia: 26.7%	*n* INT = 23; *n* CTL = 59	Hb (g/dL): INT 11.20 ± 1.20; CTL 10.80 ± 1.50; anemia: INT 8; CTL 35	Weak
		Females	Hb: 12.6; anemia: 16.0%	*n* INT = 65; *n* CTL = 58	Hb (g/dL): INT 12.40 ± 1.30; CTL 12.3 ± 1.10; anemia: INT 23; CTL 20	
Nti-Nimako, [25] 1998	11.29	Children and adolescents	Hb: 11.1; anemia: 51%	*n* INT = 37; *n* CTL = 36	Hb (g/dL) : INT 11.30; CTL 11.00; ferritin (ug/L): INT 67.6; CTL 55.2; iodine (ug/L): INT 276; CTL 216; anemia: INT 19; CTL 36	Weak
Nair et al. [26] 2013	—	Children	Hb: 11.1	*n* Total = 3125	Hb (g/dL): INT 11.6	Weak
Andersson et al. [11] 2008	8.3	Children and adolescents	Hb: 12.5; anemia: 16.2%	*n* INT = 155; *n* CTL = 151	Hb (g/dL): INT 13.30 ± 1.20; CTL 13.00 ± 1.40; ferritin (ug/L): INT 19.7 ± 17.2; CTL 11.6 ± 12.3; iodine (ug/L): INT 166 (17, 723); CTL 355 (33, 1223); anemia: INT 7.7; CTL 14.5; IDA: INT 6.4; CTL 15.2 body iron (mg/kg): INT 3.10 (‒8.7, 8.8); CTL 1.10 (‒9.3, 6.1) ZnPP (*μ*mol/mol heme): INT 38 (20, 207); CTL 45 (22, 379) sTfR (mg/L): INT 5.8 (3.5, 30.6); CTL 6.40 (3.60,16.8)	Moderate
	8.3	Children and adolescents	Hb: 12.5; anemia: 17.1%	*n* INT = 152; *n* CTL = 151	Hb (g/dL): INT 13.40 ± 1.10; CTL 13.00 ± 1.40; ferritin (ug/L): INT 19.3 ± 15.3; CTL 11.6 ± 12.3; iodine (ug/L): INT 252 (14, 1156); CTL 355 (33, 1223); anemia: INT 5; CTL 14.5; IDA: INT 3.8; CTL 15.2 body iron (mg/kg): INT 3.20 (‒7.5, 6.8); CTL 1.10 (‒9.3, 6.1) ZnPP (*μ*mol/mol heme): INT 37 (19, 340); CTL 45 (22, 379) sTfR (mg/L): INT 5.6 (2.90, 16); CTL 6.40 (3.60, 16.8)	
Jayatissa et al. [18] 2012	—	Children	Hb: 12.3; anemia: 22.6%	*n* INT = 338; *n* CTL = 336	Hb (g/dL): INT 12.19 ± 0.92; CTL 12.08 ± 0.87; ferritin (ug/L): INT 37.14 ± 29,29; CTL 27.18 ± 22.25; iodine (ug/L): INT 164 (4, 628); CTL 134 (6, 475); anemia: INT 20.1; CTL 21.2	Weak
Haas et al. [27] 2014	12.4‒15	Females	Hb: 11.7; anemia: 53%	*n* INT = 104; *n* CTL = 108	Hb (g/dL): INT 11.70 ± 1.20; CTL 11.50 ± 1.10; ferritin (ug/L): INT 44.6 ± 30.2; CTL 39.7 ± 34.6; anemia: INT 54; CTL 68 body iron (mg/kg): INT 4.76 ± 3.29; CTL 3.79 ± 3.57 sTfR (mg/L): INT 6.42 (6.02, 6.82); CTL 6.79 (6.42, 7.16)	Strong
Banerjee et al. [28] 2018	—	All	Hb: 12.2; anemia: 44.7%	*n* Total = 34,732	Hb (g/dL): **β** ± SE: 0.033 ± 0.029; anemia: **β** ± SE: ‒0.006 ± 0.009	Weak
		Infants	—	*n* Total = 1242	Hb (g/dL): **β** ± SE: ‒0.032 ± 0.08; anemia: **β** ± SE: 0.004 ± 0.024	
		SAC		*n* Total = 12775	Hb (g/dL): **β** ± SE: 0.064 ± 0.036; anemia: **β** ± SE: ‒0.02 ± 0.013	
		Adults		*n* Total = 15576	Hb (g/dL): **β** ± SE: 0.037 ± 0.038; anemia: **β** ± SE: ‒0.007 ± 0.01	
		Elderly		*n* Total = 6295	Hb (g/dL): **β** ± SE: ‒0.041 ± 0.047; anemia: **β** ± SE: 0.021 ± 0.013	
		Females		*n* Total = 8772	Hb (g/dL): **β** ± SE: 0.016 ± 0.038; anemia: **β** ± SE: ‒0.001 ± 0.013	
	-	All	—	*n* Total = 21,623	Hb (g/dL): **β** ± SE: 0.045 ± 0.048; anemia: **β** ± SE: ‒0.015 ± 0.015	
		Infants	—	*n* Total = 780	Hb (g/dL): **β** ± SE: ‒0.018 ± 0.11; anemia: **β** ± SE: 0.015 ± 0.045	
		SAC	—	*n* Total = 7960	Hb (g/dL): **β** ± SE: 0.109 ± 0.058; anemia: **β** ± SE: ‒0.032 ± 0.02	
		Adults	—	*n* Total = 9670	Hb (g/dL): **β** ± SE: 0.032 ± 0.056; anemia: **β** ± SE: ‒0.008 ± 0.016	
		Elderly	—	*n* Total = 3925	Hb (g/dL): **β** ± SE: ‒0.122 ± 0.084; anemia: **β** ± SE: 0.006 ± 0.023	
		Females	—	*n* Total = 5455	Hb (g/dL): **β** ± SE: 0.059 ± 0.062; anemia: **β**±SE: ‒0.009 ± 0.02	
Sivakumar et al. [24] 2001	7‒9	All	—	*n* INT = 689; *n* CTL = 702	Iodine (ug/L): INT 155; CTL 97;	Weak
		Infants	Hb: 10.25	*n* INT = 360; *n* CTL = 369	Hb (g/dL): INT 11.20 ± 1.30; CTL 11.30 ± 1.69	
		SAC	Hb: 10.87	*n* INT = 232; *n* CTL = 261	Hb (g/dL): INT 11.60 ± 1.56; CTL 12.00 ± 1.67	
		Adolescents	Hb: 11.77	*n* INT = 37; *n* CTL = 32	Hb (g/dL): INT 12.52 ± 2.00; CTL 12.15 ± 2.02	
		Adolescent girls	Hb: 11.22	*n* INT = 19; *n* CTL = 24	Hb (g/dL): INT 11.40 ± 2.20; CTL 12.00 ± 1.93	
		Adolescent boys	Hb: 12.31	*n* INT = 18; *n* CTL = 8	Hb (g/dL): INT 13.70 ± 1.79; CTL 12.6 ± 2.29	
		Pregnant females	Hb: 9.36	*n* INT = 25; *n* CTL = 14	Hb (g/dL): INT 10.40 ± 1.62; CTL 9.90 ± 1.73	
		Lactating females	Hb: 10.52	*n* INT = 35; *n* CTL = 26	Hb (g/dL): INT 11.40 ± 1.16; CTL 10.9 ± 2.15	
Krämer et al. [29] 2018	—	SAC	Hb: 11.5; anemia: 45.5%	*n* INT = 726; *n* CTL = 680	Hb (g/dL): **β** ± SE: 0.14 ± 0.08; anemia: **β** ± SE: 0.09 ± 0.03	Moderate
Reddy and Nair, [15] 2014	—	Children and adolescents	Hb: 9.17; anemia: 99%	*n* INT = 442; *n* CTL = 505	Hb (g/dL): INT 9.09 ± 0.87; CTL 9.08 ± 0.91; iodine (ug/L): INT 183.37; CTL 244.57; anemia: INT 83.4; CTL 84.8	Weak
Sivakumar et al. [24] 2001	7‒9	RSAC	Hb:12.02; anemia: 39.1%	*n* INT = 448; *n* CTL = 352	Hb (g/dL): INT 11.80 ± 1.98; CTL 10.7 ± 2.07; Iodine (ug/L): INT 108 (33, 249); CTL 452 (116, 1081)	Weak
Bathla and Grover, [30] 2017	—	Adolescent girls	Hb: 9.9; anemia: 100%	*n* INT = 30; *n* CTL = 30	Hb (g/dL): INT 10.48 ± 0.80; CTL 10.33 ± 0.64; iodine (ug/L): INT 108; CTL 103; anemia: INT 90; CTL 100	Weak
Reddy and Nair, [31] 2016	10	Pregnant females	Hb: 9.40; anemia: 87.4%	*n* INT = 67; *n* CTL = 54	Hb (g/dL): INT 9.86 ± 1.00; CTL 9.15 ± 1.00; Iodine(ug/L): INT 299 (121, 493); CTL 289 (107, 783); anemia: INT 88.1; CTL 96.3	Weak
Nair et al. [32] 2014	—	Pregnant females	Hb: 4.8; anemia: 100%	—	Hb (g/dL): INT 8.3; iodine (ug/L): INT 106	Weak
Vinodkumar et al. [33] 2007	10	Children, adolescents, and adults	Hb: 10.3	*n* INT = 393; *n* CTL = 436	Hb (g/dL): INT 12.32 ± 1.93; CTL 11.06 ± 2.59; Iodine (ug/L): INT 205 (70, 600); CTL 220 (60, 600)	Weak
Rajagopalan et al. [17] 2000	10	Adults	Hb: 9.08	*n* INT = 385; *n* CTL = 408	Hb (g/dL): INT 10.19 ± 1.42; CTL 9.99 ± 1.37	Weak
		Females	Hb: 8.48	*n* INT = 230; *n* CTL = 250	Hb (g/dL): INT 10.03 ± 1.34; CTL 9.75 ± 1.32	
		Males	Hb: 9.57	*n* INT = 155; *n* CTL = 158	Hb (g/dL): INT 10.42 ± 1.53; CTL 10.30 ± 1.46	
Zimmermann et al. [22] 2002, 2003	7‒12	Children and adolescents	Hb: 11.2	*n* INT = 183; *n* CTL = 184	Hb (g/dL): INT 12.70 ± 1.20; CTL 11.60 ± 1.20; ferritin (ug/L): INT 40.0 ± 25.0; CTL 17.0 ± 12.0; iodine (ug/L): INT 189 (23, 406); CTL 182 (14, 474); IDA: INT 8; CTL 30 sTfR (mg/L): INT 6.50 (3, 15.3); CTL 8.9 (3.8, 118) ZnPP(*μ*mol/mol heme): INT 35.0 ± 24.0; CTL 57.0 ± 43.0 thyroid gland volume: INT 5.70 ± 2.10; CTL 7.30 ± 2.40	Strong
Zimmermann et al. [21] 2004	7‒12	Children and adolescents	Hb:11.5; anemia: 79.0%	*n* INT = 75; *n* CTL = 83	Hb (g/dL): INT 12.80 ± 1.10; CTL 11.50 ± 0.80; ferritin (ug/L), geometric mean ± SD: INT 33.1 ± 43.3; CTL 15.0 ± 13.1; iodine (ug/L): INT 97 (17, 1356); CTL 104 (22, 1784); anemia: INT 12; CTL 58; IDA: INT 5; CTL 29 body iron (mg/kg): INT 4.82 ± 1.94; CTL 1.08 ± 3.10 sTfR (mg/L): INT 5.8 (5.55, 6.05); CTL 7.70 (7.16, 8.24) ZnPP (*μ*mol/mol heme): INT 27.0 ± 20.0; CTL 52.0 ± 35.0 thyroid gland volume: INT 5.90 ± 2.30; CTL 6.90 ± 2.20	Strong
Wegmüller et al. [34] 2006	4‒6.1	Children and adolescents	Hb: 11.6; anemia: 52%	*n* INT = 60; *n* CTL = 63	Hb (g/dL): INT 11.70 ± 1.20; CTL 11.30 ± 1.10; ferritin (ug/L): INT 63.0 ± 57.2; CTL 61.0 ± 151.9; anemia: INT 47; CTL 62; IDA: INT 23; CTL 38 sTfR (mg/L): INT 8.20 (3, 16.10); CTL 9.20 (4.70,16.6)	Moderate
Kaur, [35] 2000	8‒10	Females	Hb: 10.9; anemia: 74.7%	*n* INT = 100; *n* CTL = 50	Hb (g/dL): INT 12.23 ± 1.20; CTL 11.41 ± 1.56; ferritin (ug/L): INT 23.1 ± 41.4; CTL 16.3 ± 25.9; anemia: INT 41; CTL 64	Weak
Nadiger et al. [36] 1980	15	SAC boys	Hb: 12.2; anemia: 50.5%	*n* INT = 222; *n* CTL = 92	Hb (g/dL): INT 13.4 ± 1.49; CTL 12.0 ± 1.44; anemia: INT 19.4; CTL 51.9	Weak
		SAC girls	Hb: 13.4; anemia: 21.1%	*n* INT = 161; *n* CTL = 71	Hb (g/dL): INT 14.8 ± 1.40; CTL 13.1 ± 1.60; anemia: INT 3.0; CTL 22.5	
Godbole et al. [37] 2021	—	Females	—	*n* INT = 1153; *n* CTL = 249	Iodine median (95% CI) (*μ*g/L): INT: 226.20 (202.30, 250.09) CTL (crystal salt): 145.70 (129.08, 162.31)	Weak
Ramachandran et al. [12] 2023	—	Males	Hb: 13.3 anemia: 35.5%	*n* INT = 220; *n* CTL = 230	Hb (g/dL): INT 11.60 ± 1.70; CTL 11.30 ± 1.70 ferritin (ug/L): INT 52.9 ± 46.55; CTL 55.20 ± 54.28	
		Females	Hb: 10.8 anemia: 77.05%			
		Children	Hb: 10.50 anemia: 71.8%			
		Males	Hb: 13.15 anemia: 42.65%	*n* INT = 214; *n* CTL = 230	Hb (g/dL): INT 11.60 ± 1.63; CTL 11.30 ± 1.70 ferritin (ug/L): INT 52.9 ± 46.55; CTL 55.20 ± 54.28	
		Females	Hb: 10.7 anemia: 79.35%			Moderate
		Children	Hb: 10.6 anemia: 70.2%			
Mdoe et al. [14] 2023	—	Females	Hb: 11.1	*n* INT = 57; *n* CTL = 55	Hb (g/dL): INT 13.03 ± 1.62; CTL 12.45 ± 1.81 ferritin (ug/L): INT 26 ± 51; CTL 10 ± 16 serum folate (nmol/L): INT: 32 ± 14; CTL: 34 ± 15 serum vitamin B12(pg/mL): INT 237 ± 157; CTL 297 ± 174	Strong
Quadruple fortified salt (salt fortified with iron, folic acid, vitamin A, and iodine)						
Mdoe et al. [14] 2023	—	Females	Hb: 11.1	*n* INT = 56; *n* CTL = 55	Hb (g/dL): INT 12.99 ± 1.49; CTL 12.45 ± 1.81 ferritin (ug/L): INT 24 ± 25; CTL 10 ± 16 serum folate (nmol/L): INT 44 ± 19; CTL 34 ± 15 serum vitamin B12 (pg/mL): INT 325 ± 181; CTL 297 ± 174	Strong
Salt fortified with folic acid and iodine						
Pattisapu et al. [23] 2024	∼300 *μ*g/d of folic acid	Females	—	*n* INT = 83; *n* CTL = 83	Serum folate (nmol/L) median (IQR): INT: 54.4 (IQR, 43.5, 54.4) CTL: 14.6 (IQR, 11.2, 20.6)	Weak
Arynchyna-Smith et al. [38] 2024	1.27	Females	—	*n* INT = 32	Serum folate (nmol/L): INT: 28.21 ± 9.30 CTL: 26.78 ± 9.66	Moderate
Triple fortified salt (salt fortified with iron, vitamin A, and iodine)						
Zimmermann et al. [20] 2004	Mean salt intake for children aged 6–14 y was 7.3–11.6 g/d	SAC	Hb: 11.5	*n* INT = 74; *n* CTL = 83	Hb (g/dL): INT 12.9 ± 1.0; CTL 11.5 ± 0.8 sTfR (mg/L): INT 5.8 ± 1.4; CTL 7.7 ± 2.4 ZnPP (*μ*mol/mol heme): INT 27 ± 19; CTL 52 ± 35 ferritin (μg/L) geometric mean (‒1 SD, +1 SD): INT 31.2 (12.8, 79.9); CTL 15.0 (6.9, 28.1) body iron (mg/kg): INT 4.69 ± 2.12; CTL 1.08 ± 3.10 IDA (%): INT 5; CTL 29 serum retinol (*μ*mol/L): INT 1.18 ± 0.12; CTL 0.91 ± 0.15 Retinol-binding protein (mg/L): INT 32.8 ± 11.1; CTL 22.4 ± 8.8 vitamin A deficiency (%): INT 1; CTL 17 low vitamin A status (%): INT 31; CTL 64	Strong
Multiple micronutrient fortified salt (salt fortified with iron, folic acid, vitamin A, iodine, and other nutrients)						
Vinodkumar et al. [16] 2007	10	SAC	Hb: 9.98	*n* INT = 63; *n* CTL = 66	Hb (g/dL): INT 10.2 ± 0.77; CTL 10.1 ± 0.75 hematocrit L/L: INT: 0.32 ± 0.03; CTL 0.29 ± 0.02 red blood cells (million/cmm): INT 3.97 ± 0.39; CTL 3.48 ± 0.26 serumretinol (*μ*g/dL): INT 41.4 ± 14.8; CTL 46.2 ± 18.6 urinary iodine (*μ*g/L): INT 510 (125, 600); CTL 70 (70, 635) angular stomatitis (%): INT 0; CTL 25.5	Weak
Vinodkumar et al. [39] 2009	10	SAC	Hb: 12.08 anemia: 93	*n* INT = 213; *n* CTL = 189	Hb (g/dL): INT 12.61 ± 1.3; CTL 12.12 ± 0.96 ferritin (*μ*g/L): INT 8.31 ± 42.12; CTL 8.13 ± 15.75 sTfR (mg/L): INT 11.74 ± 5.25; CTL 10.31 ± 3.27 body iron (mg/kg): INT ‒2.4 ± 5.23; CTL ‒2.14 ± 4.5 CRP (mg/L): INT 0.41 ± 0.72; CTL 0.57 ± 1.1 AGP (g/L): INT 0.84 ± 0.23; CTL 0.77 ± 0.22 IDA (%): INT 51.1; CTL 58.1 serum retinol (mg/dL): INT 25.34 ± 5.74; CTL19.21 ± 5.24 serum vitamin B12 (pg/mL): INT 15,741 ± 10,979; CTL 557 ± 366 serum folate (nmol/L): INT 22.93 ± 15.91; CTL 11.51 ± 5.57 serum zinc (mg/dL): INT 142.5 ± 132; CTL 102.73 ± 88.78 angular stomatitis (%): INT 4.2; CTL 16.4 anemia (%): INT 60; CTL 69.8 ID (%): INT 84.4; CTL 86	Strong
Vinodkumar et al. [40] 2014	Ranged from 7.9 g for 5 y old children and 11.4 g in 15 y old children	SAC	Hb: 11.75 anemia: 44.45	*n* INT = 215; *n* CTL = 217	Hb (g/dL): INT 12.1 ± 1.38; CTL 11.4 ± 2.01 serum retinol (*μ*g/dL): INT 28.8 ± 13.9; CTL 22.6 ± 8.77 urinary iodine (mg/L): INT 335 (100, 500); CTL 200 (100, 400) sTfR (mg/L): INT 8.21 ± 3.00; CTL 10.6 ± 3.39 ferritin (*μ*g/L): INT 49.9 ± 21.5; CTL 37.5 ± 19.0 body iron (mg/kg): INT 5.24 ± 2.51; CTL 3.26 ± 2.64 CRP (mg/L): INT 1.10 ± 1.76: CTL 1.08 ± 1.65 AGP (g/L): INT 1.17 ± 0.34; CTL 1.13 ± 0.25 serum retinol deficiency (%): INT 28.1; CTL 39.0 anemia (%): INT 32.6; CTL 46.5 ID (%): INT 32.6; CTL 46.5 IDA (%): INT 31; CTL 39.3	Strong
Vinodkumar et al. [41] 2020	2.5‒3 g of salt per day	SAC	—	*n* INT = 128; *n* CTL = 100	Urinary iodine (*μ*g/L): INT 175 (110, 317); CTL 210 (150, 285) sTfR (mg/L): INT 7.10 ± 3.25; CTL 7.18 ± 2.20 ferritin (*μ*g/L) (geometric mean ± SD): INT 22.44 ± 22.10; CTL 25.34 ± 24.50 body iron (mg/kg): INT 2.93 ± 3.91; INT 3.21 ± 3.64 CRP (mg/L): INT 0.58 ± 1.25; CTL 2.37 ± 10.84 AGP (g/L): INT 0.74 ± 0.29; CTL 0.84 ± 0.33 IDA (%): INT 41.4; CTL 35	Weak
Vinodkumar et al. [42] 2021	10	SAC	Hb: 11.53 anemia: 56.9	*n* INT = 117; *n* CTL = 95	Hb (g/dL): INT 12.4 ± 1.37; CTL 11.67 ± 1.44 serum zinc (*μ*g/dL): INT 87.67 ± 25.83; CTL 86.66 ± 28.11 urinary iodine (*μ*g/L): INT 255 (80, 565); CTL 100 (67, 360) sTFR (mg/L): INT 6.13 ± 2.42; CTL 7.73 ± 4.12 ferritin (*μ*g/L) (geometric mean ± SD): INT 38.13 ± 28.51; CTL 25.63 ± 22.31 body iron (mg/kg): INT 5.30 ± 3.06; CTL 3.17 ± 3.97 CRP (mg/L): INT 2.80 ± 5.37; CTL 1.99 ± 2.45 AGP (g/L): INT 0.87 ± 0.27; CTL 0.87 ± 0.22	Weak
Goh et al. [13] 2025	4.6	Females	Hb: 11.93 anemia: 47.9	*n* INT = 198; *n* CTL = 209	Hb (g/dL): INT 12.00 ± 1.40; CTL 12.10 ± 1.20 ferritin (*μ*g/L): INT 20.30 ± 7.54; CTL 16.90 ± 5.75 sTfR (mg/L): INT 3.80 (3.10, 5.30); CTL 4 (3.30, 5.40) anemia (%): INT 39.30; CTL 39.70 urinary iodine (*μ*g/L): INT 529 (396, 724); CTL 452 (372, 668) IDA (%): INT 23; CTL 27.3 serum folate (nmol/L): INT 22.70 (15, 32.6); CTL 12.90 (8.70, 21.80) body iron (mg/kg): INT ‒28.20 ± 8.6; CTL ‒29 ± 7.50 serum vitamin B12 (pg/mL): INT 440.38 (329.27, 597.56); CTL 303.52 (224.93, 407.86) serum zinc (mg/dL): INT 71.60 ± 12.80; CTL 71.3 ± 13.00 ID (%): INT 33.20; CTL 42.60	Strong
				*n* INT = 196; *n* CTL = 209	Hb (g/dL): INT 12.10 ± 1.30; CTL 12.10 ± 1.20 ferritin (*μ*g/L): INT 16.40 ± 5.75; CTL 16.90 ± 5.75 sTfR (mg/L): INT 4.10 (3.20, 5.70); CTL 4 (3.30, 5.40) anemia (%): INT 43.90; CTL 39.70 urinary iodine(*μ*g/L): INT 509 (382, 643); CTL 452 (372, 668) IDA (%): INT 32.8; CTL 27.3 serum folate (nmol/L): INT 24.30 (13.70, 34.60); CTL 12.90 (8.70, 21.80) body iron (mg/kg): INT ‒29.90 ± 7.9; CTL ‒29 ± 7.50 serum vitamin B12 (pg/mL): INT 426.83 (321.14, 597.56); CTL 303.52 (224.93, 407.86) serum zinc (mg/dL): INT 72.80 ± 16.20; CTL 71.3 ± 13.00 ID (%): INT 44.90; CTL 42.60	

Abbreviations: AGP, α-1 acid glycoprotein; CI, confidence interval; CRP, C-reactive protein; CTL, control; DFS, double fortified salt; ID, iron deficiency; IDA, iron deficiency anemia; INT, intervention; IQR, interquartile range; Hb, hemoglobin; RSAC, residential school-age children; SAC, school-age children; SD, standard deviation; SE, standard error; sTfR, serum transferrin receptor; ZnPP, zinc protoporphyrin.

1 Type 1a DFS refers to DFS that contains microencapsulated potassium iodide and ferrous fumarate; type 1b contains encapsulated ferrous fumarate; type 2 contains a refined iodized salt, ferrous sulfate, and a stabilizing compound; type 3 contains ferrous sulfate with various chelating agents and encapsulated iodine; type 4 contains encapsulated ferrous sulfate; and type 5 contains micronized ferric pyrophosphate.

### Effects of fortified salt on nutritional and health outcomes

#### Hemoglobin

Statistically significant pooled effect sizes were found for the effects on hemoglobin concentration from DFS [standardized mean difference (SMD) (95% CI): 0.36 (0.22, 0.50); unstandardized mean difference (MD): 0.48 (0.29, 0.66) g/dL, *n* comparisons = 26] and MMFS [SMD: 0.23 (0.03, 0.43); MD: 0.32 (0.02, 0.61) g/dL, *n* comparisons = 6]. Only 1 study of TFS and 1 study of QFS reported effects on hemoglobin, with statistically significant effect sizes [TFS, SMD: 1.56 (1.42, 1.70); MD: 1.40 (1.26, 1.54) g/dL, *n* comparison = 1] [QFS, SMD: 0.33 (0.02, 0.63); MD: 0.54 (0.23, 0.85) g/dL, *n* comparison = 1] (Table 3, FIGURE 2, FIGURE 3, FIGURE 4, FIGURE 5, FIGURE 6, FIGURE 7, Supplemental Figures 24‒40).

**TABLE 3:** Pooled and single effect sizes and odds ratios for fortified salt studies.^1^.

Outcome	*n*	Pooled effect size (95% CI) or OR (95% CI)
Double fortified salt (salt fortified with iron and iodine)		
Hemoglobin SMD	26	0.36 (0.22, 0.50)
Hemoglobin MD (g/dL)	26	0.48 (0.29, 0.66)
Serum ferritin SMD	11	0.60 (‒0.17, 1.37)
Serum ferritin MD (*μ*g/L)	11	0.22 (‒0.29, 0.73)
ZnPP SMD	4	‒0.62 (‒3.16, 1.93)
sTfR SMD	6	‒0.68 (‒1.28, ‒0.09)
Body iron stores SMD	4	0.69 (0.18, 1.20)
Serum folate SMD	1	‒0.14 (‒2.84, 2.56)
Serum vitamin B12 SMD	1	‒0.36 (‒31.18, 30.45)
Anemia OR	14	0.43 (0.28, 0.64)
Iron deficiency anemia OR	5	0.27 (0.17, 0.41)
Salt fortified with folic acid and iodine		
Serum folate SMD	2	4.94 (‒3.86, 13.75)
Triple fortified salt (salt fortified with iron, vitamin A, and iodine)		
Hemoglobin SMD	1	1.56 (1.42, 1.70)
Hemoglobin MD (g/dL)	1	1.40 (1.26, 1.54)
ZnPP SMD	1	‒0.87 (‒5.35, 3.60)
sTfR SMD	1	‒0.95 (‒1.27, ‒0.64)
Body iron stores SMD	1	1.35 (0.93, 1.77)
Serum retinol SMD	1	1.98 (1.95, 2.00)
Quadruple fortified salt (salt fortified with iron, folic acid, vitamin A, and iodine)		
Hemoglobin SMD	1	0.33 (0.02, 0.63)
Hemoglobin MD (g/dL)	1	0.54 (0.23, 0.85)
Serum ferritin SMD	1	0.66 (‒3.24, 4.57)
Serum ferritin MD (*μ*g/L)	1	14 (10.10, 17.90)
Serum folate SMD	1	0.58 (‒2.59, 3.76)
Serum vitamin B12 SMD	1	0.16 (‒32.73, 33.05)
Multiple micronutrient fortified salt (salt fortified with iron, folic acid, vitamin A, iodine, and other nutrients)		
Hemoglobin SMD	6	0.23 (0.03, 0.43)
Hemoglobin MD (g/dL)	6	0.32 (0.02, 0.61)
Serum ferritin SMD	6	0.22 (‒0.29, 0.73)
Ferritin MD (μg/L)	6	4.16 (‒1.17, 9.48)
sTfR SMD	6	‒0.19 (‒0.51, 0.13)
Body iron stores SMD	6	0.26 (‒0.09, 0.60)
Serum folate SMD	3	2.12 (1.38, 2.87)
Serum retinol SMD	3	0.45 (‒0.34, 1.24)
Serum vitamin B12 SMD	2	2.13 (‒2.08, 6.34)
Serum Zinc SMD	4	0.00 (‒0.00, 0.00)
Anemia OR	5	0.70 (0.49, 1.00)
Iron deficiency anemia OR	5	0.74 (0.52, 1.05)
Iron deficiency OR	5	0.70 (0.53, 0.94)

Abbreviations: CI, confidence interval; MD, mean difference; *n*, refers to the number of comparisons; OR, odds ratio; SMD, standardized mean difference; sTfR, serum transferrin receptor; ZnPP, zinc protoporphyrin.

1 Pooled effects were calculated when ≥2 comparisons were available. If only 1 comparison was reported for an outcome, the effect size for that 1 comparison was used.

**FIGURE 2:**
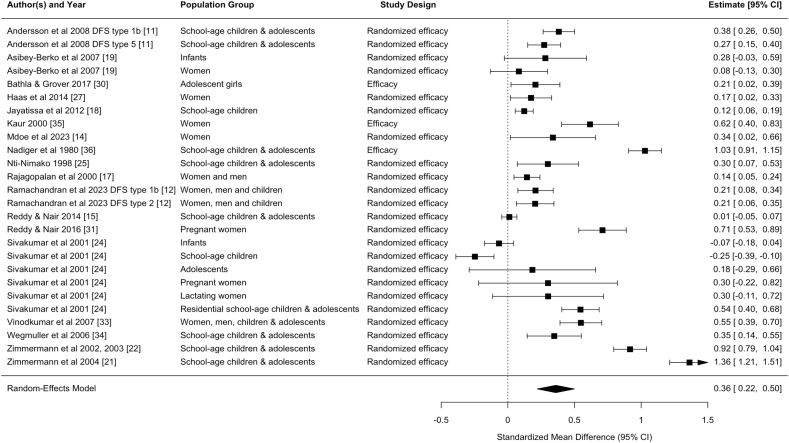
Forest plot for the effect of double fortified salt on hemoglobin concentration (standardized mean difference). In certain rows, study characteristics were added after the author and year in order to distinguish studies from each other. CI, confidence interval; DFS, double fortified salt.

**FIGURE 3:**
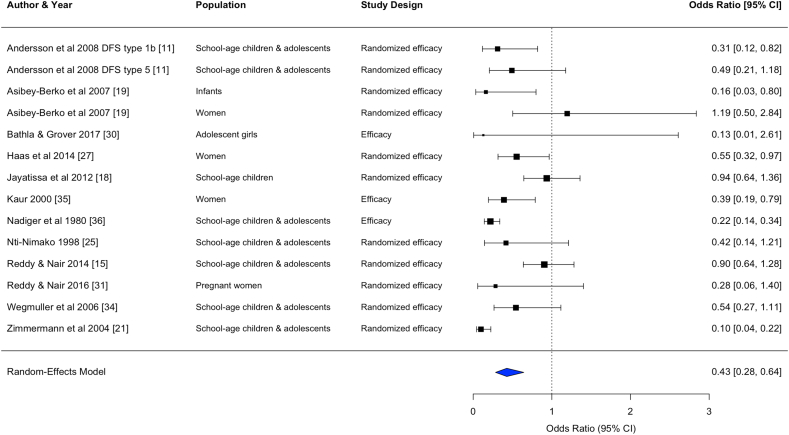
Forest plot for the effect of double fortified salt on anemia (odds ratio). In certain rows, study characteristics were added after the author and year in order to distinguish studies from each other. CI, confidence interval; DFS, double fortified salt.

**FIGURE 4:**
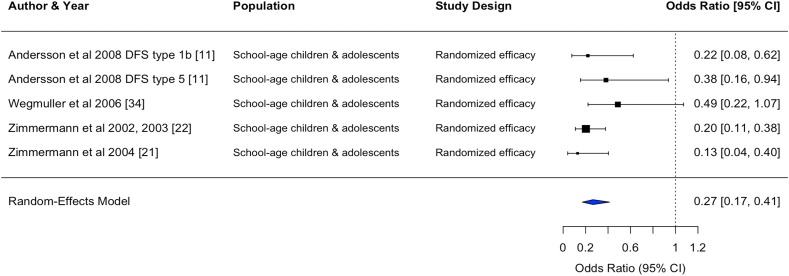
Forest plot for the effect of double fortified salt on iron deficiency anemia (odds ratio). In certain rows, study characteristics were added after the author and year in order to distinguish studies from each other. CI, confidence interval; DFS, double fortified salt.

**FIGURE 5:**
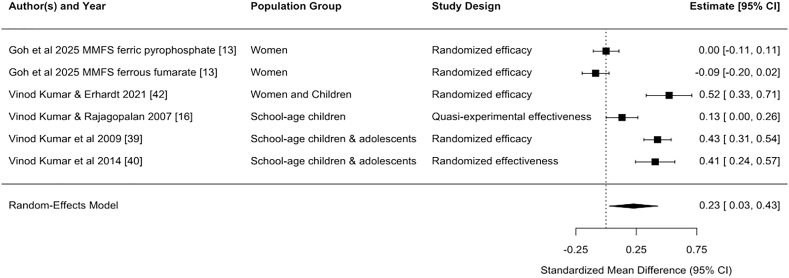
Forest plot for the effect of multiple micronutrient fortified salt on hemoglobin (standardized mean difference). In certain rows, study characteristics were added after the author and year in order to distinguish studies from each other. CI, confidence interval; MMFS, multiple micronutrient fortified salt.

**FIGURE 6:**
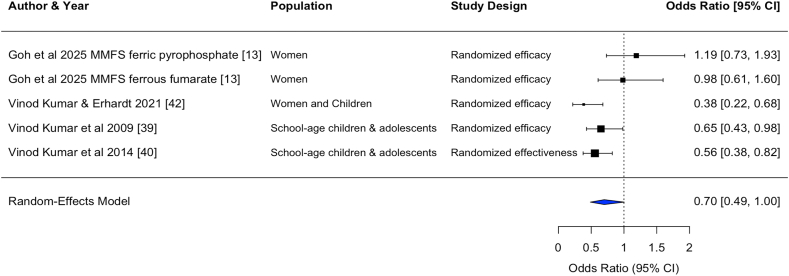
Forest plot for the effect of multiple micronutrient fortified salt on anemia (odds ratio). In certain rows, study characteristics were added after the author and year in order to distinguish studies from each other. CI, confidence interval; MMFS, multiple micronutrient fortified salt.

**FIGURE 7:**
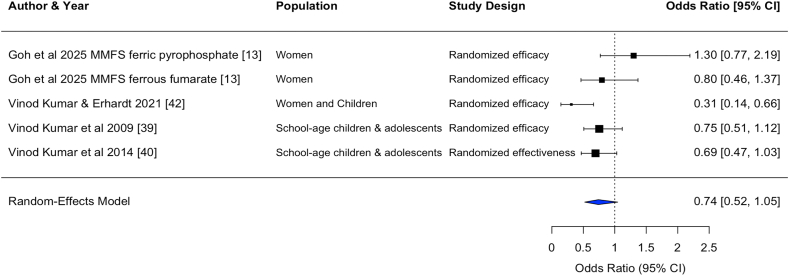
Forest plot for the effect of multiple micronutrient fortified salt on iron deficiency anemia (odds ratio). In certain rows, study characteristics were added after the author and year in order to distinguish studies from each other. CI, confidence interval; MMFS, multiple micronutrient fortified salt.

#### Iron status

Statistically significant pooled effect sizes were found for the effects of DFS on body iron stores [SMD: 0.69 (0.18, 1.20), *n* = comparisons = 4] and sTfR [SMD: ‒0.68 (‒1.28, ‒0.08), *n* comparisons = 6]. There was no statistically significant effect of DFS on ZnPP or ferritin. There was a statistically significant effect of TFS on transferrin receptor [SMD: ‒0.95 (‒1.27, ‒0.64), *n* comparisons = 1]. There was no effect of TFS on ZnPP nor of QFS on serum ferritin. Pooled effect sizes were not statistically significant for the effects of MMFS on serum ferritin, sTfR, and body iron (*n* comparisons = 6).

#### Anemia, ID, and IDA

DFS and MMFS reduced the odds of anemia [DFS, OR: 0.43 (0.28, 0.64), *n* comparisons = 14; MMFS, OR: 0.70 (0.49, 1.00), *n* comparisons = 5] and reduced the odds of IDA [DFS, OR: 0.27 (0.17, 0.41), *n* comparisons = 5; MMFS, OR: 0.74 (0.52, 1.05), *n* comparisons = 5]. MMFS also reduced the odds of ID [OR: 0.78 (0.57, 1.06), *n* comparisons = 6].

#### Folate status

One QFS intervention, 2 FISFA interventions, and 3 MMFS interventions measured effects on serum folate concentration. Effect sizes for QFS and FISFA were not significant. The pooled effect size for the effect of MMFS on serum folate was statistically significant [SMD: 2.12 (1.38, 2.87), *n* comparisons = 3].

#### Vitamin B12 status

Only 1 QFS intervention and 3 MMFS interventions examined effects on serum vitamin B12 concentrations. None of the pooled effects were statistically significant.

#### Vitamin A status

Only 1 TFS and 3 MMFS studies examine effects on serum retinol, with TFS showing a statistically significant positive effect and MMFS showing no significant effect.

#### Zinc status

There was no effect of MMFS on serum zinc levels (*n* comparisons = 4).

#### Cognition

Meaningful positive effects on cognitive development were reported in 2 trials of DFS and 1 of MMFS; however, assessment measures were not similar enough to pool results. In female tea pickers in India, positive effects were observed on perception, attention, and memory among those who received DFS compared with iodized salt [43]. In another study with school children in India, those assigned to receive DFS compared with no intervention had better outcomes on tests of memory and cognition, although only 4 schools were randomly assigned [15]. In a study involving a school meal program, children from schools that received the MMFS for 1 y had larger improvements in memory and attention, but not overall intelligence scores, compared with their counterparts from schools that did not receive MMFS [16]. No study reported effects of fortified salt on mental or motor development in young children.

#### Work productivity

Two efficacy studies measured the effects of DFS on work productivity. In Indian female tea pickers who received DFS compared with iodized salt, effects were observed on work output in terms of leaves picked [44]. In another trial in adult females and males, tea pickers in India, the average daily quantity of tea leaves picked by an individual increased in those who received DFS compared with those who received unfortified salt [17].

### Stratified analyses

Stratified analyses were exploratory, and results should be taken with some caution, given the small number of comparisons in each stratum (Supplemental Table 1‒5).

#### Study quality rating

Pooled effect sizes on iron biomarkers were larger among DFS studies with quality ratings of moderate or strong compared with weak. Among MMFS studies, effects on iron biomarkers were larger in lower compared with higher quality studies, but only 1‒2 studies contributed to the estimates for studies rated as weak.

#### Type of DFS

Effects of DFS on hemoglobin and serum ferritin were generally larger among studies that used type 4 or type 5 DFS, although effects were significant among studies of DFS type 1a, type 1b, and type 2.

#### Population

Effects of DFS on hemoglobin were larger among studies of SAC and adolescents, non-pregnant females, and pregnant females. Evaluations of MMFS were limited to populations of SAC and adolescents, and non-pregnant females, with larger effects on hemoglobin, anemia, and ID among studies of children and adolescents.

#### Iron intake from fortified salt

Effects of DFS and MMFS on hemoglobin, ferritin, and IDA were larger among studies with average iron intakes above compared with <10 mg iron per person per day.

#### Intervention duration

Although studies with durations <12 mo tended to have larger effects on ferritin and anemia for DFS, and larger effects on hemoglobin, ferritin, anemia, and IDA for MMFS, few studies had intervention durations of ≥12 mo.

#### Baseline hemoglobin and anemia status

Effects of DFS did not differ meaningfully based on the study populations’ mean baseline hemoglobin (<11 g/dL compared with ≥11 g/dL) or baseline prevalence of anemia (≤50% compared with >50%). Effects of MMFS on hemoglobin, anemia, and IDA were larger among studies with baseline anemia prevalence >50%.

#### Study design

The 2 effectiveness studies that used DFS did not report outcome measure data that could be used in the pooled analyses. Within MMFS studies, there were no meaningful differences in outcomes between efficacy and effectiveness studies.

## Discussion

In our systematic review and meta-analysis of double and multiple fortified salt on nutritional and functional outcomes, we found that DFS, TFS, QFS, and MMFS improved hemoglobin concentration, DFS and MMFS reduced the odds of anemia and IDA, and MMFS improved serum folate concentration and reduced the odds of ID. Benefits were not observed on biomarkers of vitamin B12, vitamin A, and zinc. Of the few studies that examined functional outcomes, DFS and MMFS showed benefits to cognition, and DFS improved work productivity among Indian tea pickers. The magnitude of effect varied by study characteristics, with larger effects among studies of higher quality (for DFS), children and adolescents (for DFS and MMFS), higher average iron intakes (for DFS and MMFS), and baseline anemia >50% (for MMFS). Our findings are particularly important in the context of growing micronutrient deficiencies, including iodine deficiency, in parts of the world [45].

Previous reviews have systematically documented the evidence for effects of DFS [5,8]. Our review extends these studies by reviewing the most recent DFS studies and the magnitude and direction of our results for effects on iron biomarkers complement the previous findings. No review, however, has systematically summarized the effects of multiple fortified salts besides DFS. Our review documents an emerging literature base for the impacts of multiple fortified salts, which is particularly evident for MMFS, but also includes QFS and FISFA. Some used rigorous randomized trial designs across diverse populations [13]. Many studies used less rigorous study designs, such as quasi-experimental and pre-post single-group designs, and some had minimal documentation around their fortification formulation. Overall, the recent efficacy trials of multiple fortified salts point toward benefits to nutrient status in school-aged children and females, in particular hemoglobin concentration for TFS, QFS, and MMFS, and folate for MMFS, but further high-quality studies are needed. The improvements in folate concentration for MMFS among females are meaningful in the face of a continued high prevalence of neural tube defect-affected birth outcomes globally, estimated at 18.6 of 10,000 live births, many of which are preventable with folic acid supplementation [46].

The majority of the evidence for the impacts of double and multiple fortified salt on anemia and hemoglobin originates from India. These findings are informative for adult and child populations across the country and likely generalizable to other parts of the region; however, the etiology of anemia is complex. Even among the studies in India, improvements in hemoglobin and anemia were inconsistent. At a global level, it is estimated that 50% of anemia is caused by IDA [47]. Research in India has indicated that these estimates can be >70% [48]. Given, however, that the etiology of anemia is multifactorial and varies by setting and population, the proportion of anemia that is amenable to iron, for instance, from iron-fortified salt, can be difficult to estimate and is complicated when the prevalence of other determinants (particularly other nutrient deficiencies, immune function-related causes, and genetic hemoglobin disorders) is high. For this reason, it is critical to first understand the population’s potential to respond to iron interventions. In populations that face multiple nutrient deficiencies, MMFS may be an appropriate intervention, given that it includes 5 different micronutrients (iron, zinc, vitamin B12, folic acid, and iodine).

Effectiveness trials of multiple fortified salts are currently lacking. These types of studies are critical to understand the successes and challenges with implementation from various delivery channels and what health benefits can be expected within these constraints. Evidence from efficacy trials points to benefits on overall hemoglobin concentration across populations with a wide range of baseline hemoglobin and anemia prevalence; however, these benefits can only be expected if effectiveness approaches efficacy. It is worth noting that, although many studies reported baseline anemia prevalence, few reported baseline prevalence of other nutrient deficiencies, information which plays an important role in the population’s potential to benefit and the health effects observed. An important consideration when moving from efficacy to effectiveness is how to achieve equitable and high coverage and consumption of the product. With products such as double and multiple fortified salt, coverage and consumption are largely influenced by the organoleptic properties of the fortified salt [[49], [50], [51]], which vary by type of salt, how the target population may cook and prepare their food with the salt, quality of salt, and storage conditions for the fortified salt [5]. For instance, discoloration of the fortified salt itself and the food after using the salt, and changes in food taste and smell from using the fortified salt have been reported with some types of DFS [11,12,18,19], but rarely with FISFA, TFS, QFS, and MMFS. Another consideration is how to ensure stable nutrient content in the salt. For instance, studies have reported losses of iodine content in their fortified salt of ≤15% over 6 mo [[20], [21], [22]]. Lastly, unless subsidized, the cost increment of multiple fortified salts may make these products prohibitively expensive for low-income populations and those who could benefit from them most. Depending on the context, producers may reject the production and commercial sale of multiple fortified salts as a cost-effective business model [52].

Only 5 studies included in our review reported data on morbidity or other health risks (i.e. alterations in calcium and phosphorus homeostasis, hyperthyroidism, headache, fever, stomach pain, nausea, or vomiting), but these adverse outcomes were not found to be related to the fortified salt [14,19,20,23,24]. Large-scale food fortification with micronutrients poses a risk of excess intakes of these micronutrients, which is particularly concerning when it comes to nutrients that have small safety margins, such as retinol and folate, and nutrients associated with important health concerns [53]. For instance, excess consumption of iron has been shown to decrease child growth, increase illness such as diarrhea, alter gut microbiota toward pathogenic bacteria, and increase inflammation [54]. Monitoring and reporting on the potential risks associated with salt fortification is important, particularly in high-risk populations and settings, and in contexts where multiple staple foods may be fortified with the same nutrient.

## Conclusions

Overall, iron-containing double and multiple fortified salts, namely DFS, TFS, QFS, and MMFS, improved hemoglobin concentration, DFS and MMFS reduced the prevalence of anemia and IDA, and MMFS reduced the odds of ID and improved folate status. These improvements were observed across adult and child population groups in many settings, but primarily in India. Limited efficacy trials exist for fortified salts other than DFS, and further evidence is needed to explore the effects of these fortified salts on nutrient status, as well as functional outcomes of child development, cognitive performance, work productivity, fatigue, and other outcomes related to iron and micronutrient status across children, adolescents, and adults.

## Author contributions

The authors’ responsibilities were as follows– LML, MBZ, WS: designed the research; GL, LML: conducted the analyses and reviewed the literature; LML: drafted the manuscript; All authors critically reviewed the manuscript; and all authors: read and approved the final manuscript.

## Funding

This research was supported by the Bill & Melinda Gates Foundation (INV-058682) through an award to the Iodine Global Network. The funder had no role in the study design; in the collection, analysis, and interpretation of data; in the writing of the report; and in the decision to submit the paper for publication.

## Conflict of interest

The authors report no conflicts of interest.
