# Supplementary material for: The Effects of Salt Fortified with Multiple Nutrients on Health Outcomes in Children, Adolescents, and Adults: A Systematic Review and Meta-Analysis
Source: Adv Nutr. 2025 Dec 4;17(1):100567. doi: 10.1016/j.advnut.2025.100567 (PMC12796936; doi:10.1016/j.advnut.2025.100567)

**Supplemental Materials for**

**The effects of salt fortified with multiple nutrients on health outcomes in children, adolescents and adults: a systematic review and meta-analysis**

Gitanjali Lall,^1^ Michael Zimmermann,^2^ Werner Schultink,^3^ Leila M Larson^1^

1. Department of Health Promotion, Education, and Behavior, University of South Carolina, USA
2. Radcliffe Department of Medicine, University of Oxford, UK
3. Iodine Global Network, Ontario, Canada

Supplemental Table 1: Pooled and single effect sizes and odds ratios stratified by study quality for fortified salt studies^1^

| **Outcome** | **Study quality** | | | |
| --- | --- | --- | --- | --- |
|  | **N** | **Strong or moderate** | **N** | **Weak** |
| **Double fortified salt (salt fortified with iron and iodine)** | | | | |
| Hemoglobin SMD | 9 | 0.47 (0.20, 0.74) | 17 | 0.30 (0.14, 0.46) |
| Hemoglobin MD (g/dL) | 9 | 0.54 (0.28, 0.80) | 17 | 0.44 (0.19, 0.69) |
| Serum ferritin SMD | 9 | 0.64 (-0.20, 1.49) | 2 | 0.36 (-1.50, 2.23) |
| Serum ferritin MD (μg/L) | 9 | 10.29 (4.88, 15.70) | 2 | 9.65 (7.78, 11.51) |
| ZnPP SMD | 4 | -0.62 (-3.16, 1.93) |  |  |
| sTfR SMD | 6 | -0.68 (-1.28, -0.09) |  |  |
| Body iron stores SMD | 4 | 0.69 (0.18, 1.20) |  |  |
| Serum folate SMD | 1 | -0.14 (-2.84, 2.56) |  |  |
| Serum B12 SMD | 1 | -0.36 (-31.18, 30.45) |  |  |
| Anemia OR | 5 | 0.35 (0.19, 0.66) | 9 | 0.48 (0.28, 0.83) |
| Iron deficiency anemia OR | 5 | 0.27 (0.17, 0.41) |  |  |
| **Salt fortified with folic acid and iodine** | | | | |
| Serum folate SMD | 1 | 0.11 (-4.77, 4.99) | 1 | 9.12 (8.21, 10.02) |
| Iron deficiency anemia OR |  |  |  |  |
| **Triple fortified salt (salt fortified with iron, vitamin A, and iodine)** | | | | |
| Hemoglobin SMD | 1 | 1.56 (1.42, 1.70) |  |  |
| Hemoglobin MD (g/dL) | 1 | 1.40 (1.26, 1.54) |  |  |
| ZnPP SMD | 1 | -0.87 (-5.35, 3.60) |  |  |
| sTfR SMD | 1 | -0.95 (-1.27, -0.64) |  |  |
| Body iron stores SMD | 1 | 1.35 (0.93, 1.77) |  |  |
| Serum retinol SMD | 1 | 1.98 (1.95, 2.00) |  |  |
| **Quadruple fortified salt (salt fortified with iron, folic acid, vitamin A, and iodine)** | | | | |
| Hemoglobin SMD | 1 | 0.33 (0.02, 0.63) |  |  |
| Hemoglobin MD (g/dL) | 1 | 0.54 (0.23, 0.85) |  |  |
| Serum ferritin SMD | 1 | 0.66 (-3.24, 4.57) |  |  |
| Serum ferritin MD (μg/L) | 1 | 14 (10.10, 17.90) |  |  |
| Serum folate SMD | 1 | 0.58 (-2.59, 3.76) |  |  |
| Serum B12 SMD | 1 | 0.16 (-32.73, 33.05) |  |  |
| **Multiple micronutrient fortified salt (salt fortified with iron, folic acid, vitamin A, iodine, and other nutrients)** | | | | |
| Hemoglobin SMD | 4 | 0.18 (-0.08, 0.45) | 2 | 0.32 (-0.06, 0.70) |
| Hemoglobin MD (g/dL) | 4 | 0.27 (-0.11, 0.64) | 2 | 0.41 (-0.21, 1.03) |
| Serum ferritin SMD | 4 | 0.23 (-0.32, 0.78) | 2 | 0.13 (-2.14, 2.41) |
| Ferritin MD (μg/L) | 4 | 3.88 (-1.92, 9.68) | 2 | 4.77 (-10.32, 19.87) |
| sTfR SMD | 4 | -0.16 (-0.63, 0.30) | 2 | -0.24 (-0.69, 0.21) |
| Body iron stores SMD | 4 | 0.23 (-0.24, 0.70) | 2 | 0.27 (-0.40, 0.94) |
| Serum folate SMD | 3 | 2.12 (1.38, 2.87) |  |  |
| Serum retinol SMD | 2 | 0.82 (0.27, 1.37) | 1 | -0.29 (-0.39, -0.19) |
| Serum B12 SMD | 2 | 2.13 (-2.08, 6.34) |  |  |
| Serum Zinc SMD | 3 | 0.07 (-0.75, 0.89) | 1 | 0.04(-3.58, 3.66) |
| Anemia OR | 4 | 0.79 (0.56, 1.11) | 1 | 0.38 (0.22, 0.68) |
| Iron deficiency anemia OR | 4 | 0.83 (0.64, 1.07) | 1 | 0.31 (0.14, 0.66) |
| Iron deficiency OR | 4 | 0.76 (0.55, 1.03) | 2 | 0.80 (0.30, 2.14) |

^1^MD, mean difference; OR, odds ratio; SMD, standardized mean difference; sTfR, serum transferrin receptor; ZnPP, zinc protoporphyrin. N refers to the number of comparisons.

Supplemental Table 2: Pooled and single effect sizes and odds ratios stratified by type of double fortified salt^1^

| **Outcome** | **Type of DFS** | | | | | | | | | | | |
| --- | --- | --- | --- | --- | --- | --- | --- | --- | --- | --- | --- | --- |
|  | **N** | **1a** | **N** | **1b** | **N** | **2** | **N** | **3** | **N** | **4** | **N** | **5** |
| **Double fortified salt (salt fortified with iron and iodine)** | | | | | | | | | | | | |
| Hemoglobin SMD | 3 | 0.21 (0.05, 0.36) | 4 | 0.22 (0.10, 0.33) | 10 | 0.21 (0.01, 0.40) | 2 | 0.34 (-0.06, 0.74) | 1 | 0.92 (0.79, 1.04) | 3 | 0.66 (-0.03, 1.35) |
| Hemoglobin MD (g/dL) | 3 | 0.27 (0.09, 0.45) | 4 | 0.25 (0.13, 0.38) | 10 | 0.30 (0.05, 0.55) | 2 | 0.69 (-0.34, 1.73) | 1 | 1.10 (1.04, 1.16) | 3 | 0.66 (0.04, 1.28) |
| Serum ferritin SMD |  |  | 4 | 0.50 (-0.58, 1.57) | 1 | -0.05 (- 4.14, 4.03) |  |  | 1 | 1.17 (-0.83, 3.18) | 3 | 0.59 (-0.87, 2.05) |
| Serum ferritin MD (μg/L) |  |  | 4 | 8.40 (6.51, 10.30) | 1 | -2.30 (-6.38, 1.78) |  |  | 1 | 23.00 (21.00, 25.00) | 3 | 11.42 (2.94, 19.90) |
| ZnPP SMD |  |  | 1 | -0.09 (-10.35, 10.17) |  |  |  |  | 1 | -0.63 (-4.20, 2.93) | 2 | -0.67 (-4.57, 3.22) |
| sTfR SMD |  |  | 2 | -0.97 (-2.39, 0.45) |  |  |  |  | 1 | -0.09 (-2.73, 2.54) | 3 | -0.53 (-1.12, 0.06) |
| Body iron stores SMD |  |  | 2 | 0.43 (0.12, 0.74) |  |  |  |  |  |  | 2 | 0.96 (0.01, 1.90) |
| Serum folate SMD |  |  |  |  |  |  |  |  |  |  |  |  |
| Serum B12 SMD |  |  |  |  |  |  |  |  |  |  |  |  |
| Anemia OR | 3 | 0.50 (0.47, 1.49) | 3 | 0.62 (0.35, 1.09) | 3 | 0.55 (0.20, 1.54) |  |  |  |  | 3 | 0.30 (0.10, 0.88) |
| Iron deficiency anemia OR |  |  | 1 | 0.22 (0.08, 0.62) |  |  |  |  | 1 | 0.20 (0.11, 0.38) | 3 | 0.32 (0.15, 0.65) |

^1^MD, mean difference; OR, odds ratio; SMD, standardized mean difference; sTfR, serum transferrin receptor; ZnPP, zinc protoporphyrin. N refers to the number of comparisons.

Supplemental Table 3: Pooled and single effect sizes and odds ratios stratified by study population for fortified salt studies^1^

| **Outcome** | **Population** | | | | | | | | | | | |
| --- | --- | --- | --- | --- | --- | --- | --- | --- | --- | --- | --- | --- |
|  | **N** | **Infants** | **N** | **School-age children and adolescents** | **N** | **Non-pregnant women** | **N** | **Pregnant women** | **N** | **Lactating women** | **N** | **Men** |
| **Double fortified salt (salt fortified with iron and iodine)** | | | | | | | | | | | | |
| Hemoglobin SMD | 2 | 0.08 (-0.26, 0.41) | 17 | 0.47 (0.25, 0.69) | 8 | 0.37 (0.12, 0.62) | 2 | 0.58 (0.21, 0.95) | 1 | 0.30 (-0.11, 0.72) | 4 | 0.36 (-0.03, 0.75) |
| Hemoglobin MD (g/dL) | 2 | 0.13 (-0.36, 0.62) | 17 | 0.61 (0.32, 0.90) | 8 | 0.53 (0.17, 0.90) | 2 | 0.69 (0.52, 0.86) | 1 | 0.50 (0.08, 0.92) | 4 | 0.53 (-0.04, 1.10) |
| Serum ferritin SMD |  |  | 8 | 0.62 (-0.17, 1.41) | 5 | 0.13(-2.07, 2.34) |  |  |  |  | 2 | 0.06 (3.03, 3.14) |
| Serum ferritin MD (μg/L) |  |  | 8 | 10.28 (4.59, 15.97) | 5 | 6.83(0.96, 12.70) |  |  |  |  | 2 | 3.99 (-8.45, 16.44) |
| ZnPP SMD |  |  | 4 | -0.62 (-3.16, 1.93) |  |  |  |  |  |  |  |  |
| sTfR SMD |  |  | 5 | -0.45 (-0.89, -0.01) | 1 | -1.68 (-1.71, -1.65) |  |  |  |  |  |  |
| Body iron stores SMD |  |  | 3 | 0.83 (0.22, 1.43) | 1 | 0.28 (-0.18, 0.74) |  |  |  |  |  |  |
| Serum folate SMD |  |  |  |  | 1 | -0.14 (-2.84, 2.56) |  |  |  |  |  |  |
| Serum B12 SMD |  |  |  |  | 1 | -0.36 (-31.18, 30.45) |  |  |  |  |  |  |
| Anemia OR | 1 | 0.16 (0.03, 0.80) | 11 | 0.34 (0.20, 0.57) | 4 | 0.43 (0.20, 0.94) | 1 | 0.28 (0.06, 1.40) |  |  | 1 | 0.22 (0.13, 0.38) |
| Iron deficiency anemia OR |  |  | 5 | 0.27 (0.17, 0.41) |  |  |  |  |  |  |  |  |
| **Salt fortified with folic acid and iodine** | | | | | | | | | | | | |
| Serum folate SMD |  |  |  |  | 2 | 4.94 (-3.86, 13.75) |  |  |  |  |  |  |
| Iron deficiency anemia OR |  |  |  |  | 1 | 0.13 (0.02, 1.02) |  |  |  |  |  |  |
| **Triple fortified salt (salt fortified with iron, vitamin A, and iodine)** | | | | | | | | | | | | |
| Hemoglobin SMD |  |  | 1 | 1.56 (1.42, 1.70) |  |  |  |  |  |  |  |  |
| Hemoglobin MD (g/dL) |  |  | 1 | 1.40 (1.26, 1.54) |  |  |  |  |  |  |  |  |
| ZnPP SMD |  |  | 1 | -0.87 (-5.35, 3.60) |  |  |  |  |  |  |  |  |
| sTfR SMD |  |  | 1 | -0.95 (-1.27, -0.64) |  |  |  |  |  |  |  |  |
| Body iron stores SMD |  |  | 1 | 1.35 (0.93, 1.77) |  |  |  |  |  |  |  |  |
| Serum retinol SMD |  |  | 1 | 1.98 (1.95, 2.00) |  |  |  |  |  |  |  |  |
| **Quadruple fortified salt (salt fortified with iron, folic acid, vitamin A, and iodine)** | | | | | | | | | | | | |
| Hemoglobin SMD |  |  |  |  | 1 | 0.33 (0.02, 0.63) |  |  |  |  |  |  |
| Hemoglobin MD (g/dL) |  |  |  |  | 1 | 0.54 (0.23, 0.85) |  |  |  |  |  |  |
| Serum ferritin SMD |  |  |  |  | 1 | 0.66(-3.24, 4.57) |  |  |  |  |  |  |
| Serum ferritin MD (μg/L) |  |  |  |  | 1 | 14.00 (10.10, 17.90) |  |  |  |  |  |  |
| Serum folate SMD |  |  |  |  | 1 | 0.58 (-2.59, 3.76) |  |  |  |  |  |  |
| Serum B12 SMD |  |  |  |  | 1 | 0.16 (-32.73, 33.05) |  |  |  |  |  |  |
| **Multiple micronutrient fortified salt (salt fortified with iron, folic acid, vitamin A, iodine, and other nutrients)** | | | | | | | | | | | | |
| Hemoglobin SMD |  |  | 4 | 0.36 (0.20, 0.53) | 3 | 0.14 (-0.23, 0.50) |  |  |  |  |  |  |
| Hemoglobin MD (g/dL) |  |  | 4 | 0.50 (0.22, 0.79) | 3 | 0.20 (-0.30, 0.71) |  |  |  |  |  |  |
| Serum ferritin SMD |  |  | 4 | 0.34 (-0.99, 1.67) | 3 | 0.22 (-0.40, 0.83) |  |  |  |  |  |  |
| Ferritin MD (μg/L) |  |  | 4 | 5.57 (-2.35, 13.48) | 3 | 4.96 (-2.44, 12.36) |  |  |  |  |  |  |
| sTfR SMD |  |  | 4 | -0.25 (-0.71, 0.22) | 3 | -0.20 (-0.60, 0.20) |  |  |  |  |  |  |
| Body iron stores SMD |  |  | 4 | 0.34 (-0.10, 0.78) | 3 | 0.25 (-0.22, 0.71) |  |  |  |  |  |  |
| Serum folate SMD |  |  | 1 | 0.94 (-0.26, 2.13) | 2 | 2.44 (2.14, 2.74) |  |  |  |  |  |  |
| Serum retinol SMD |  |  | 3 | 0.45 (-0.34, 1.24) |  |  |  |  |  |  |  |  |
| Serum B12 SMD |  |  |  |  | 2 | 2.13 (-2.08, 6.34) |  |  |  |  |  |  |
| Serum Zinc SMD |  |  | 2 | 0.07 (-3.37, 3.51) | 3 | 0.06 (-0.74, 0.86) |  |  |  |  |  |  |
| Anemia OR |  |  | 3 | 0.55 (0.42, 0.71) | 3 | 0.78 (0.41, 1.49) |  |  |  |  |  |  |
| Iron deficiency anemia OR |  |  | 3 | 0.61 (0.40, 0.92) | 3 | 0.72 (0.34, 1.51) |  |  |  |  |  |  |
| Iron deficiency OR |  |  | 4 | 0.74 (0.48, 1.14) | 3 | 0.72 (0.46, 1.15) |  |  |  |  |  |  |

^1^MD, mean difference; OR, odds ratio; SMD, standardized mean difference; sTfR, serum transferrin receptor; ZnPP, zinc protoporphyrin. N refers to the number of comparisons.

Supplemental Table 4: Pooled and single effect sizes and odds ratios stratified by average iron intake from fortified salt, intervention duration, baseline hemoglobin and anemia status, for fortified salt studies^1^

| **Outcome** | **Average iron intake from fortified salt** | | | | **Intervention duration** | | | | **Baseline hemoglobin concentration** | | | | **Baseline anemia prevalence** | | | |
| --- | --- | --- | --- | --- | --- | --- | --- | --- | --- | --- | --- | --- | --- | --- | --- | --- |
|  | **N** | **≤10 mg iron/person/day** | **N** | **>10 mg iron/person/day** | **N** | **<12m** | **N** | **>= 12m** | **N** | **<11 g/dL** | **N** | **≥11 g/dL** | **N** | **≤50%** | **N** | **>50%** |
| **Double fortified salt (salt fortified with iron and iodine)** | | | | | | | | | | | | | | | | |
| Hemoglobin SMD | 15 | 0.29 (0.15, 0.44) | 5 | 0.76(0.32, 1.20) | 14 | 0.43 (0.24, 0.63) | 6 | 0.45 (0.17, 0.72) | 10 | 0.24 (0.04, 0.45) | 15 | 0.39 (0.21, 0.57) | 6 | 0.28 (0.14, 0.43) | 9 | 0.41 (0.17, 0.66) |
| Hemoglobin MD (g/dL) | 15 | 0.44 (0.21, 0.66) | 5 | 0.89(0.36, 1.41) | 14 | 0.48 (0.28, 0.69) | 6 | 0.78 (0.32, 1.24) | 10 | 0.36 (0.04, 0.67) | 15 | 0.49 (0.29, 0.68) | 6 | 0.40 (0.10, 0.70) | 9 | 0.45 (0.21, 0.69) |
| Serum ferritin SMD | 4 | 0.59 (-0.43, 1.61) | 3 | 0.95 (-0.76, 2.65) | 9 | 0.63(-0.16, 1.43) | 2 | 0.06 (-3.03, 3.14) | 1 | 0.18 (-5.73, 6.10) | 10 | 0.60 (- 0.17, 1.38) | 3 | 0.56 (-0.36, 1.47) | 6 | 0.19 (-1.90, 2.27) |
| Serum ferritin MD (μg/L) | 4 | 7.84 (6.82, 8.86) | 3 | 15.44 ( 4.80, 26.07) | 9 | 11.44(7.01, 15.86) | 2 | 3.99 (-8.45, 16.44) | 1 | 6.8 (0.88, 12.72) | 10 | 10.27(5.49, 15.05) | 3 | 8.43 (7.22, 9.64) | 6 | 7.16 (0.97, 13.35) |
| ZnPP SMD | 2 | 0.10 (-6.30, 6.11) | 2 | -0.72 (-3.52, 2.07) | 4 | -0.62 (-3.16, 1.93) |  |  |  |  | 4 | -0.62 (-3.16, 1.93) | 2 | -0.10 (-6.30, 6.11) | 1 | -0.87 (-5.37, 3.64) |
| sTfR SMD | 3 | -0.22 (-0.52, 0.08) | 3 | -1.29 (-1.93, -0.65) | 6 | -0.68 (-1.28, -0.09) |  |  |  |  | 6 | -0.68 (-1.28, -0.09) | 2 | -0.20 (-0.54, 0.14) | 3 | -1.04 (-1.81, -0.26) |
| Body iron stores SMD | 2 | 0.52 (0.20, 0.84) | 2 | 0.86 (- 0.26, 1.99) | 4 | 0.69 (0.18, 1.20) |  |  |  |  | 4 | 0.69 (0.18, 1.20) | 2 | 0.52 (0.20, 0.84) | 2 | 0.86 (-0.26, 1.99) |
| Serum folate SMD |  |  |  |  | 1 | -0.14 (-2.84, 2.56) |  |  |  |  | 1 | -0.14 (-2.84, 2.56) |  |  |  |  |
| Serum B12 SMD |  |  |  |  | 1 | -0.36 (-31.18, 30.45) |  |  |  |  | 1 | -0.36 (-31.18, 30.45) |  |  |  |  |
| Anemia OR | 7 | 0.47 (0.32, 0.70) | 4 | 0.27 (0.13, 0.54) | 13 | 0.39 (0.26, 0.60) | 1 | 0.22(0.14, 0.34) | 5 | 0.42 (0.20, 0.89) | 9 | 0.45 (0.27, 0.74) | 5 | 0.59 (0.33, 1.07) | 8 | 0.40 (0.23, 0.69) |
| Iron deficiency anemia OR | 3 | 0.37 (0.22, 0.62) | 2 | 0.18 (0.11, 0.32) | 5 | 0.27 (0.17, 0.41) |  |  |  |  | 5 | 0.27 (0.17, 0.41) | 2 | 0.30 (0.15, 0.60) | 2 | 0.27 (0.07, 0.98) |
| **Salt fortified with folic acid and iodine** | | | | | | | | | | | | | | | | |
| Serum folate SMD |  |  |  |  | 2 | 4.94 (-3.86, 13.75) |  |  |  |  |  |  |  |  |  |  |
| Iron deficiency anemia OR |  |  |  |  | 1 | 0.13 (0.02, 1.02) |  |  |  |  |  |  |  |  |  |  |
| **Triple fortified salt (salt fortified with iron, vitamin A, and iodine)** | | | | | | | | | | | | | | | | |
| Hemoglobin SMD | 1 | 1.56 (1.42, 1.70) |  |  | 1 | 1.56 (1.42, 1.70) |  |  |  |  | 1 | 1.56 (1.42, 1.70) |  |  |  |  |
| Hemoglobin MD (g/dL) | 1 | 1.40 (1.26, 1.54) |  |  | 1 | 1.40 (1.26, 1.54) |  |  |  |  | 1 | 1.40 (1.26, 1.54) |  |  |  |  |
| ZnPP SMD | 1 | -0.87 (-5.35, 3.60 |  |  | 1 | -0.87 (-5.35, 3.60) |  |  |  |  | 1 | -0.87 (-5.35, 3.60) |  |  |  |  |
| sTfR SMD | 1 | -0.95 (-1.27, -0.64) |  |  | 1 | -0.95 (-1.27, -0.64) |  |  |  |  | 1 | -0.95 (-1.27, -0.64) |  |  |  |  |
| Body iron stores SMD | 1 | 1.35 (0.93, 1.77) |  |  | 1 | 1.35 (0.93, 1.77) |  |  |  |  | 1 | 1.35 (0.93, 1.77) |  |  |  |  |
| Serum retinol SMD | 1 | 1.98 (1.95, 2.00) |  |  | 1 | 1.98 (1.95, 2.00) |  |  |  |  | 1 | 1.98 (1.95, 2.00) |  |  |  |  |
| **Quadruple fortified salt (salt fortified with iron, folic acid, vitamin A, and iodine)** | | | | | | | | | | | | | | | | |
| Hemoglobin SMD |  |  |  |  | 1 | 0.33 (0.02, 0.63) |  |  |  |  | 1 | 0.33 (0.02, 0.63) |  |  |  |  |
| Hemoglobin MD (g/dL) |  |  |  |  | 1 | 0.54 (0.23, 0.85) |  |  |  |  | 1 | 0.54 (0.23, 0.85) |  |  |  |  |
| Serum ferritin SMD |  |  |  |  | 1 | 0.66 (-3.24, 4.57) |  |  |  |  | 1 | 0.66 (-3.24, 4.57) |  |  |  |  |
| Serum ferritin MD (μg/L) |  |  |  |  | 1 | 14.00(10.10, 17.90) |  |  |  |  | 1 | 14.00 (10.10, 17.90) |  |  |  |  |
| Serum folate SMD |  |  |  |  | 1 | 0.58 (-2.59, 3.76) |  |  |  |  | 1 | 0.58 (-2.59, 3.76) |  |  |  |  |
| Serum B12 SMD |  |  |  |  | 1 | 0.16 (-32.73, 33.05) |  |  |  |  | 1 | 0.16 (-32.73, 33.05) |  |  |  |  |
| **Multiple micronutrient fortified salt (salt fortified with iron, folic acid, vitamin A, iodine, and other nutrients)** | | | | | | | | | | | | | | | | |
| Hemoglobin SMD | 5 | 0.19 (-0.04, 0.42) | 1 | 0.41 (0.24, 0.57) | 3 | 0.44 (0.36, 0.52) | 3 | 0.01 (-0.11, 0.13) | 1 | 0.13 (0.00, 0.26) | 5 | 0.25 (0, 0.49) | 2 | -0.04 (-0.13, 0.04) | 3 | 0.44 (0.36, 0.52) |
| Hemoglobin MD (g/dL) | 5 | 0.24 (-0.07, 0.54) | 1 | 0.70 (0.54, 0.86) | 3 | 0.63 (0.47, 0.78) | 3 | 0.00 (-0.11, 0.11) | 1 | 0.10 (-0.03, 0.23) | 5 | 0.36 (0.02, 0.70) | 2 | -0.05 (-0.15, 0.05) | 3 | 0.63 (0.47, 0.78) |
| Serum ferritin SMD | 5 | 0.20 (-0.36, 0.75) | 3 | 0.61 (-1.30, 2.52) | 3 | 0.46 (-1.03, 1.94) | 3 | 0.20 (-0.41, 0.80) |  |  | 5 | 0.23 (-0.30, 0.76) | 2 | 0.21 (-0.44, 0.86) | 3 | 0.46 (-1.03, 1.94) |
| Ferritin MD (μg/L) | 5 | 2.46 (-2.63, 7.56) | 1 | 12.40 (10.49, 14.31) | 3 | 8.39 (0.41, 16.38) | 3 | 0.21 (-3.32, 3.73) |  |  | 5 | 5.53 (-0.07, 11.13) | 2 | 1.45 (-2.38, 5.27) | 3 | 8.39 (0.41, 16.38) |
| sTfR SMD | 5 | -0.08 (-0.37, 0.21) | 1 | -0.75 (-1.05, -0.44) | 3 | -0.32 (-0.95, 0.32) |  | -0.08(-0.42, 0.26) |  |  | 5 | -0.22 (-0.61, 0.17) | 2 | -0.10 (-0.62, 0.42) | 3 | -0.32 (-0.95, 0.32) |
| Body iron stores SMD | 5 | 0.11 (-0.19, 0.41) | 1 | 0.77 (0.53, 1.01) | 3 | 0.47 (-0.02, 0.96) | 3 | -0.04 (-0.39, 0.30) |  |  | 5 | 0.32 (-0.07, 0.7) | 2 | -0.02 (-0.49, 0.46) | 3 | 0.47 (-0.02, 0.96) |
| Serum folate SMD | 3 | 2.12 (1.38, 2.87) |  |  | 1 | 0.94 (-0.26, 2.13) | 2 | 2.44 (2.14, 2.74) |  |  | 3 | 2.12 (1.38, 2.87) | 2 | 2.44 (2.14, 2.74) | 1 | 0.94 (-0.26, 2.13) |
| Serum retinol SMD | 2 | 0.41 (0.95, 1.77) | 1 | 0.54 (0.50, 0.58) | 2 | 0.82 (0.27, 1.37) | 1 | -0.29( -0.39, -0.19) | 1 | -0.29 (-0.39, -0.19) | 2 | 0.82 (0.27, 1.37) |  |  | 2 | 0.82 (0.27, 1.37) |
| Serum B12 SMD | 2 | 2.13 (-2.08, 6.34) |  |  |  |  | 2 | 2.13 (-2.08, 6.34) |  |  | 2 | 2.13 (-2.08, 6.34) | 2 | 2.13 (-2.08, 6.34) |  |  |
| Serum Zinc SMD | 4 | 0.06(-0.74, 0.86) |  |  | 2 | 0.07(-3.37, 3.51) | 2 | 0.06 (-0.76. 0.89) |  |  | 4 | 0.06(-0.74, 0.86) | 2 | 0.06 (-0.76, 0.89) | 2 | 0.07(-3.37, 3.51) |
| Anemia OR | 4 | 0.74 (0.47, 1.17) | 1 | 0.56 (0.38, 0.82) | 5 | 0.70 (0.49, 1.00) | 2 | 1.08 (0.77, 1.52) |  |  | 5 | 0.70 (0.49, 1) | 2 | 1.08 (0.77, 1.52) | 3 | 0.55 (0.42, 0.71) |
| Iron deficiency anemia OR | 4 | 0.74 (0.46, 1.19) | 1 | 0.69 (0.47, 1.03) | 3 | 0.61 (0.40, 0.92) | 2 | 1.02 (0.63, 1.65) |  |  | 5 | 0.74 (0.52, 1.05) | 2 | 1.02 (0.63, 1.65) | 3 | 0.61 (0.40, 0.92) |
| Iron deficiency OR | 5 | 0.85 (0.61, 1.18) | 1 | 0.56 (0.38, 0.82) | 3 | 0.61 (0.44, 0.84) | 3 | 0.98 (0.66, 1.45) |  |  | 5 | 0.70 (0.53, 0.94) | 2 | 0.86 (0.53, 1.40) | 3 | 0.61(0.44, 0.84) |

^1^MD, mean difference; OR, odds ratio; SMD, standardized mean difference; sTfR, serum transferrin receptor; ZnPP, zinc protoporphyrin. N refers to the number of comparisons.

Supplemental Table 5: Pooled and single effect sizes and odds ratios stratified by study design (effectiveness vs efficacy), for fortified salt studies^1,2^

| **Outcome** | **Study type** | | | |
| --- | --- | --- | --- | --- |
|  | **N** | **Effectiveness** | **N** | **Efficacy** |
| **Double fortified salt (salt fortified with iron and iodine)** | | | | |
| Hemoglobin SMD | - | - | 26 | 0.30 (0.14, 0.46) |
| Hemoglobin MD (g/dL) | - | - | 26 | 0.47 (0.03, 0.91) |
| Serum ferritin SMD | - | - | 11 | 0.60 (-0.17, 1.37) |
| Serum ferritin MD (μg/L) | - | - | 11 | 9.98(5.59, 14.36) |
| ZnPP SMD | - | - | 4 | -0.62 (-3.16, 1.93) |
| sTfR SMD | - | - | 6 | -0.68 (-1.28, -0.09) |
| Body iron stores SMD | - | - | 4 | 0.69 (0.18, 1.20) |
| Serum folate SMD | - | - | 1 | -0.14 (-2.84, 2.56) |
| Serum B12 SMD | - | - | 1 | -0.36 (-31.18, 30.45) |
| Anemia OR | - | - | 14 | 0.43 (0.28, 0.64) |
| Iron deficiency anemia OR | - | - | 5 | 0.27 (0.17, 0.41) |
| **Triple fortified salt (salt fortified with iron, vitamin A, and iodine)** | | | | |
| Hemoglobin SMD | - | - | 1 | 1.56 (1.42, 1.70) |
| Hemoglobin MD (g/dL) | - | - | 1 | 1.40 (1.26, 1.54) |
| ZnPP SMD | - | - | 1 | -0.87 (-5.35, 3.60) |
| sTfR SMD | - | - | 1 | -0.95 (-1.27, -0.64) |
| Body iron stores SMD | - | - | 1 | 1.35 (0.93, 1.77) |
| Serum retinol SMD | - | - | 1 | 1.98 (1.95, 2.00) |
| **Quadruple fortified salt (salt fortified with iron, folic acid, vitamin A, and iodine)** | | | | |
| Hemoglobin SMD | - | - | 1 | 0.33 (0.02, 0.63) |
| Hemoglobin MD (g/dL) | - | - | 1 | 0.54 (0.23, 0.85) |
| Serum ferritin SMD | - | - | 1 | 0.66 (-3.24, 4.57) |
| Serum ferritin MD (μg/L) | - | - | 1 | 14 (10.10, 17.90) |
| Serum folate SMD | - | - | 1 | 0.58 (-2.59, 3.76) |
| Serum B12 SMD | - | - | 1 | 0.16 (-32.73, 33.05) |
| **Multiple micronutrient fortified salt (salt fortified with iron, folic acid, vitamin A, iodine, and other nutrients)** | | | | |
| Hemoglobin SMD | 2 | 0.26 (0, 0.53) | 4 | 0.21 (-0.09, 0.50) |
| Hemoglobin MD (g/dL) | 2 | 0.10 (-0.03, 0.23) | 4 | 0.47 (0.03, 0.91) |
| Serum ferritin SMD | 2 | 0.40 (-1.21, 2.01) | 4 | 0.21(-0.37, 0.79) |
| Ferritin MD (μg/L) | 2 | 4.80 (-10.20, 19.79) | 4 | 3.77 (1.91, 9.45) |
| sTfR SMD | 2 | -0.40 (-1.10, 0.31) | 4 | -0.09(-0.46, 0.28) |
| Body iron stores SMD | 2 | 0.38(-0.45, 1.20) | 4 | 0.16 (-0.21, 0.53) |
| Serum folate SMD | - | - | 3 | 2.12 (1.38, 2.87) |
| Serum retinol SMD | 2 | 0.13(-0.68, 0.93) | 1 | 1.10 (1.08, 1.12) |
| Serum B12 SMD | - | - | 2 | 2.13 (-2.08, 6.34) |
| Serum Zinc SMD | - | - | 4 | 0.06 (-0.74, 0.86) |
| Anemia OR | 1 | 0.56 (0.38, 0.82) | 2 | 0.74 (0.47, 1.17) |
| Iron deficiency anemia OR | 1 | 0.69 (0.47, 1.03) | 4 | 0.74 (0.46, 1.19) |
| Iron deficiency OR | 2 | 0.84 (0.36, 1.94) | 4 | 0.76 (0.54, 1.07) |

^1^Efficacy studies were defined as randomized or unspecified efficacy trials. Effectiveness studies were defined as randomized effectiveness and quasi-experimental effectiveness trials. One group pre-post-test studies were excluded from this stratified analysis. All FISFA studies were One group pre-post-test studies

^2^MD, mean difference; OR, odds ratio; SMD, standardized mean difference; sTfR, serum transferrin receptor; ZnPP, zinc protoporphyrin. N refers to the number of comparisons.

Supplemental Figure 1: Funnel plot for hemoglobin concentration standardized mean difference among studies of double fortified salt


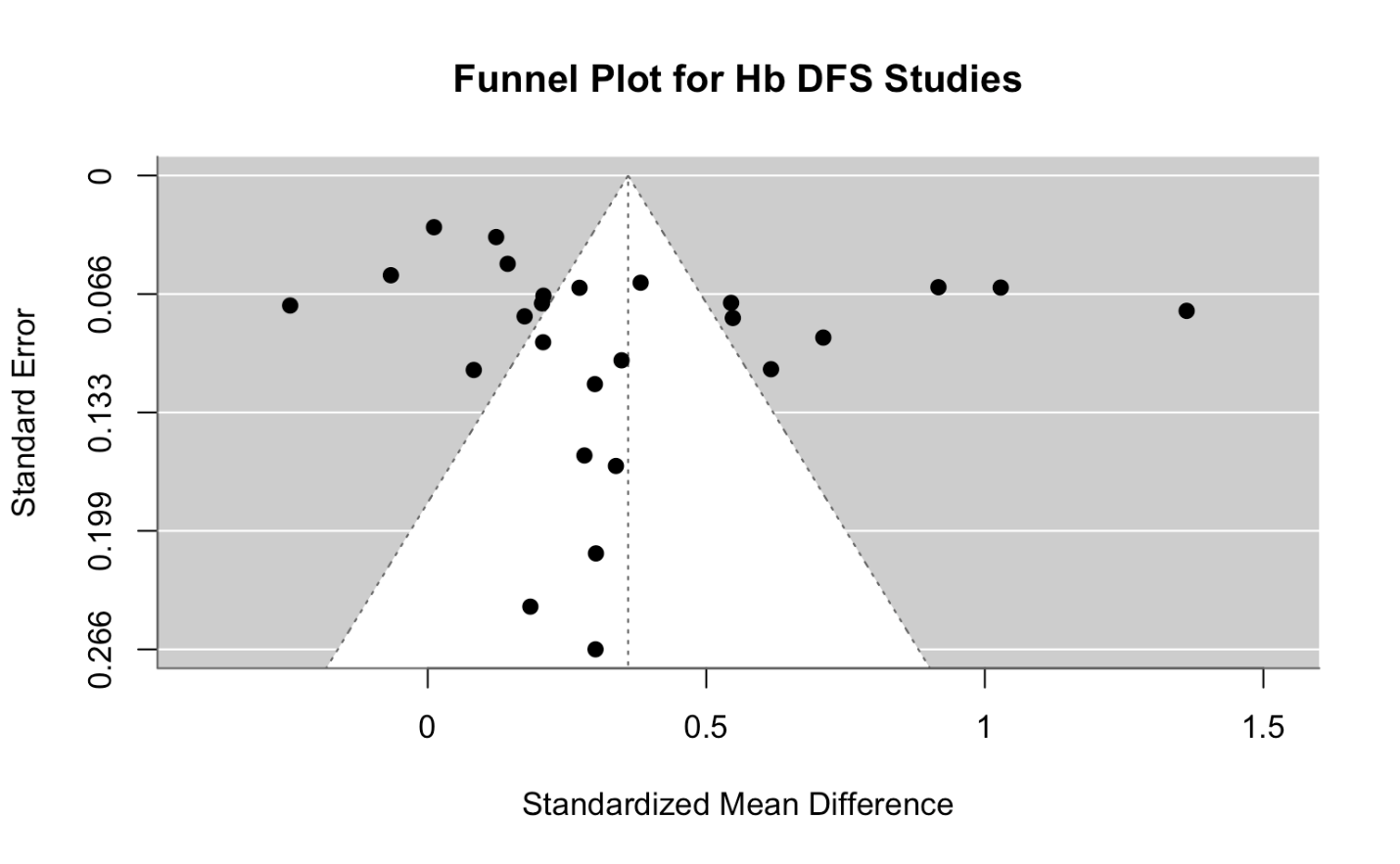


Supplemental Figure 2: Funnel plot for hemoglobin concentration mean difference among studies of double fortified salt


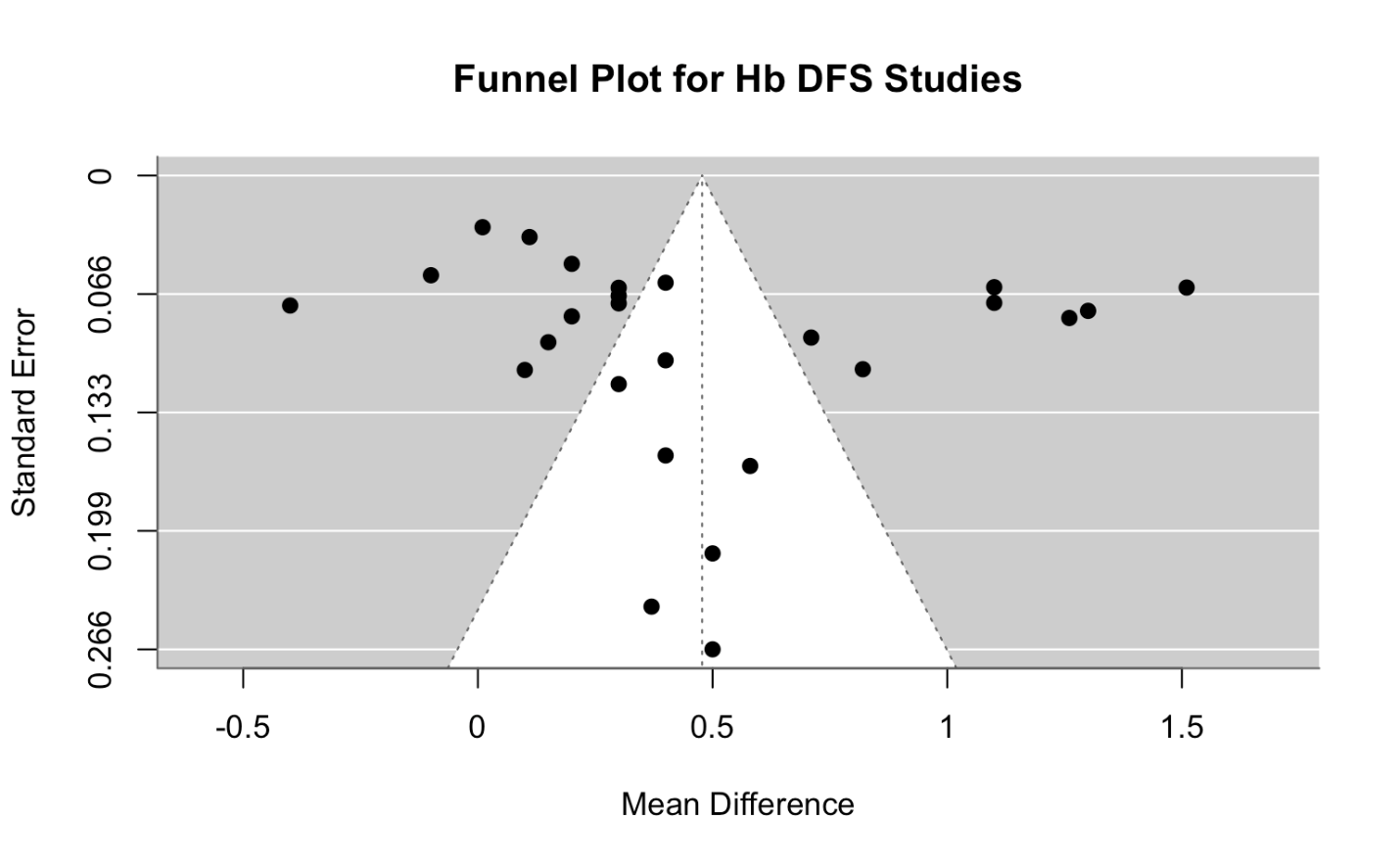


Supplemental Figure 3: Funnel plot for ferritin concentration standardized mean difference among studies of double fortified salt


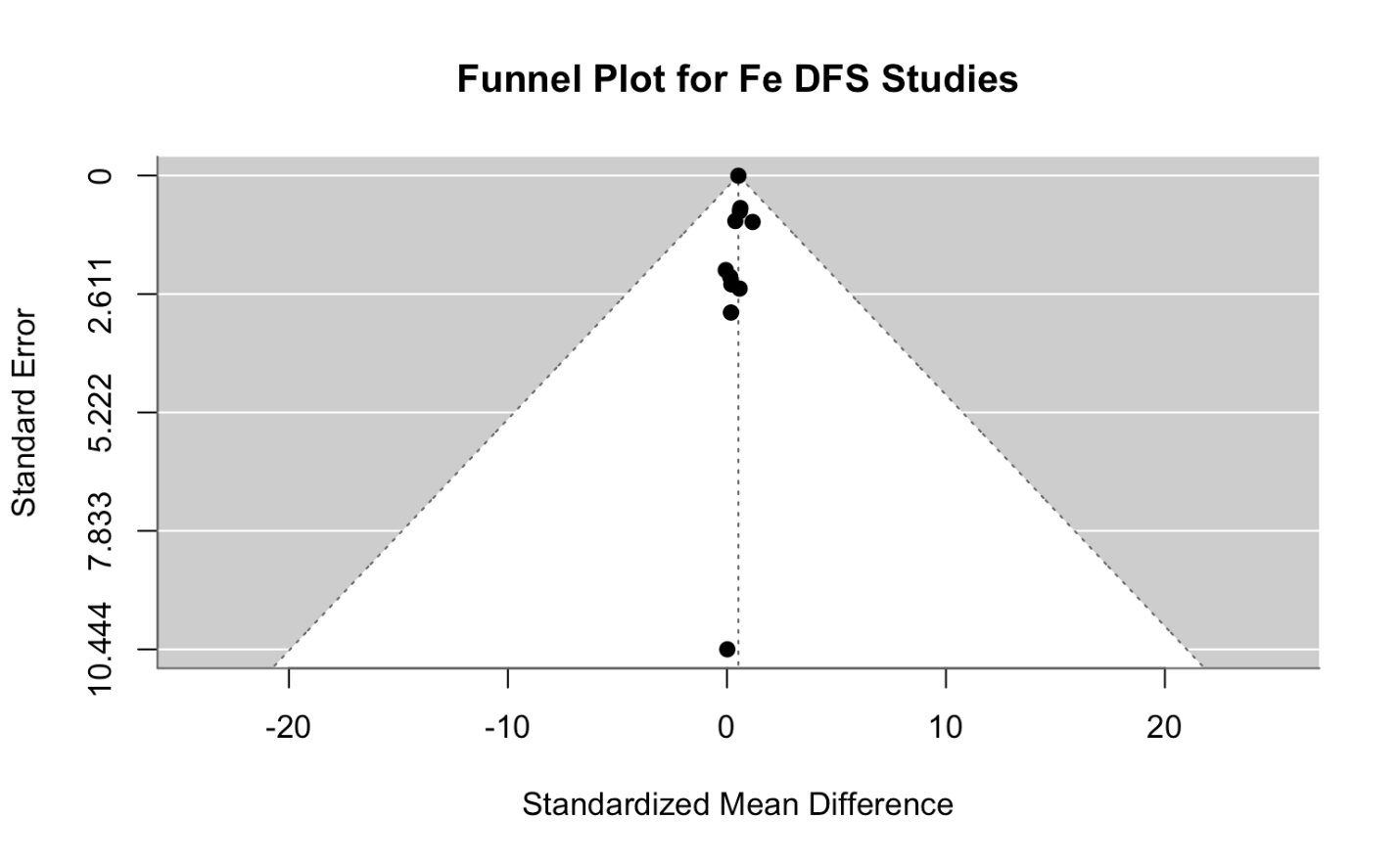


Supplemental Figure 4: Funnel plot for ferritin concentration mean difference among studies of double fortified salt


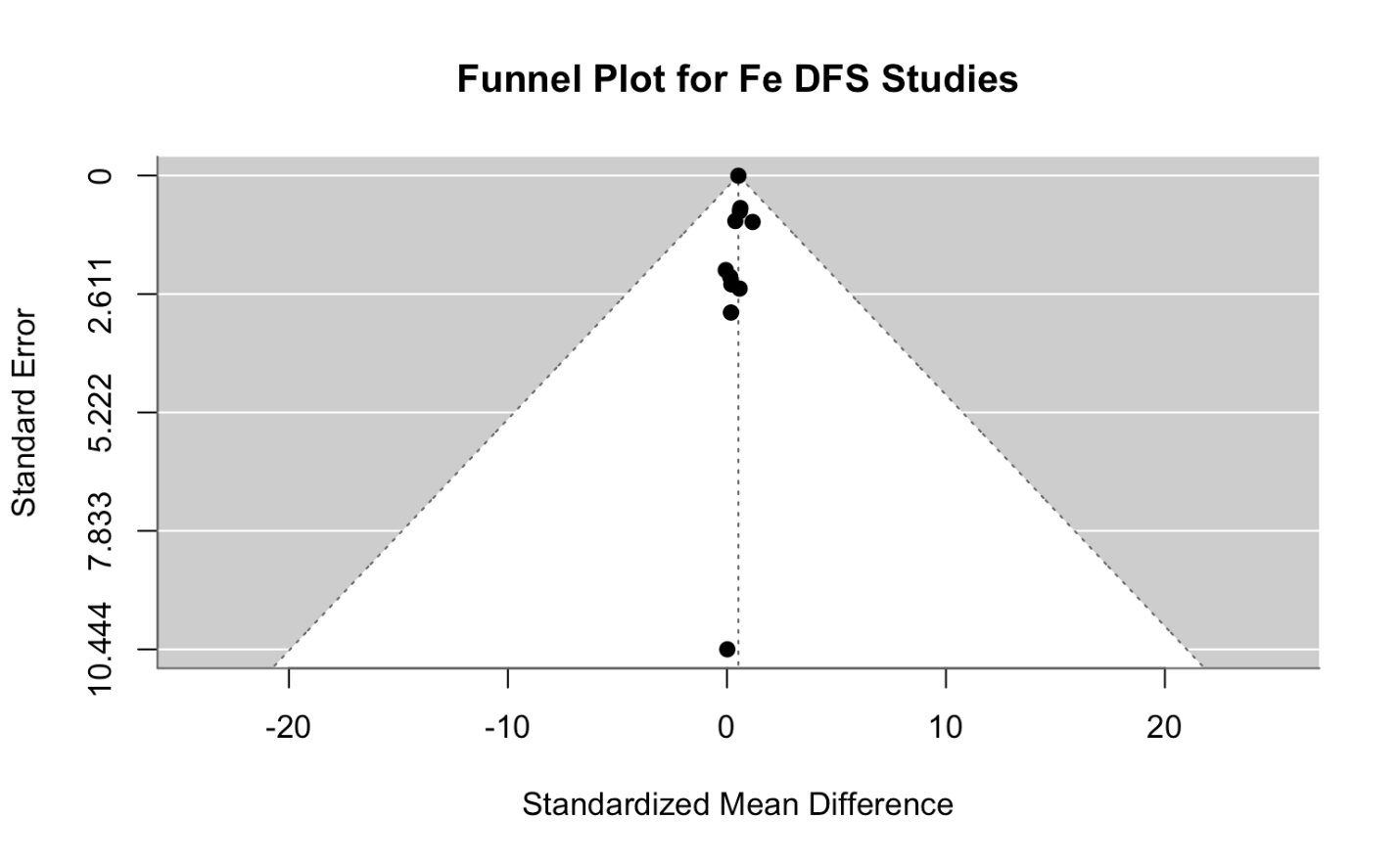


Supplemental Figure 5: Funnel plot for zinc protoporphyrin concentration standardized mean difference among studies of double fortified salt


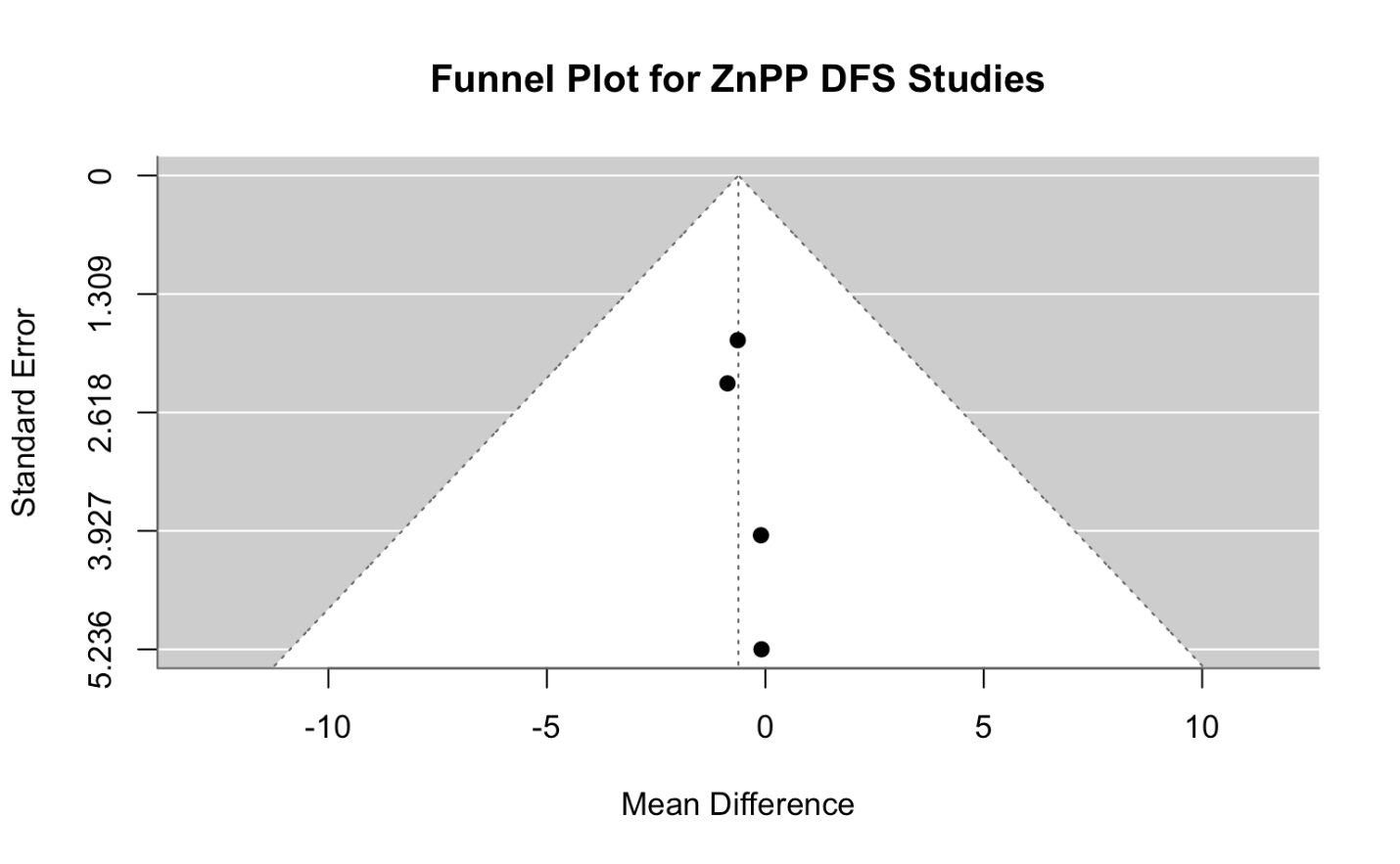


Supplemental Figure 6: Funnel plot for serum transferrin receptor concentration standardized mean difference among studies of double fortified salt


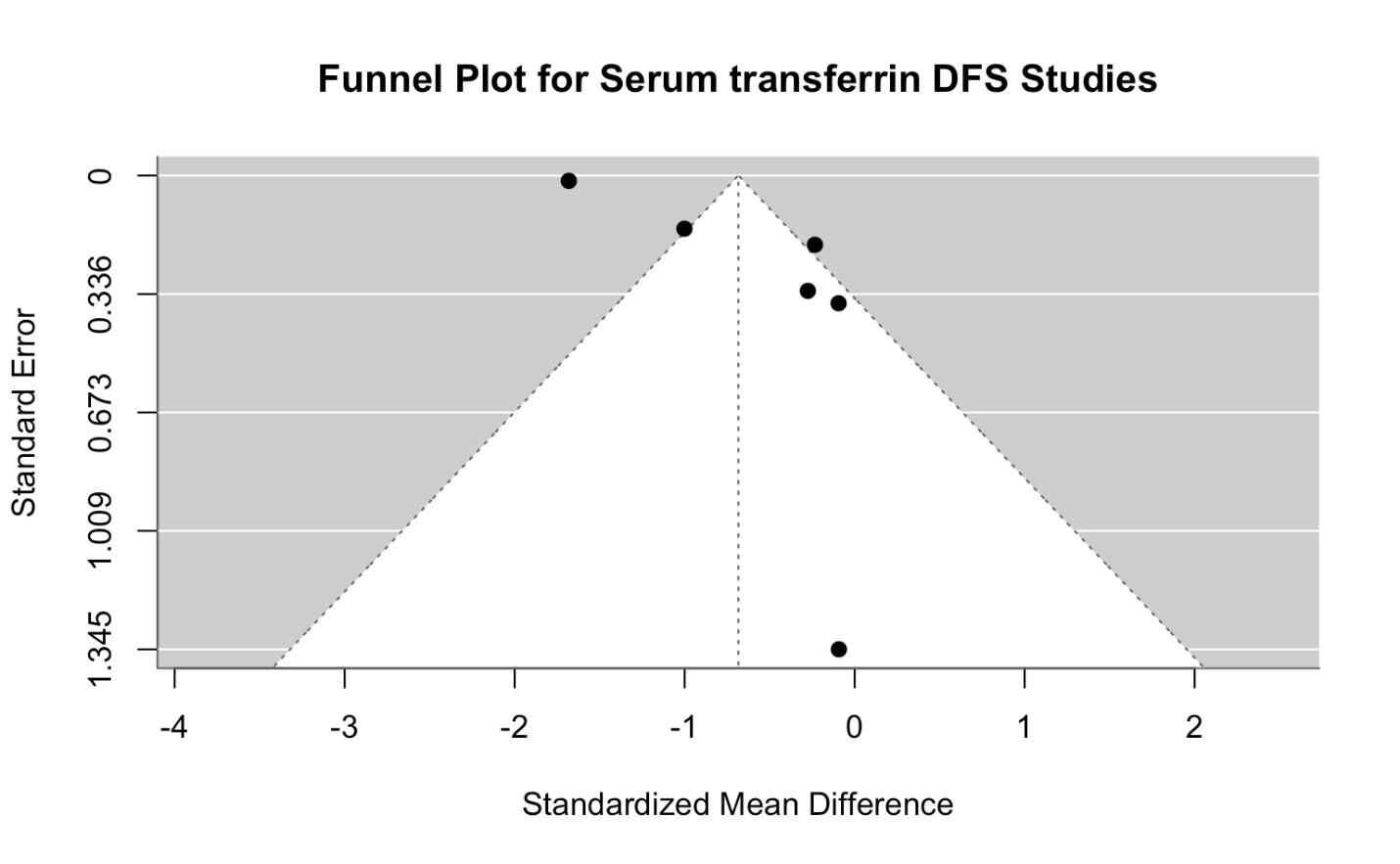


Supplemental Figure 7: Funnel plot for body iron stores standardized mean difference among studies of double fortified salt


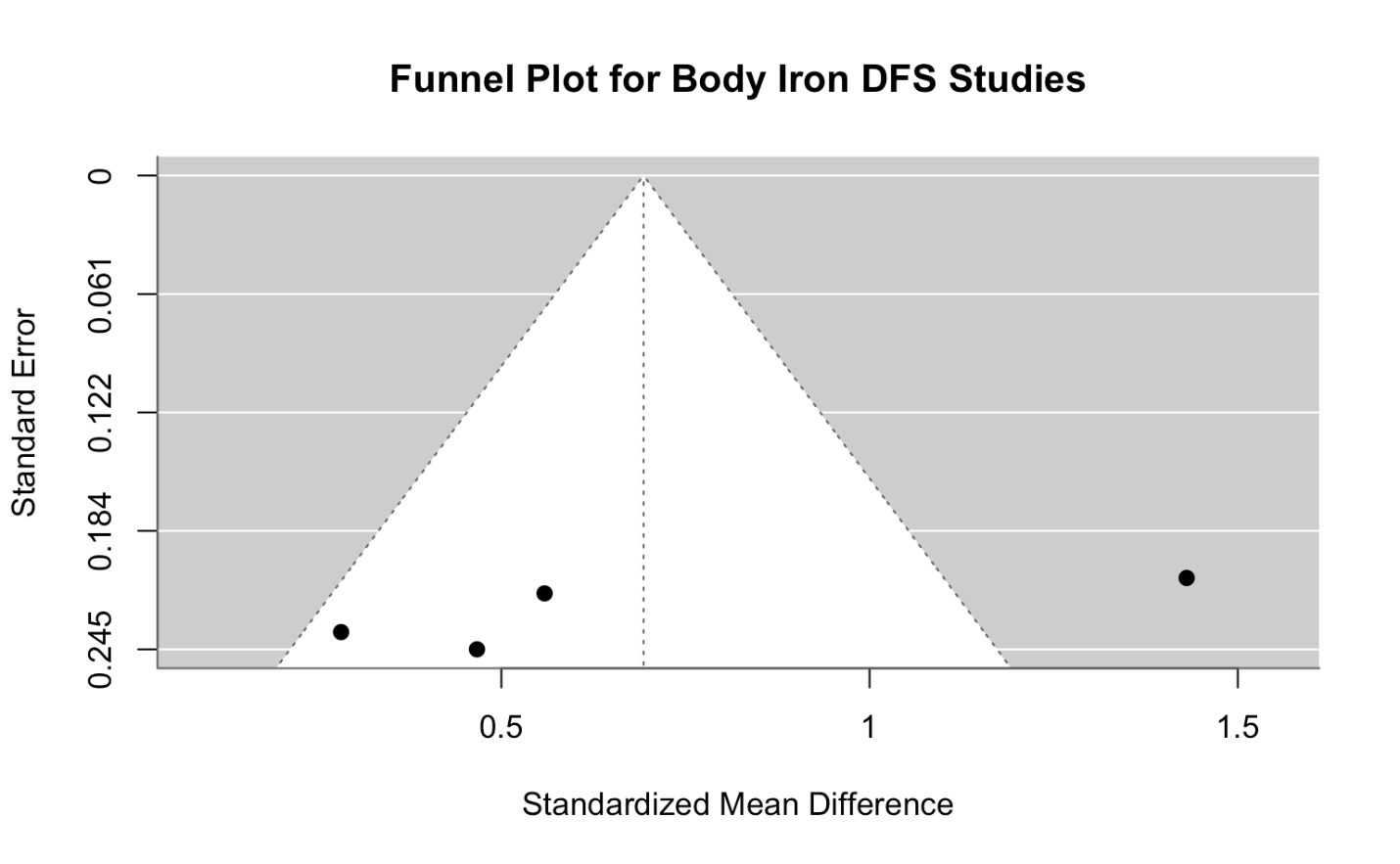


Supplemental Figure 8: Funnel plot for anemia odds ratio among studies of double fortified salt


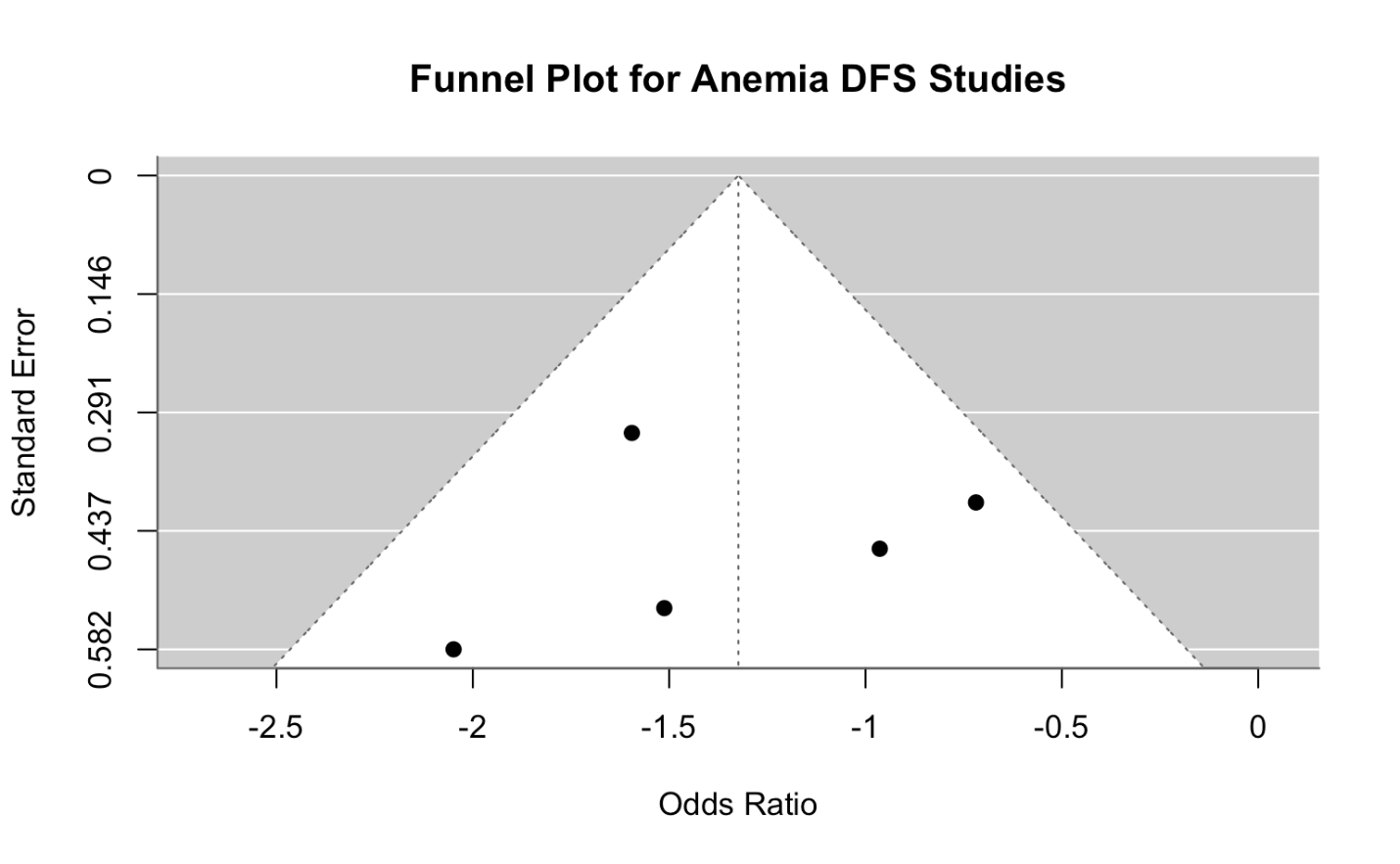


Supplemental Figure 9: Funnel plot for iron deficiency anemia odds ratio among studies of double fortified salt


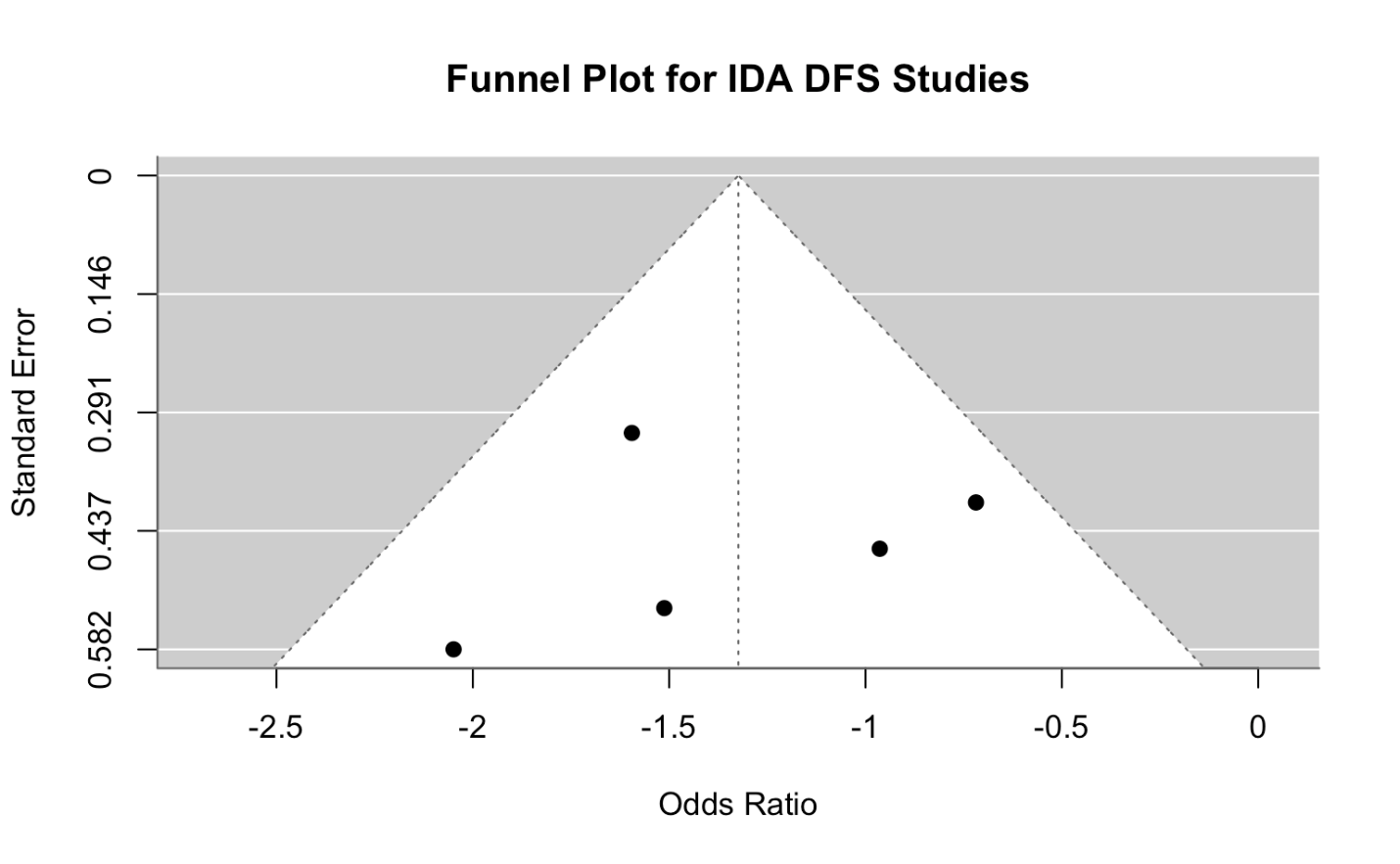


Supplemental Figure 10: Funnel plot for folate concentration standardized mean difference among studies of salt fortified with folic acid and iodine


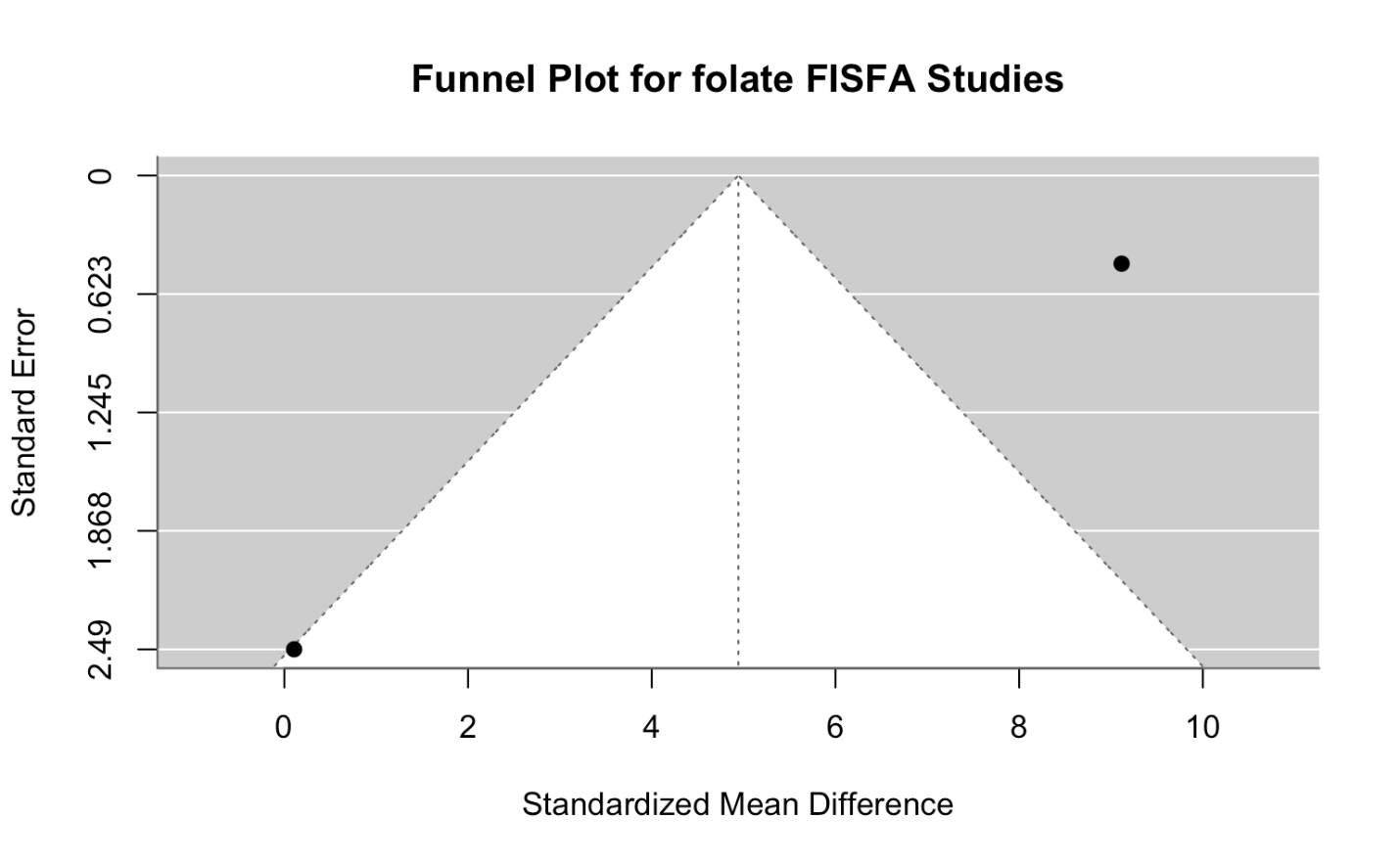


Supplemental Figure 11: Funnel plot for hemoglobin concentration standardized mean difference among studies of multiple micronutrient fortified salt


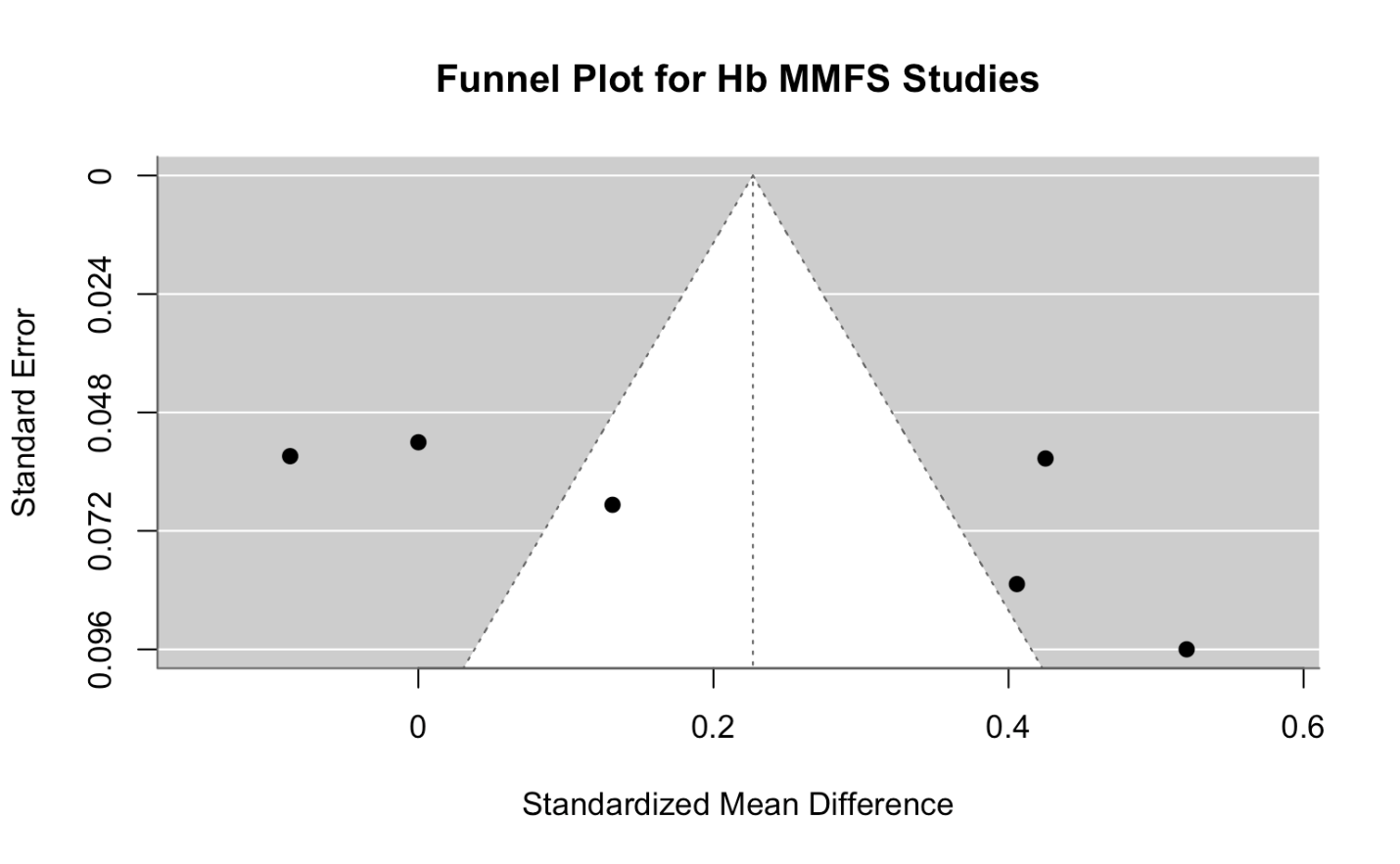


Supplemental Figure 12: Funnel plot for hemoglobin concentration mean difference among studies of multiple micronutrient fortified salt


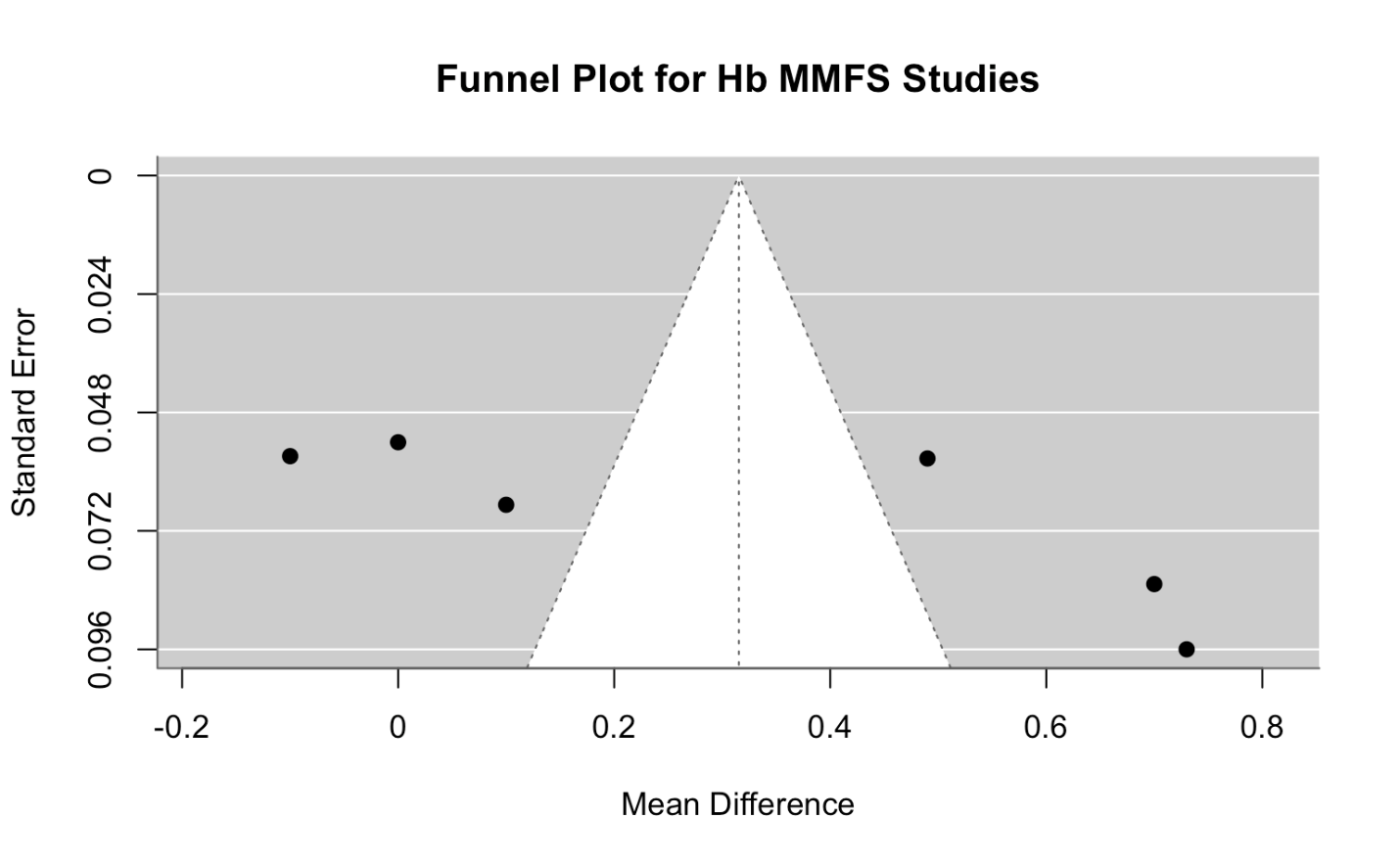


Supplemental Figure 13: Funnel plot for ferritin concentration standardized mean difference among studies of multiple micronutrient fortified salt


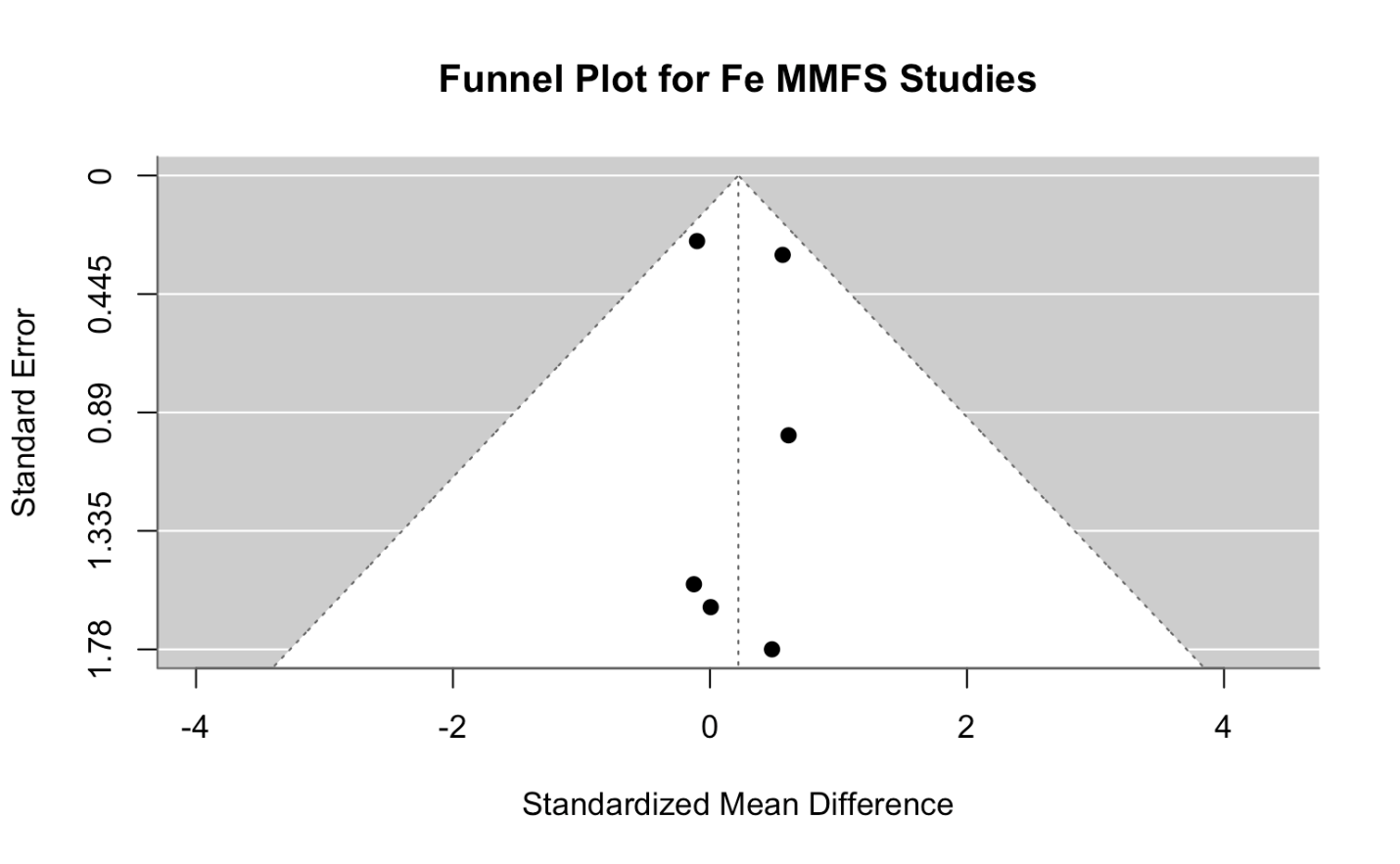


Supplemental Figure 14: Funnel plot for ferritin concentration mean difference among studies of multiple micronutrient fortified salt


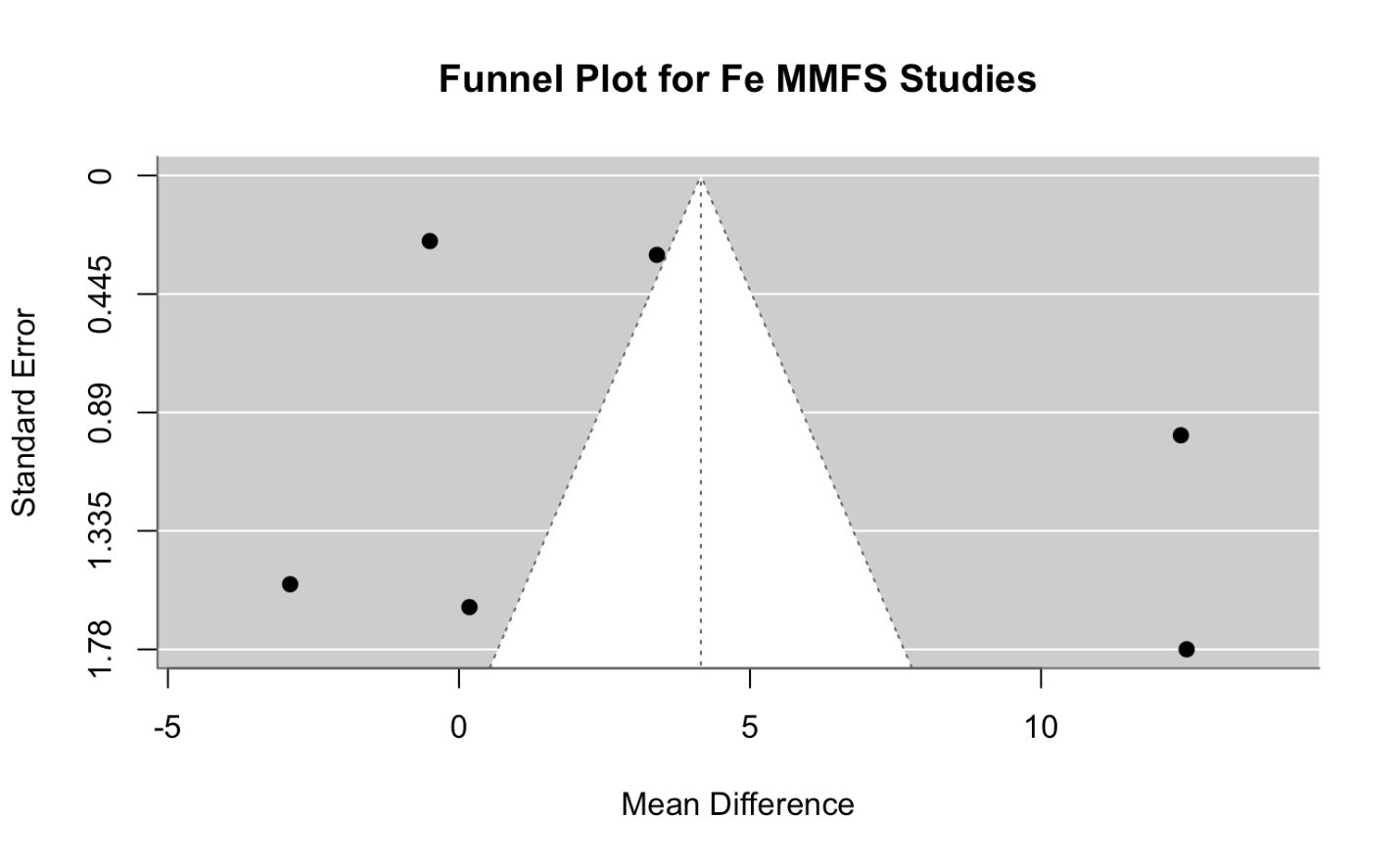


Supplemental Figure 15: Funnel plot for serum transferrin receptor concentration standardized mean difference among studies of multiple micronutrient fortified salt


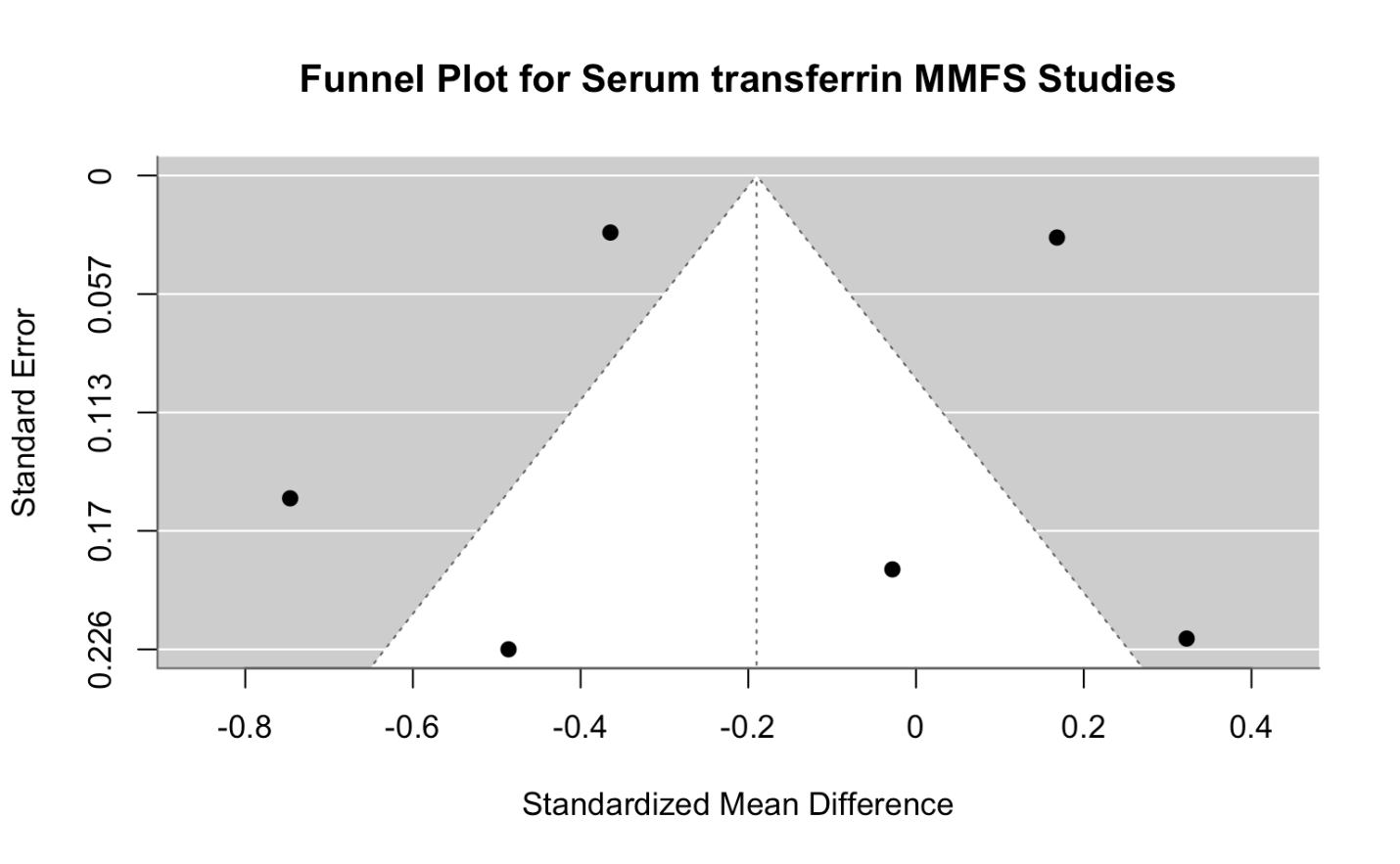


Supplemental Figure 16: Funnel plot for body iron stores standardized mean difference among studies of multiple micronutrient fortified salt


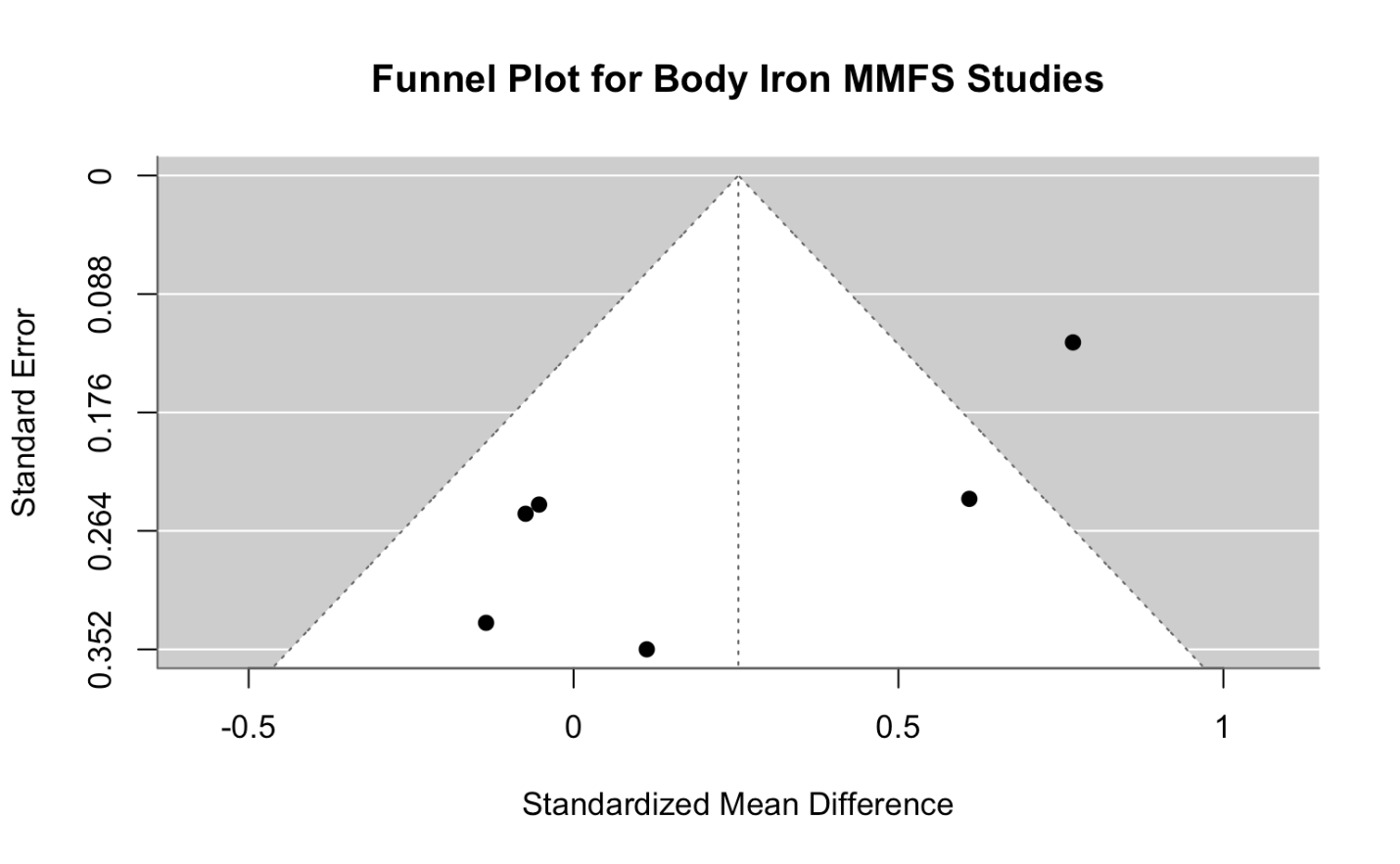


Supplemental Figure 17: Funnel plot for serum folate concentration standardized mean difference among studies of multiple micronutrient fortified salt


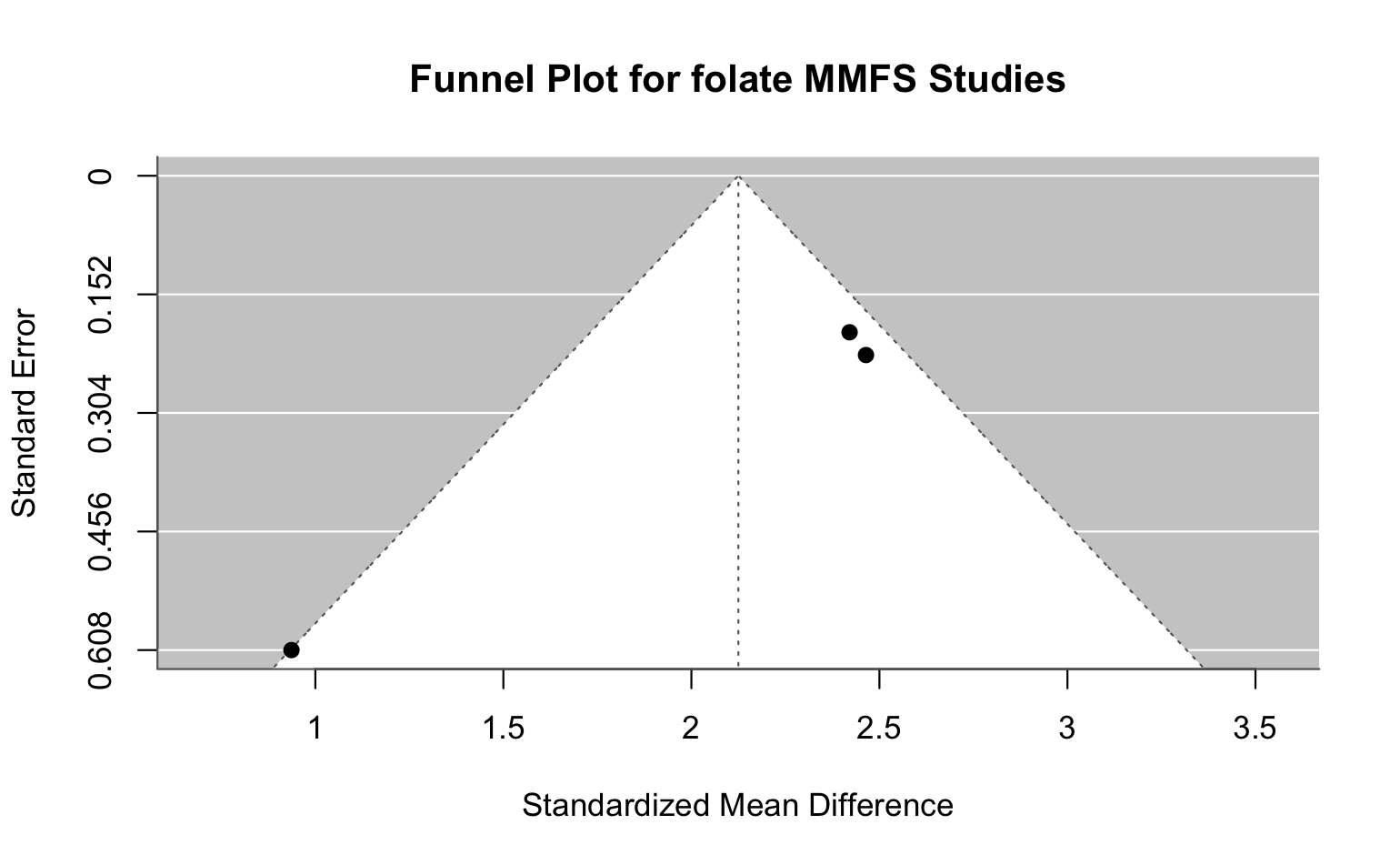


Supplemental Figure 18: Funnel plot for serum retinol concentration standardized mean difference among studies of multiple micronutrient fortified salt


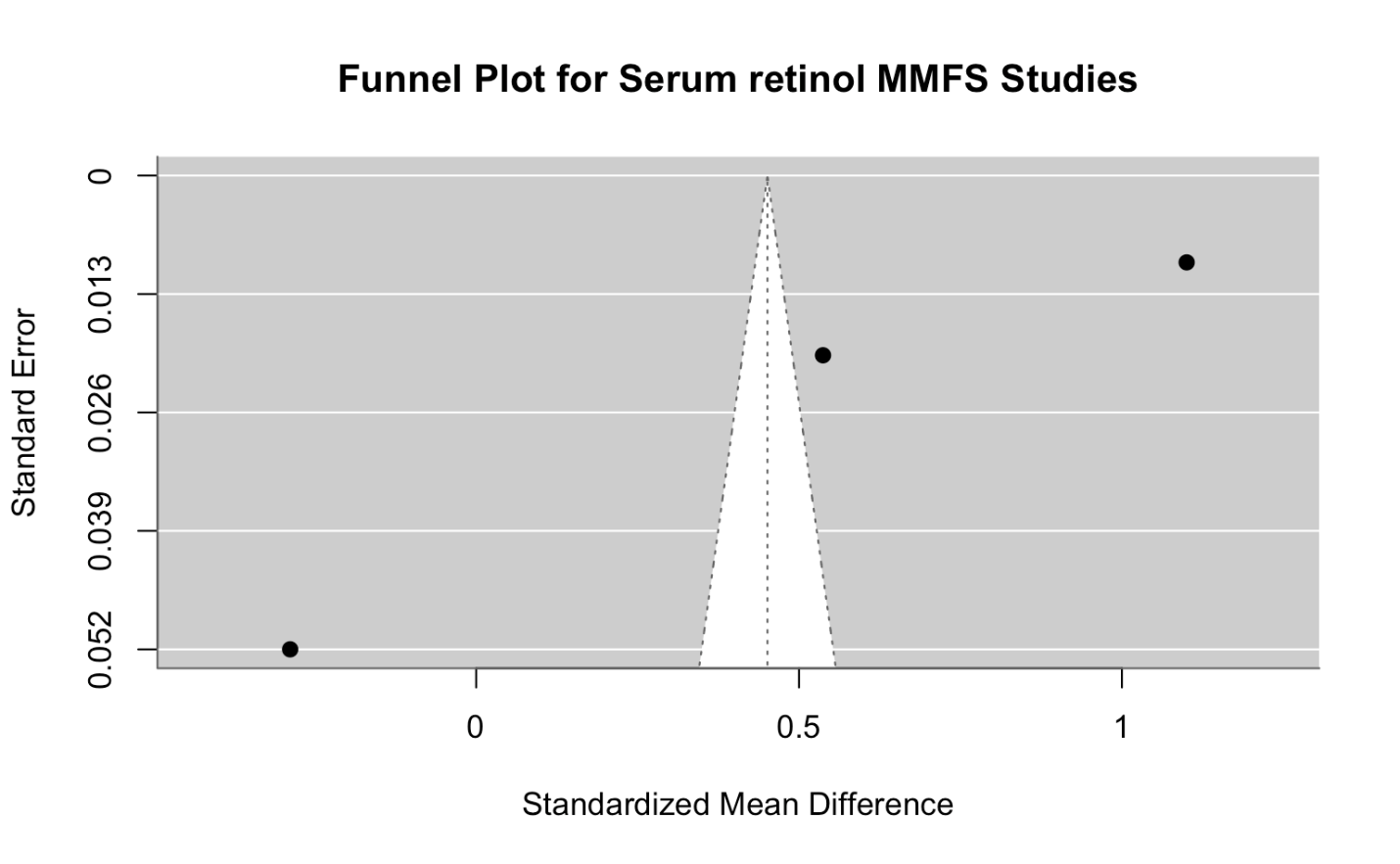


Supplemental Figure 19: Funnel plot for serum vitamin B12 concentration standardized mean difference among studies of multiple micronutrient fortified salt


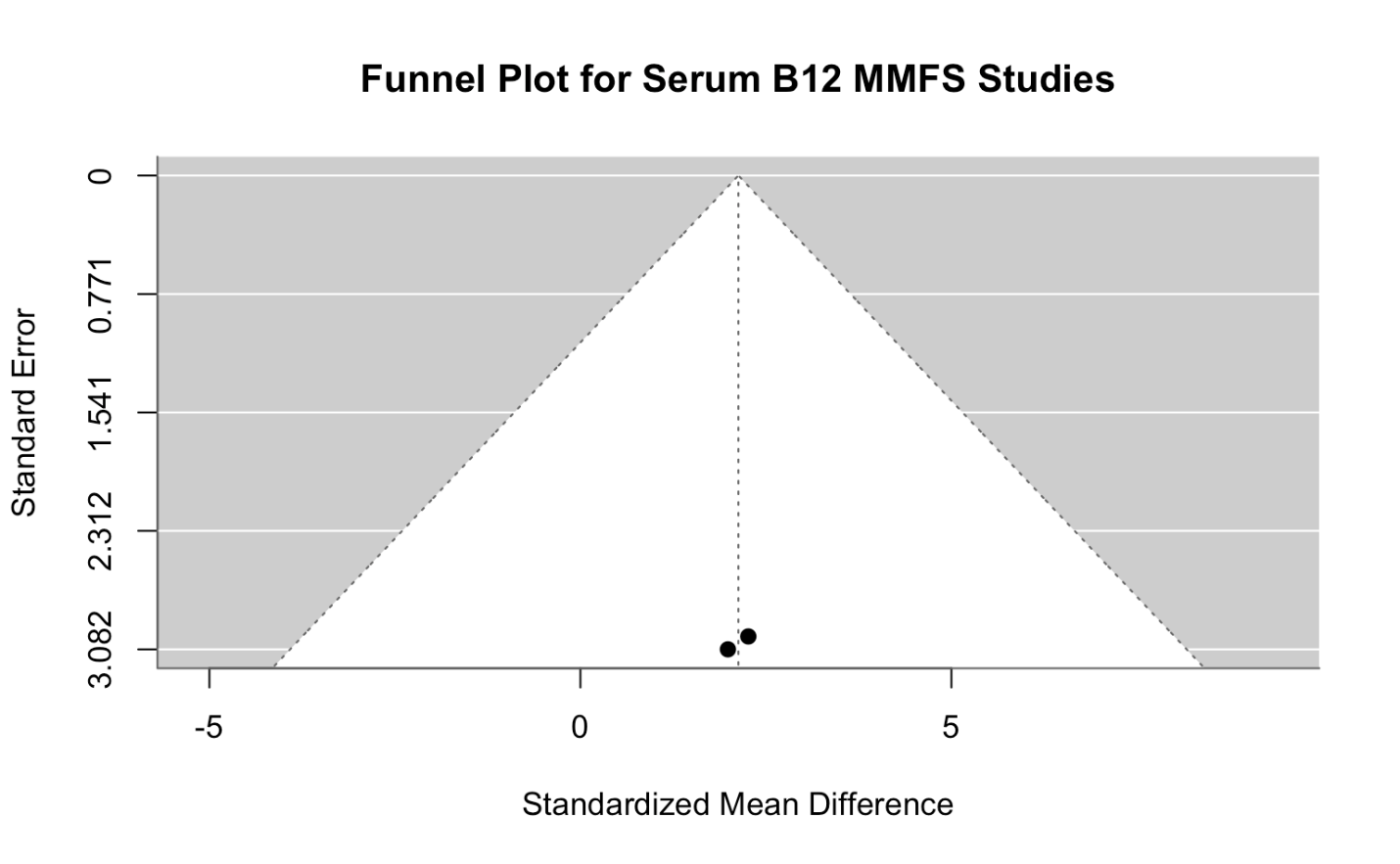


Supplemental Figure 20: Funnel plot for serum zinc concentration standardized mean difference among studies of multiple micronutrient fortified salt


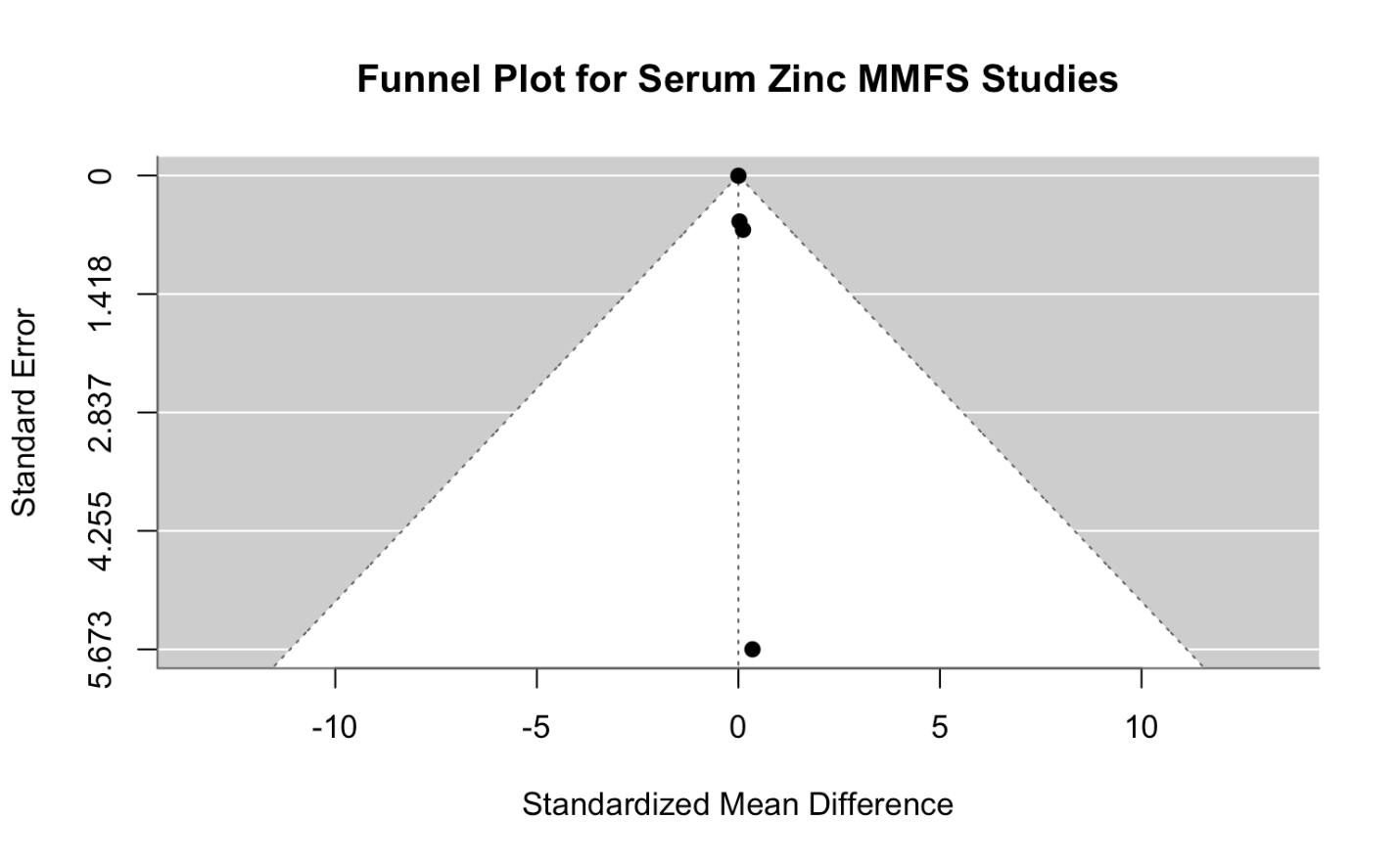


Supplemental Figure 21: Funnel plot for anemia odds ratio among studies of multiple micronutrient fortified salt


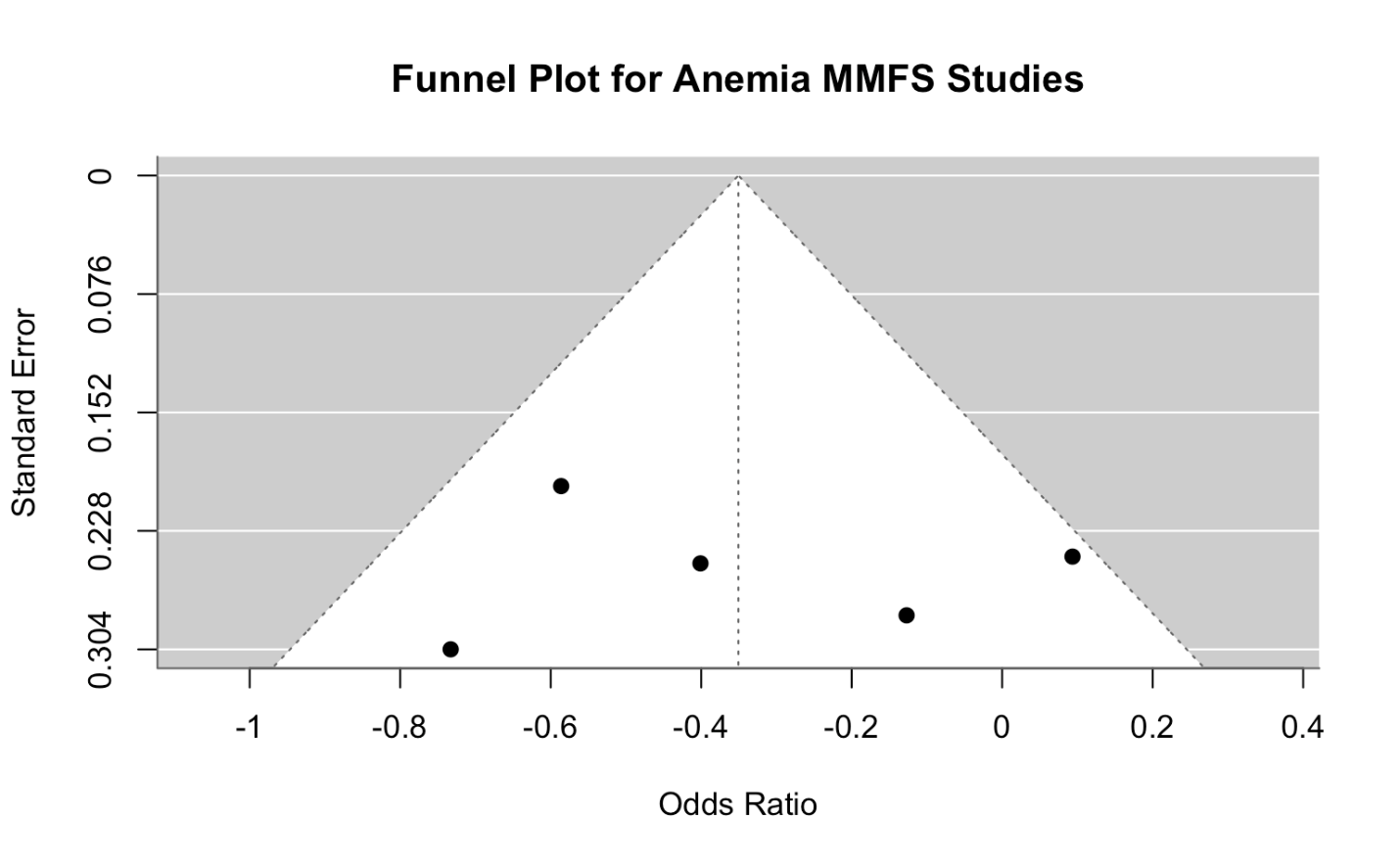


Supplemental Figure 22: Funnel plot for iron deficiency anemia odds ratio among studies of multiple micronutrient fortified salt


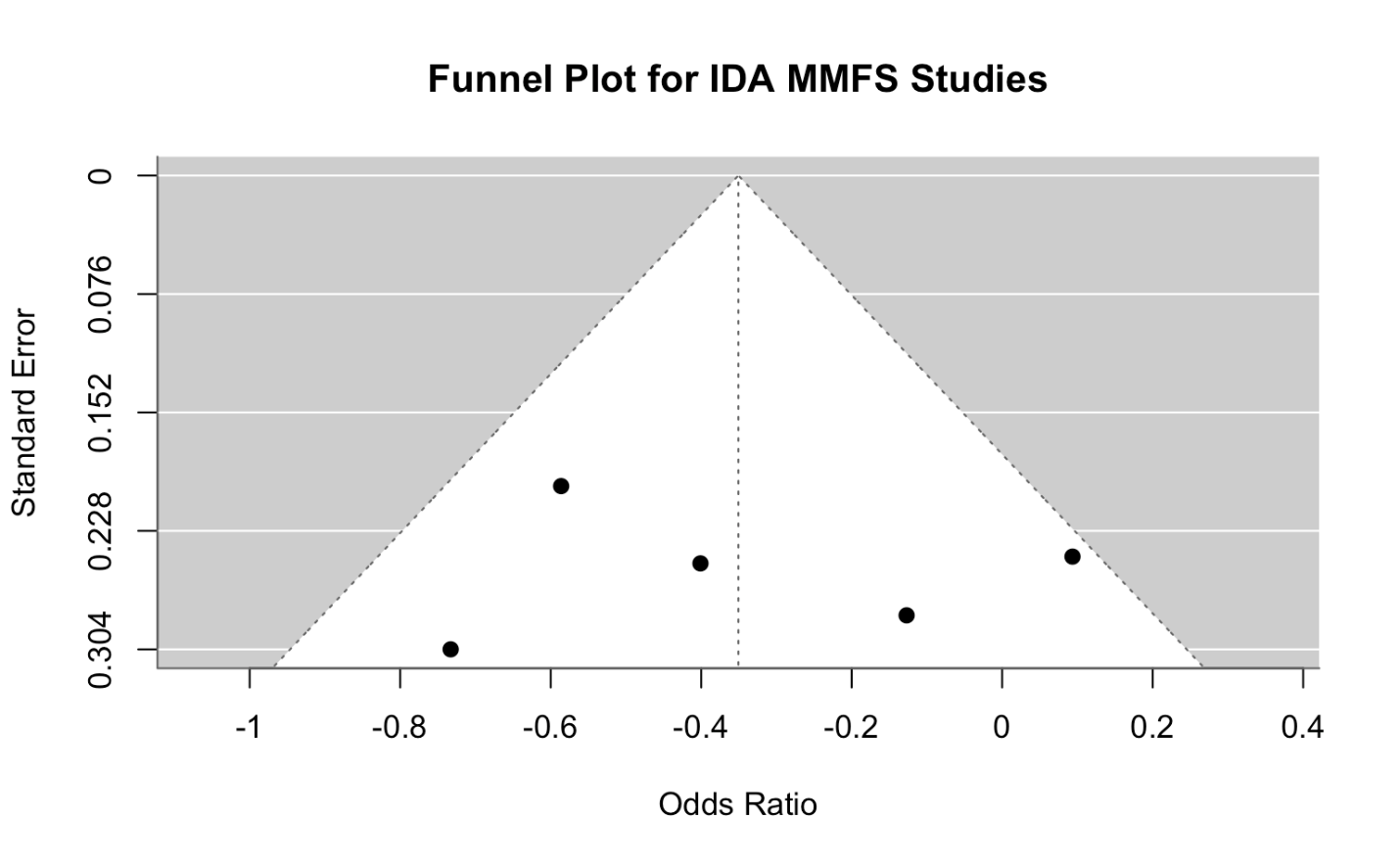


Supplemental Figure 23: Funnel plot for iron deficiency odds ratio among studies of multiple micronutrient fortified salt


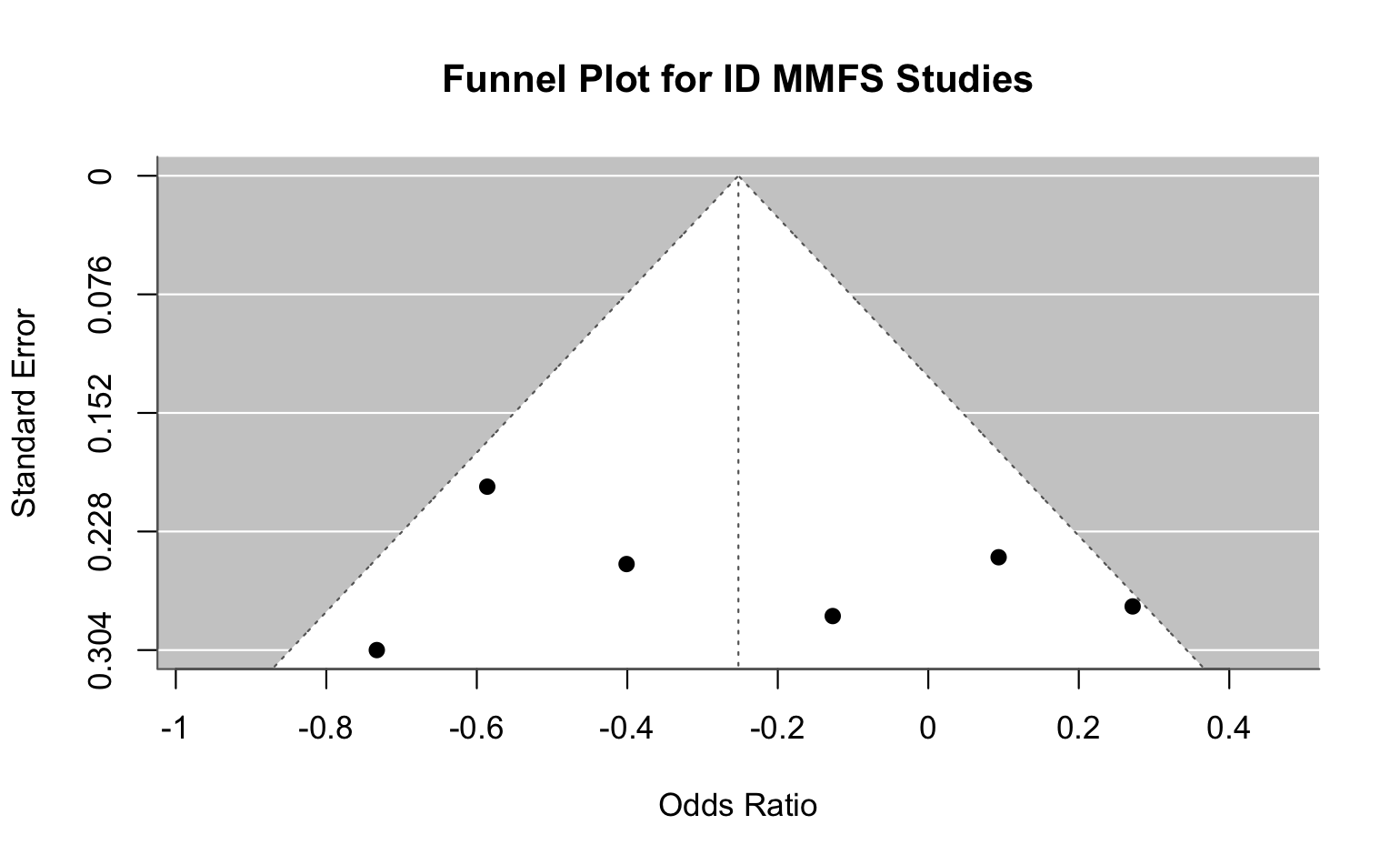


Supplemental Figure 24: Forest plot for effect of double fortified salt on hemoglobin concentration (mean difference)


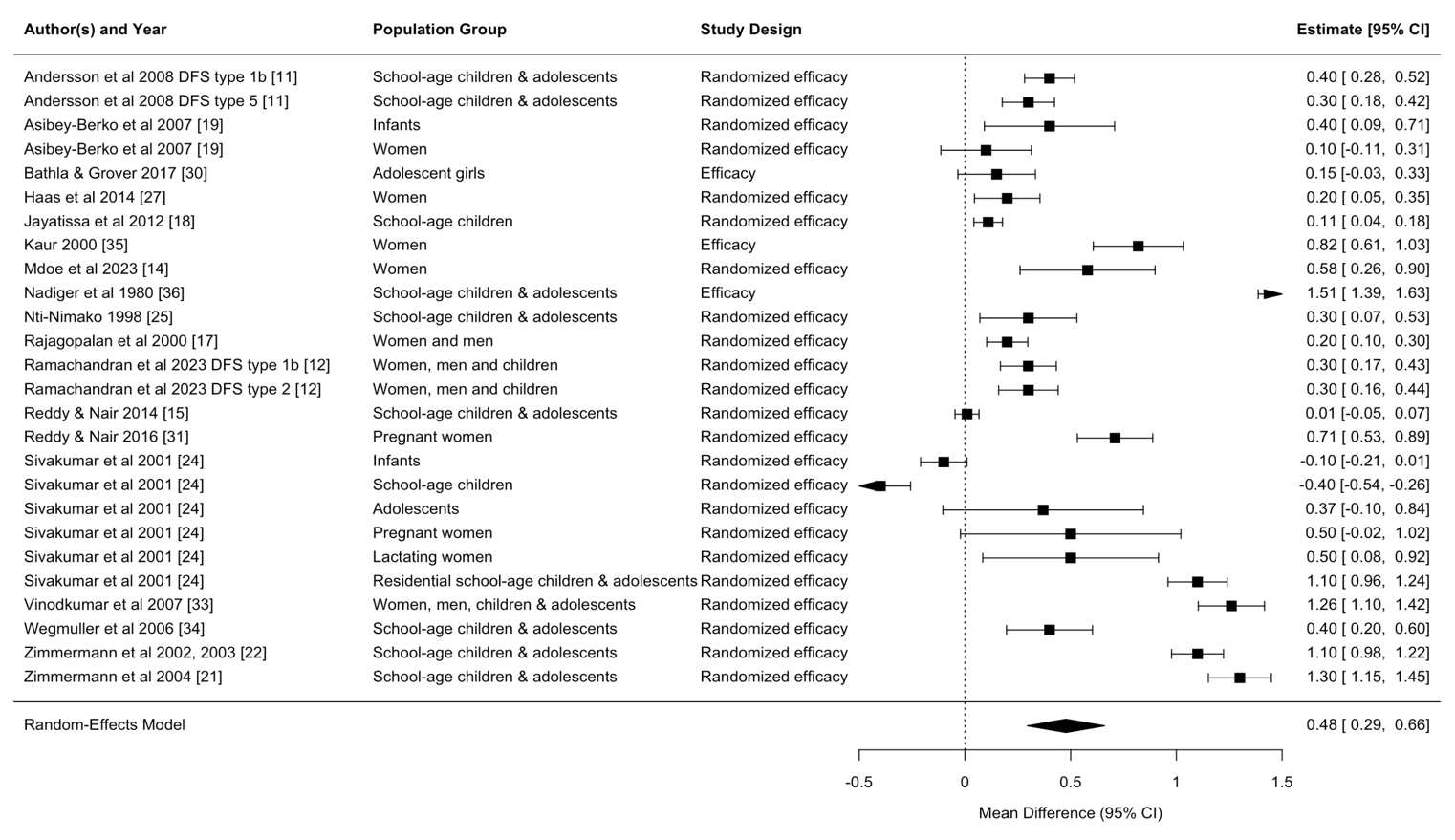


Supplemental Figure 25: Forest plot for effect of double fortified salt on ferritin concentration (standardized mean difference)


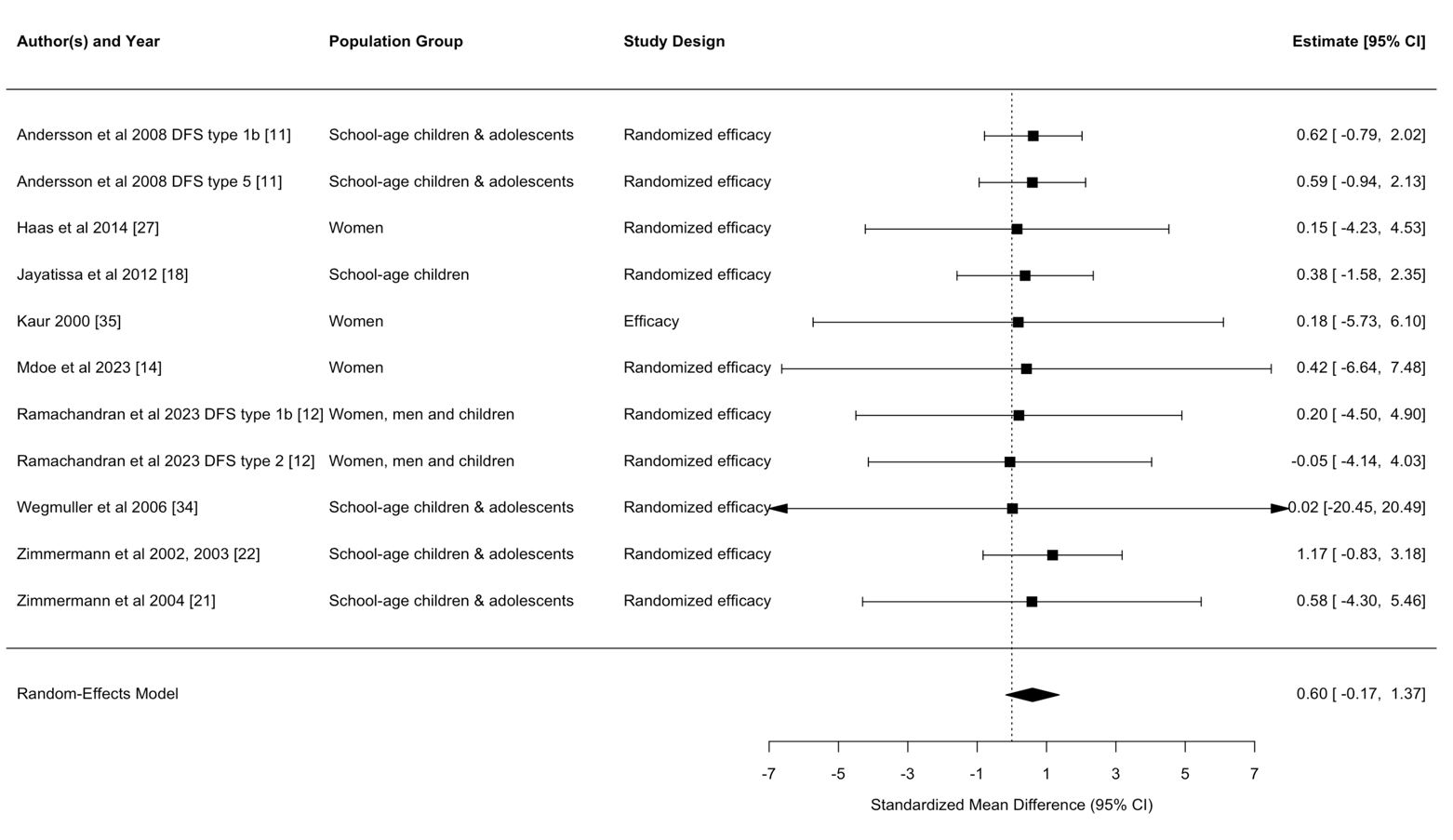


Supplemental Figure 26: Forest plot for effect of double fortified salt on ferritin concentration (mean difference)


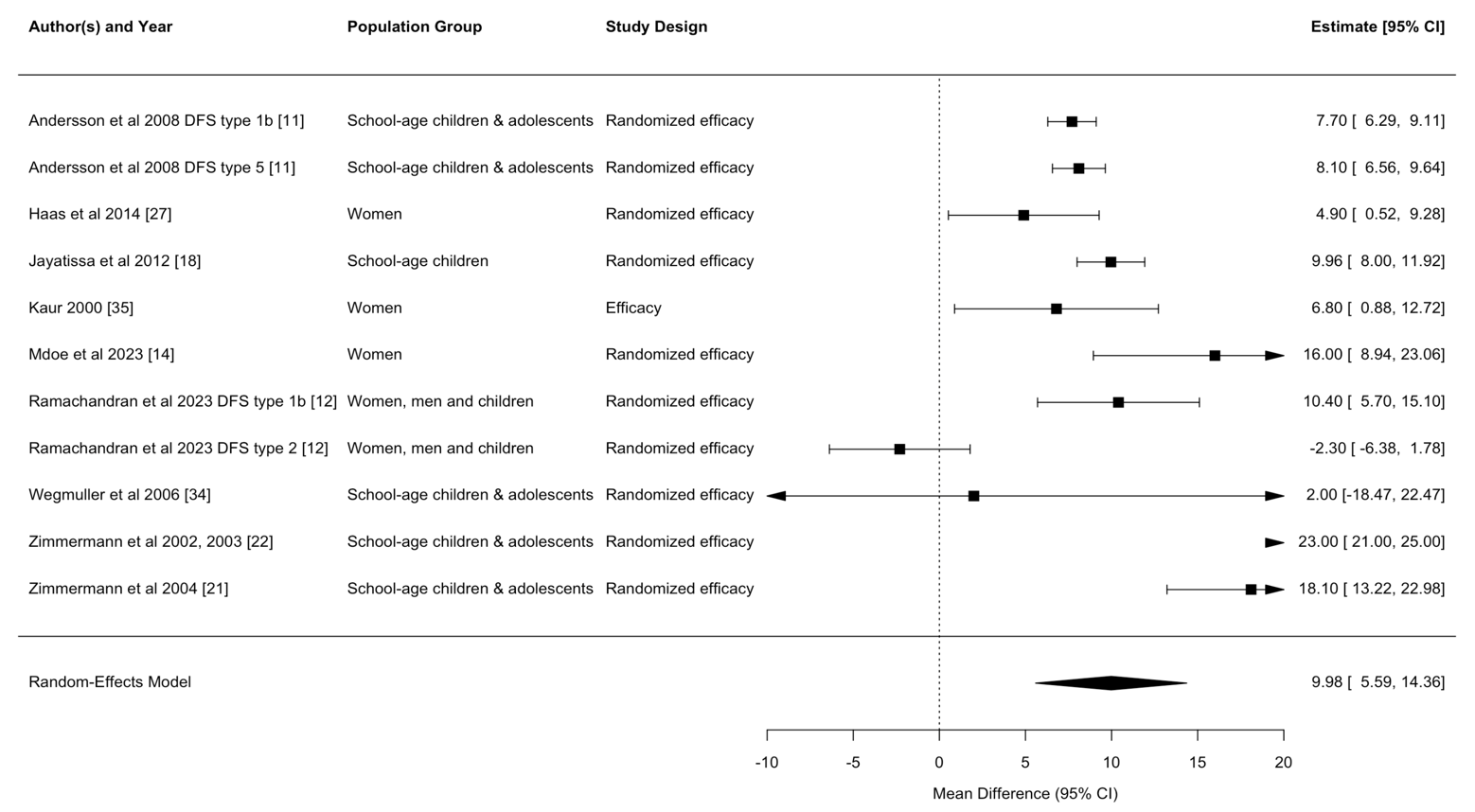


Supplemental Figure 27: Forest plot for effect of double fortified salt on body iron stores (standardized mean difference)


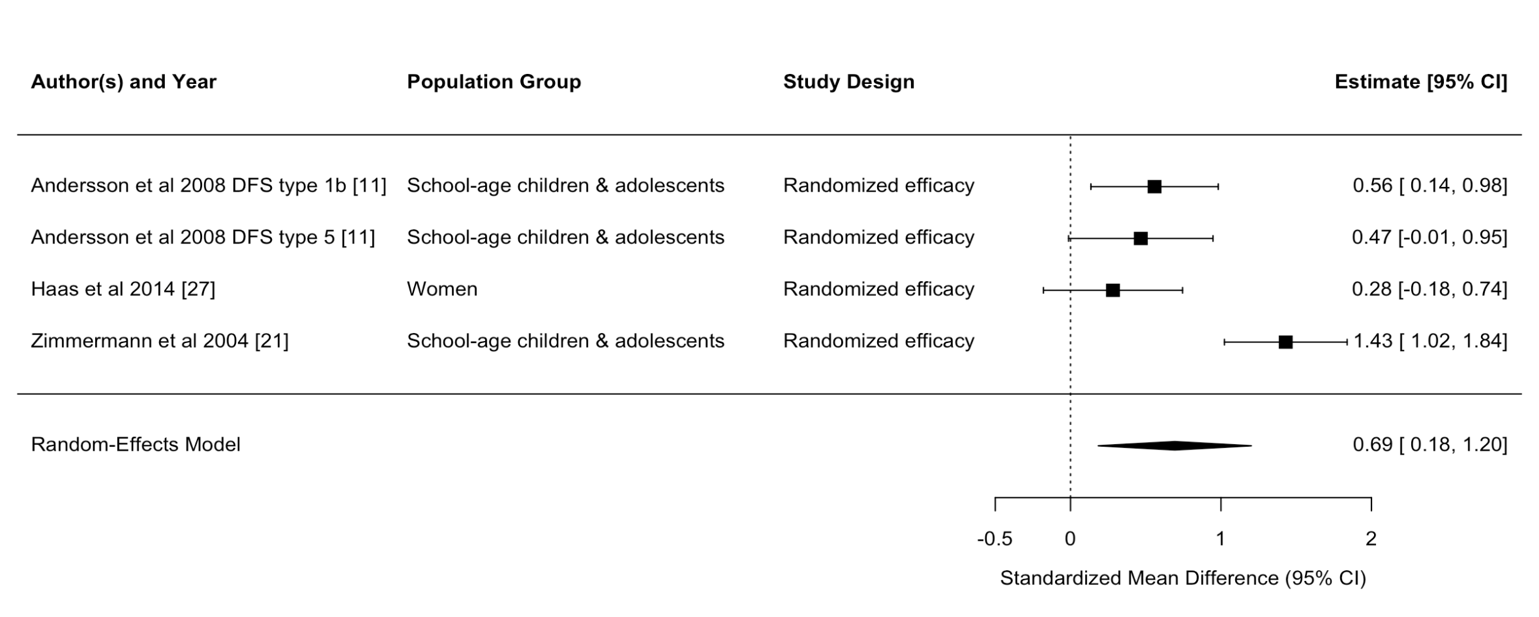


Supplemental Figure 28: Forest plot for effect of double fortified salt on soluble transferrin receptor (standardized mean difference)


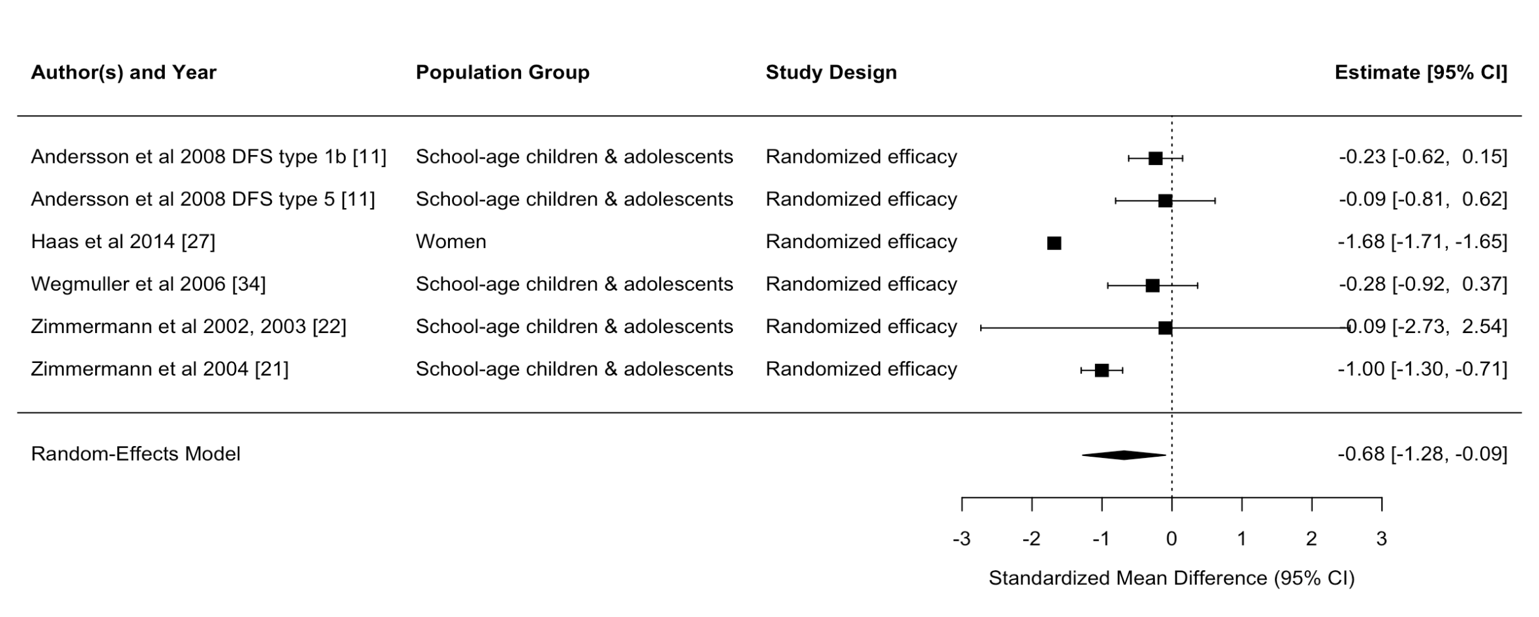


Supplemental Figure 29: Forest plot for effect of double fortified salt on zinc protoporphyrin (standardized mean difference)


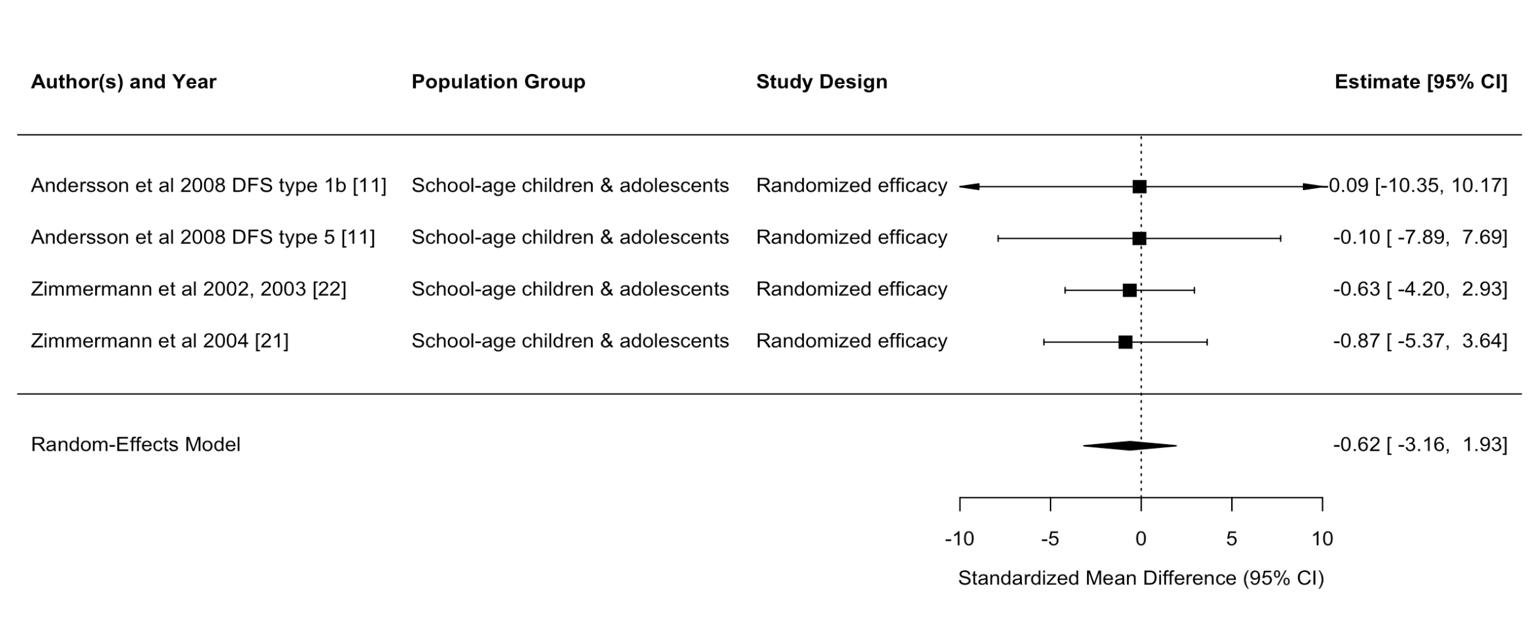


Supplemental Figure 30: Forest plot for effect of salt fortified with folic acid and iodine on folic acid concentration (standardized mean difference)


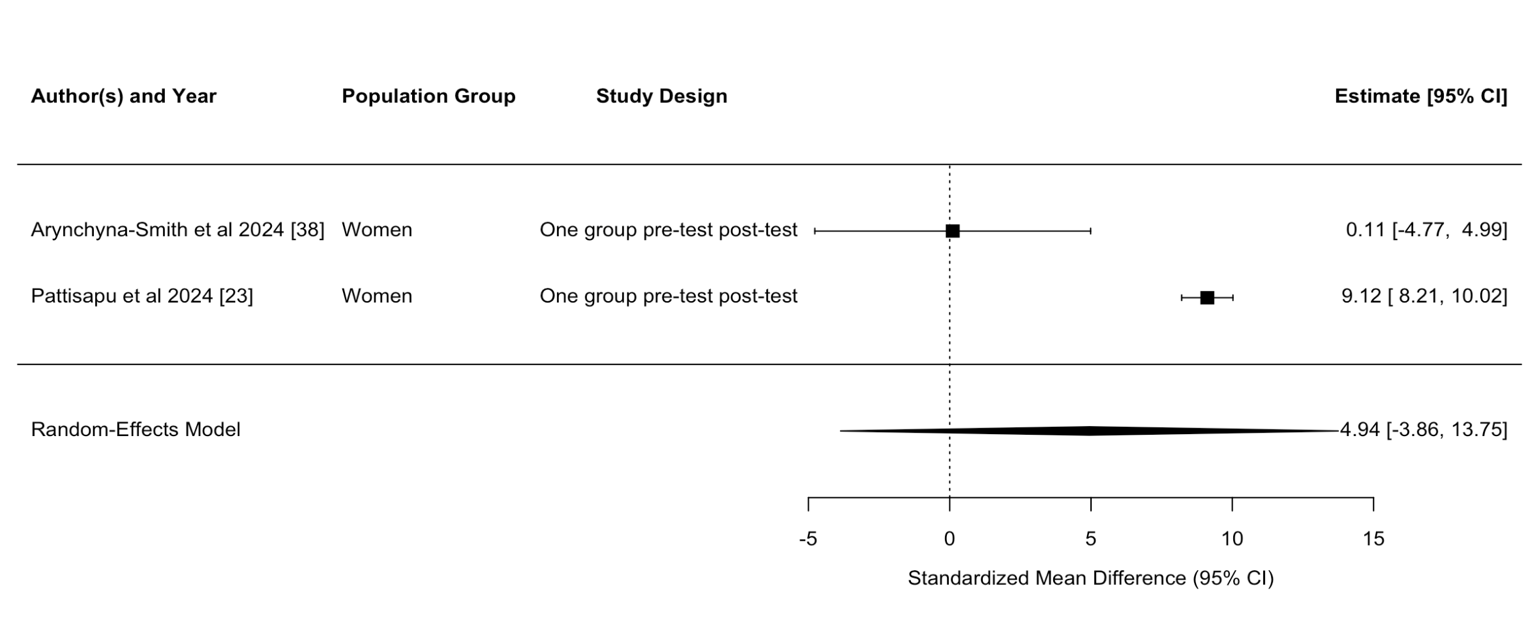


Supplemental Figure 31: Forest plot for effect of multiple micronutrient fortified salt on hemoglobin (unstandardized mean difference)


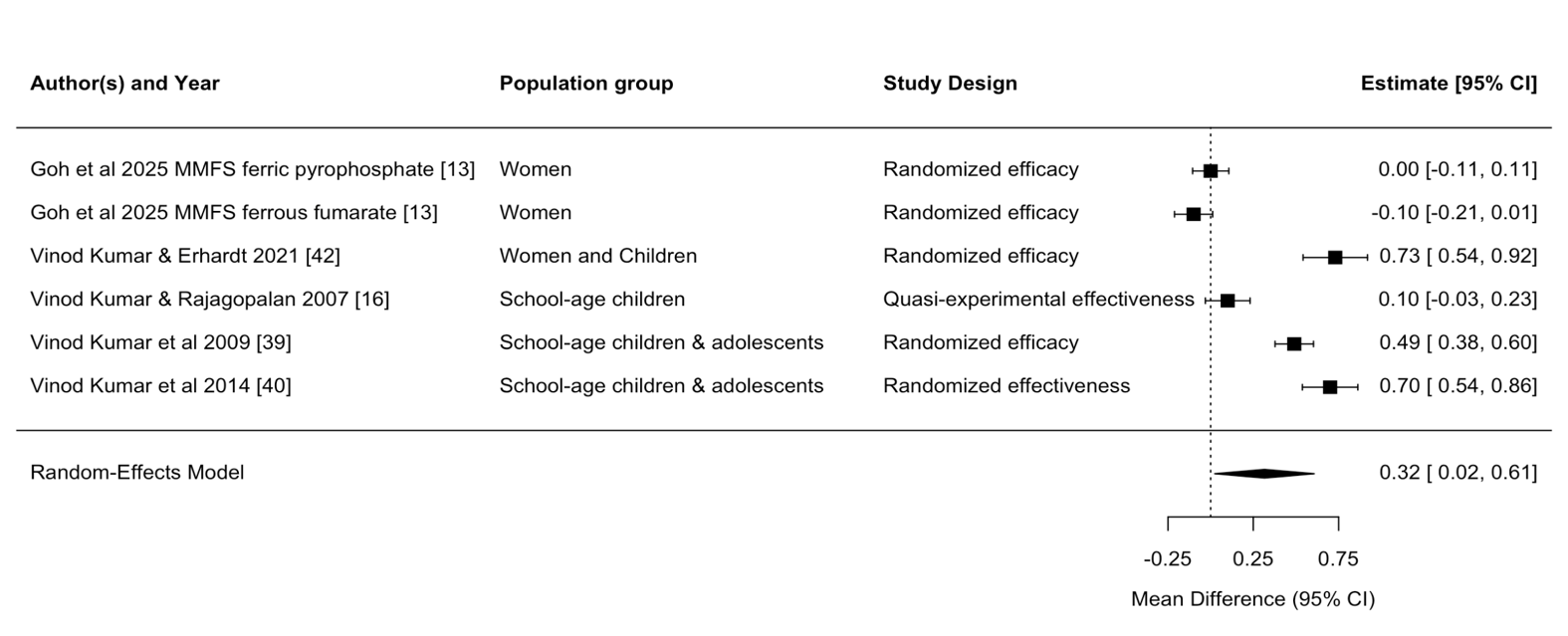


Supplemental Figure 32: Forest plot for effect of multiple micronutrient fortified salt on ferritin concentration (standardized mean difference)


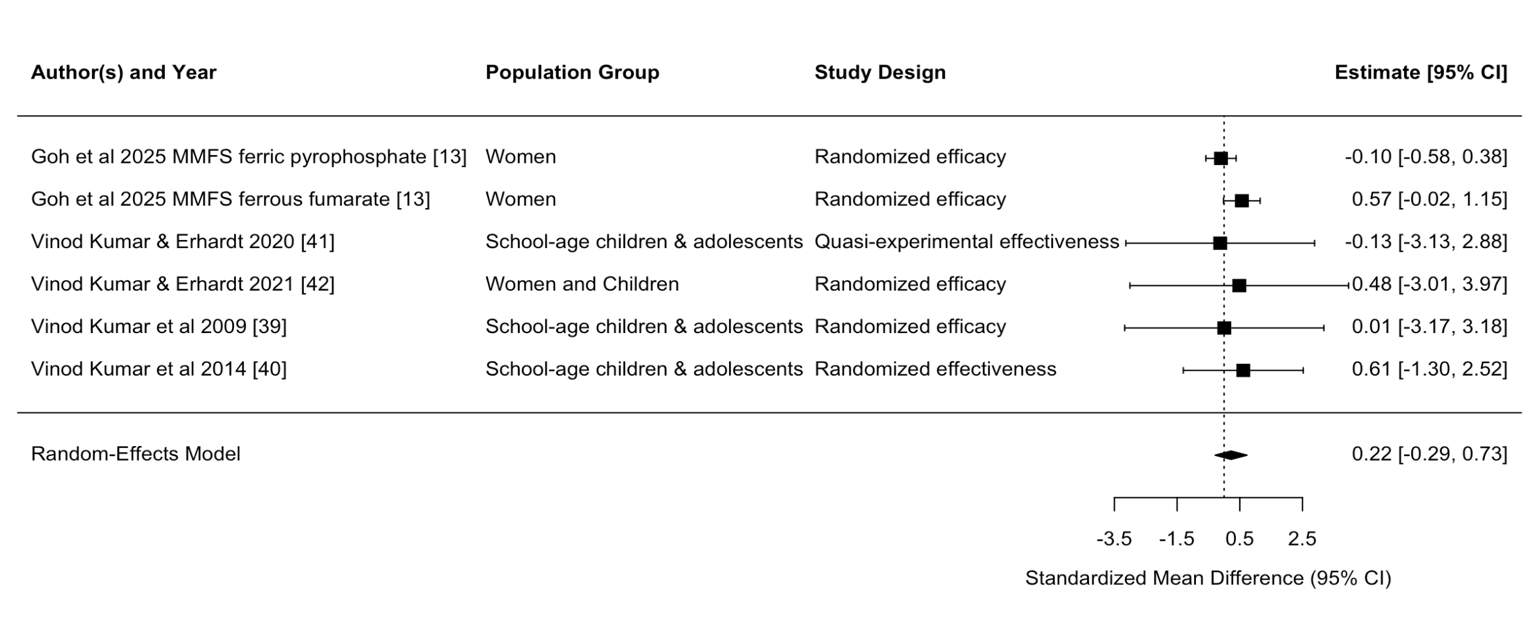


Supplemental Figure 33: Forest plot for effect of multiple micronutrient fortified salt on ferritin concentration (unstandardized mean difference)


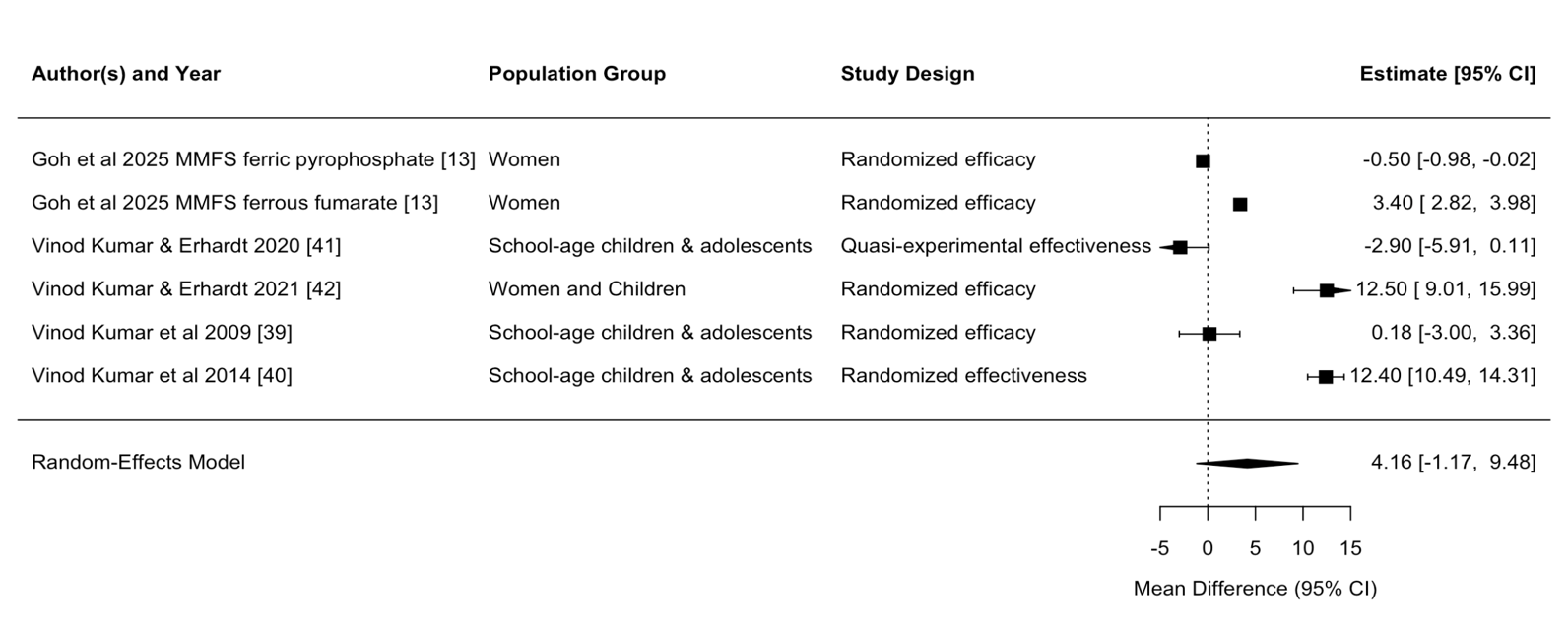


Supplemental Figure 34: Forest plot for effect of multiple micronutrient fortified salt on soluble transferrin receptor (standardized mean difference)


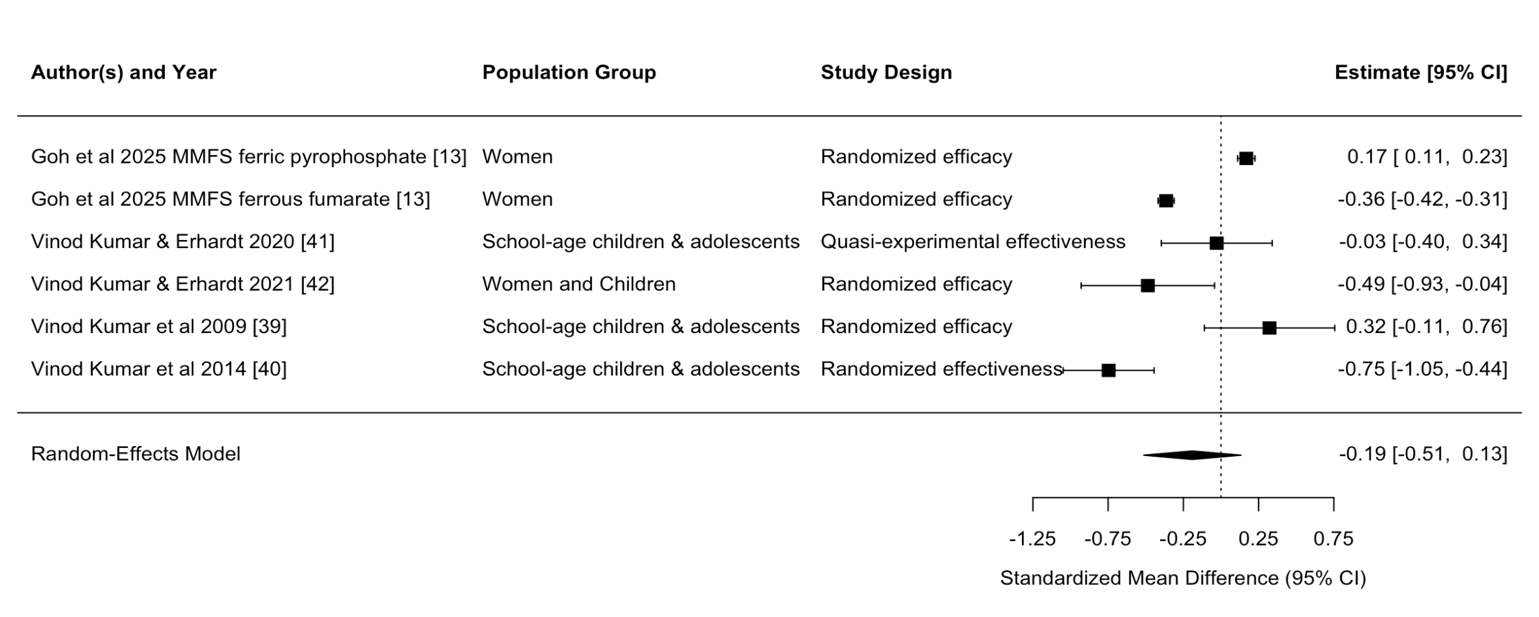


Supplemental Figure 35: Forest plot for effect of multiple micronutrient fortified salt on body iron stores (standardized mean difference)


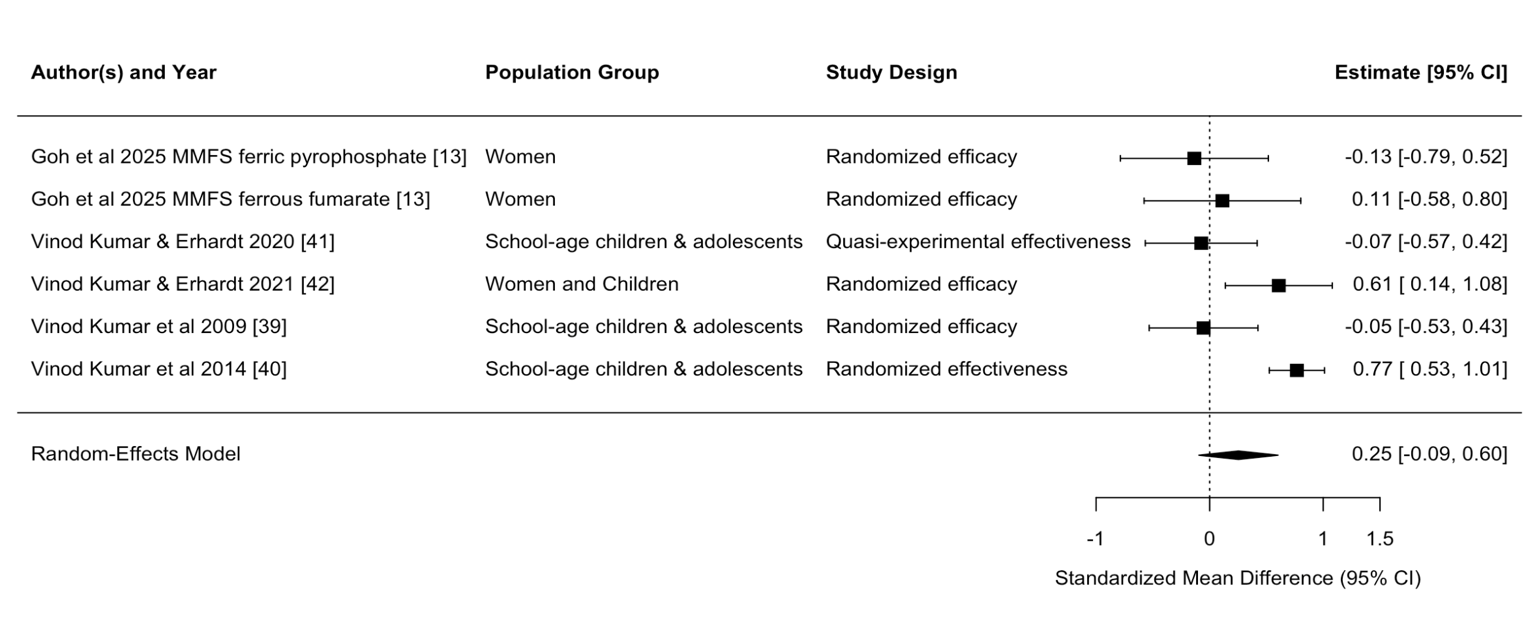


Supplemental Figure 36: Forest plot for effect of multiple micronutrient fortified salt on folic acid concentration (standardized mean difference)


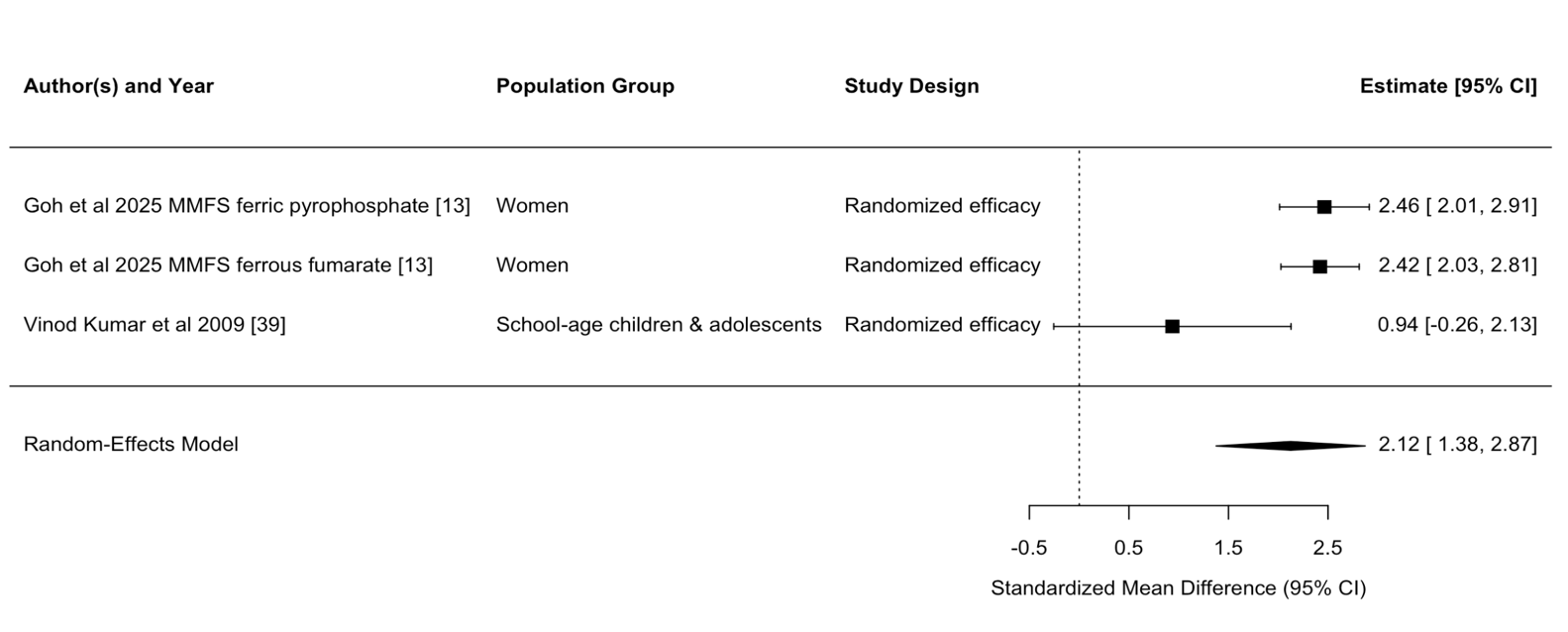


Supplemental Figure 37: Forest plot for effect of multiple micronutrient fortified salt on vitamin B12 concentration (standardized mean difference)


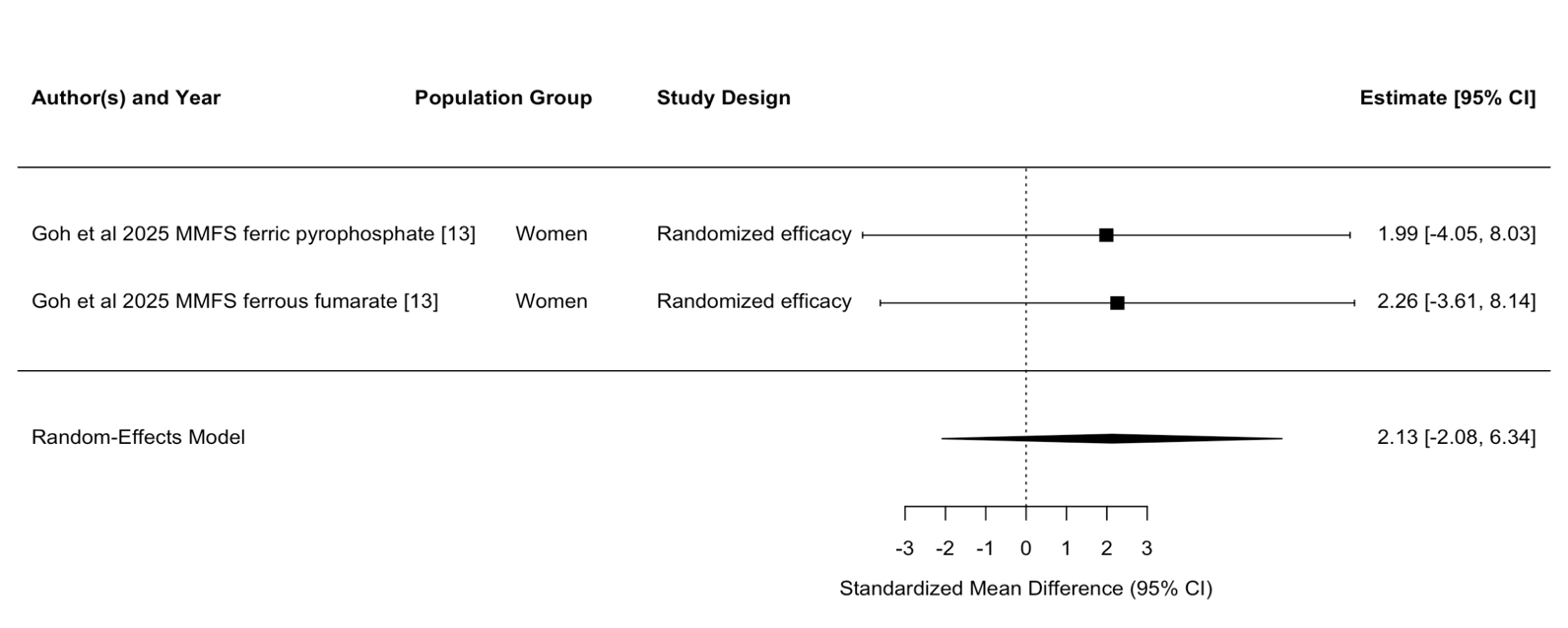


Supplemental Figure 38: Forest plot for effect of multiple micronutrient fortified salt on retinol concentration (standardized mean difference)


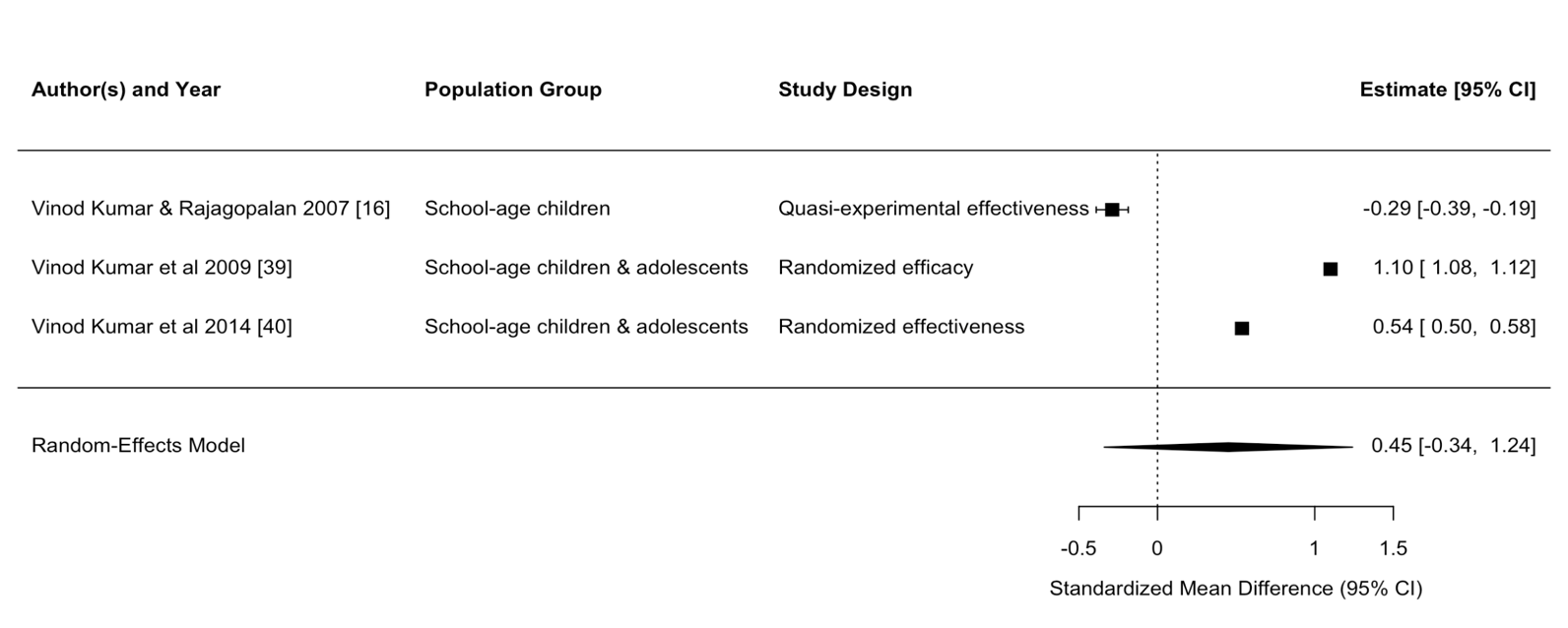


Supplemental Figure 39: Forest plot for effect of multiple micronutrient fortified salt on zinc concentration (standardized mean difference)


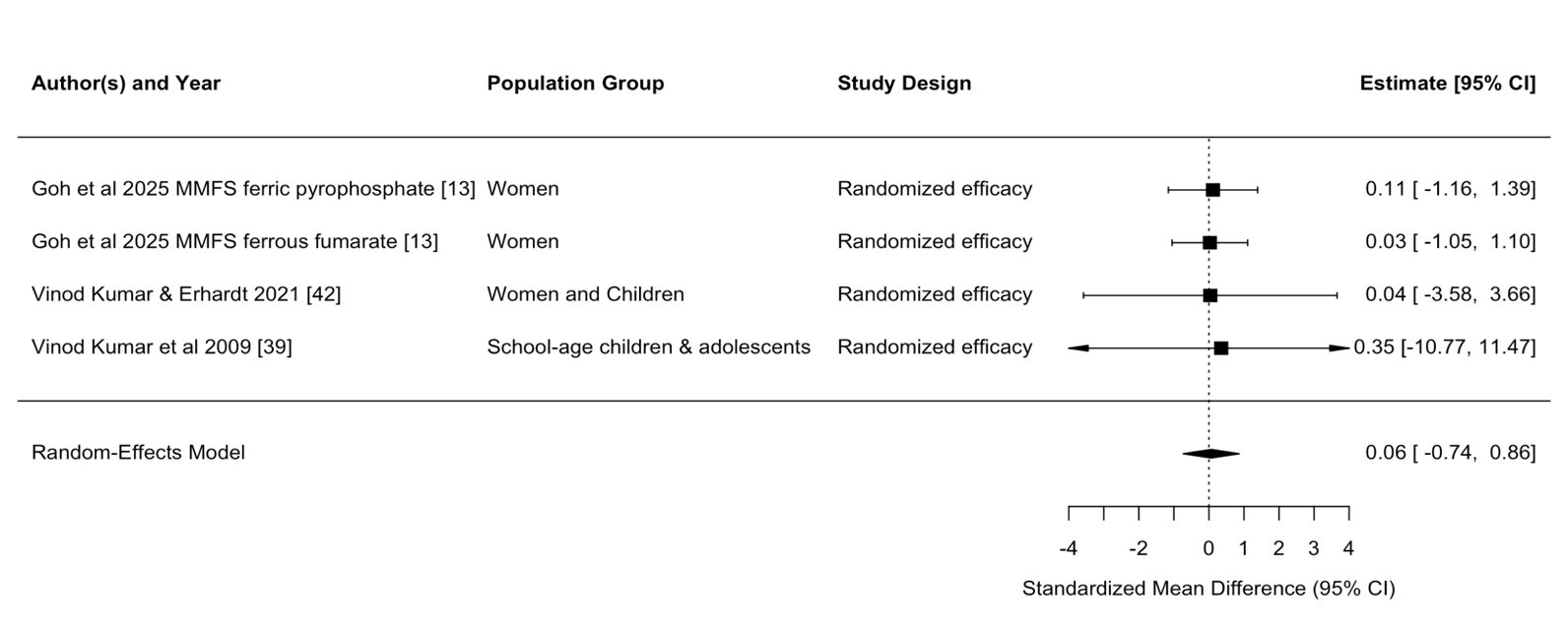


Supplemental Figure 40: Forest plot for effect of multiple micronutrient fortified salt on iron deficiency (odds ratio)


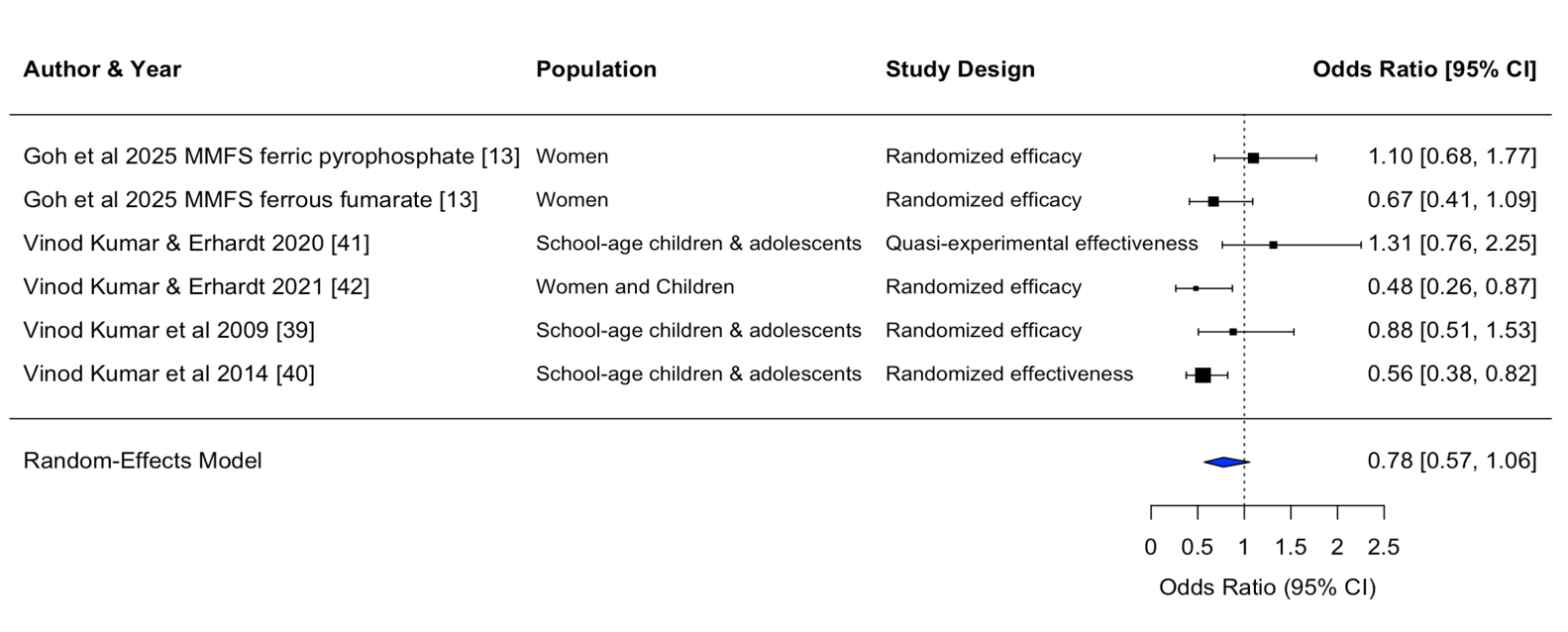

Supplement: Multimedia component 1 [file mmc1.docx]
